# The global, regional, and national burden of benign prostatic hyperplasia in 204 countries and territories from 2000 to 2019: a systematic analysis for the Global Burden of Disease Study 2019

**DOI:** 10.1016/S2666-7568(22)00213-6

**Published:** 2022-11

**Authors:** GBD 2019 Benign Prostatic Hyperplasia Collaborators, Atalel Fentahun Awedew, Hannah Han, Behzad Abbasi, Mohsen Abbasi-Kangevari, Muktar Beshir Ahmed, Omar Almidani, Erfan Amini, Jalal Arabloo, Ayele Mamo Argaw, Seyyed Shamsadin Athari, Daniel Atlaw, Maciej Banach, Amadou Barrow, Akshaya Srikanth Bhagavathula, Vijayalakshmi S Bhojaraja, Boris Bikbov, Belay Boda Abule Bodicha, Nadeem Shafique Butt, Florentino Luciano Caetano dos Santos, Omid Dadras, Xiaochen Dai, Linh Phuong Doan, Sahar Eftekharzadeh, Ali Fatehizadeh, Tushar Garg, Teferi Gebru Gebremeskel, Motuma Erena Getachew, Seyyed-Hadi Ghamari, Syed Amir Gilani, Mahaveer Golechha, Veer Bala Gupta, Vivek Kumar Gupta, Simon I Hay, Mohammad-Salar Hosseini, Mehdi Hosseinzadeh, Ayesha Humayun, Irena M Ilic, Milena D Ilic, Nahlah Elkudssiah Ismail, Mihajlo Jakovljevic, Shubha Jayaram, Seyed Behzad Jazayeri, Alelign Tasew Jema, Ali Kabir, Ibraheem M Karaye, Yousef Saleh Khader, Ejaz Ahmad Khan, Iván Landires, Sang-woong Lee, Shaun Wen Huey Lee, Stephen S Lim, Stany W Lobo, Azeem Majeed, Mohammad-Reza Malekpour, Narges Malih, Ahmad Azam Malik, Entezar Mehrabi Nasab, Tomislav Mestrovic, Irmina Maria Michalek, Gedefaye Nibret Mihrtie, Mohammad Mirza-Aghazadeh-Attari, Awoke Temesgen Misganaw, Ali H Mokdad, Mariam Molokhia, Christopher J L Murray, Sreenivas Narasimha Swamy, Son Hoang Nguyen, Ali Nowroozi, Virginia Nuñez-Samudio, Mayowa O Owolabi, Shrikant Pawar, Norberto Perico, David Laith Rawaf, Salman Rawaf, Reza Rawassizadeh, Giuseppe Remuzzi, Amirhossein Sahebkar, Chethan Sampath, Jeevan K Shetty, Migbar Mekonnen Sibhat, Jasvinder A Singh, Ker-Kan Tan, Gebremaryam Temesgen, Musliu Adetola Tolani, Marcos Roberto Tovani-Palone, Sahel Valadan Tahbaz, Rohollah Valizadeh, Bay Vo, Linh Gia Vu, Lin Yang, Fereshteh Yazdanpanah, Arzu Yigit, Vahit Yiğit, Ismaeel Yunusa, Mazyar Zahir, Theo Vos, M Ashworth Dirac

## Abstract

**Background:**

Benign prostatic hyperplasia is a common urological disease affecting older men worldwide, but comprehensive data about the global, regional, and national burden of benign prostatic hyperplasia and its trends over time are scarce. As part of the Global Burden of Diseases, Injuries, and Risk Factors Study (GBD) 2019, we estimated global trends in, and prevalence of, benign prostatic hyperplasia and disability-adjusted life-years (DALYs) due to benign prostatic hyperplasia, in 21 regions and 204 countries and territories from 2000 to 2019.

**Methods:**

This study was conducted with GBD 2019 analytical and modelling strategies. Primary prevalence data came from claims from three countries and from hospital inpatient encounters from 45 locations. A Bayesian meta-regression modelling tool, DisMod-MR version 2.1, was used to estimate the age-specific, location-specific, and year-specific prevalence of benign prostatic hyperplasia. Age-standardised prevalence was calculated by the direct method using the GBD reference population. Years lived with disability (YLDs) due to benign prostatic hyperplasia were estimated by multiplying the disability weight by the symptomatic proportion of the prevalence of benign prostatic hyperplasia. Because we did not estimate years of life lost associated with benign prostatic hyperplasia, disability-adjusted life-years (DALYs) equalled YLDs. The final estimates were compared across Socio-demographic Index (SDI) quintiles. The 95% uncertainty intervals (UIs) were estimated as the 25th and 975th of 1000 ordered draws from a bootstrap distribution.

**Findings:**

Globally, there were 94·0 million (95% UI 73·2 to 118) prevalent cases of benign prostatic hyperplasia in 2019, compared with 51·1 million (43·1 to 69·3) cases in 2000. The age-standardised prevalence of benign prostatic hyperplasia was 2480 (1940 to 3090) per 100 000 people. Although the global number of prevalent cases increased by 70·5% (68·6 to 72·7) between 2000 and 2019, the global age-standardised prevalence remained stable (–0·770% [–1·56 to 0·0912]). The age-standardised prevalence in 2019 ranged from 6480 (5130 to 8080) per 100 000 in eastern Europe to 987 (732 to 1320) per 100 000 in north Africa and the Middle East. All five SDI quintiles observed an increase in the absolute DALY burden between 2000 and 2019. The most rapid increases in the absolute DALY burden were seen in the middle SDI quintile (94·7% [91·8 to 97·6]), the low-middle SDI quintile (77·3% [74·1 to 81·2]), and the low SDI quintile (77·7% [72·9 to 83·2]). Between 2000 and 2019, age-standardised DALY rates changed less, but the three lower SDI quintiles (low, low-middle, and middle) saw small increases, and the two higher SDI quintiles (high and high-middle SDI) saw small decreases.

**Interpretation:**

The absolute burden of benign prostatic hyperplasia is rising at an alarming rate in most of the world, particularly in low-income and middle-income countries that are currently undergoing rapid demographic and epidemiological changes. As more people are living longer worldwide, the absolute burden of benign prostatic hyperplasia is expected to continue to rise in the coming years, highlighting the importance of monitoring and planning for future health system strain.

**Funding:**

Bill & Melinda Gates Foundation.

**Translation:**

For the Amharic translation of the abstract see Supplementary Materials section.

## Introduction

Benign prostatic hyperplasia is a multifocal, non-malignant, hyperplastic, and progressive histopathological change in stromal and epithelial cells in the transitional zone of the prostate, resulting in discrete prostatic nodules, inflammation, fibrosis, and changes in smooth muscle activity, which can cause partial or complete obstruction of the urethra.^1^, ^2^ The resulting bladder outlet obstruction, coupled with increased muscle tone of the bladder and secondary dysfunction of the detrusor, produce lower urinary tract symptoms.^3^

Benign prostatic hyperplasia is a common urological disease among older men. The age-specific prevalence of benign prostatic hyperplasia has been estimated from autopsy studies to be 8% in the fourth decade of life, 50% in the sixth decade of life, and 80% in the ninth decade of life.^4^, ^5^ The annual prostatic volume increment with age, based on Krimpen and Baltimore's longitudinal study of ageing, is about 2·0–2·5% per year in older men.^6^, ^7^ There is some evidence to suggest the prevalence varies by race and ethnicity.^8^ Other factors associated with benign prostatic hyperplasia include metabolic syndrome, obesity, increased BMI, dyslipidaemia, diabetes, cardiovascular disease, acute and chronic prostatic inflammation, functional bladder capacity, treatment for cardiac disease, post-void residual urine volume, educational level, antidepressant use, calcium antagonist use, erectile function or dysfunction, high concentrations of prostate disease-specific antigen, family history of bladder cancer, and family history of prostatic disease, whereas an inverse association has been observed with increased physical exercise, moderate alcohol consumption, and smoking.^5^, ^8^, ^9^, ^10^, ^11^, ^12^, ^13^, ^14^, ^15^, ^16^, ^17^, ^18^, ^19^, ^20^

**Research in context**

**Evidence before this study**

Given that the global population is both growing and ageing, addressing the burden of age-associated diseases, such as benign prostatic hyperplasia, has become a global health priority. Although a systematic review was not conducted before producing and reporting these estimates, we did identify many studies that have examined the prevalence of benign prostatic hyperplasia in different communities. However, varied case definitions, research methodologies, access to care, diagnostic modalities, and coding practices created a challenge of assessing and comparing the disease burden across different populations and over time. The Global Burden of Diseases, Injuries, and Risk Factors Study (GBD) assembles data from diverse sources worldwide and applies standardised data processing and modelling techniques to facilitate like-versus-like comparisons across time and space. Previous GBD publications on multiple diseases, and a previous study by Launer and colleagues based on GBD 2017 estimates, reported a rising burden of benign prostatic hyperplasia worldwide.

**Added value of this study**

The present study overcomes some of the limitations of epidemiological studies conducted in one or a few locations by making use of the GBD modelling methods to leverage international administrative data and systematically estimate the prevalence of, and disability-adjusted life-years (DALYs) associated with, benign prostatic hyperplasia in 204 countries and territories between 2000 and 2019. We extended and improved upon the results of GBD 2017 reported by Launer and colleagues by providing estimates for additional countries and territories as well as more recent estimation years, incorporating more location-years of prevalence data, applying a novel method to adjust for systematic bias in prevalence data sources, and incorporating the use of a validated instrument to estimate benign prostatic hyperplasia symptom severity. We explored both absolute and age-standardised rates of benign prostatic hyperplasia burden at different levels of geographical hierarchy, over time, and across countries with different Socio-demographic Index (SDI) rankings. We found that the absolute burden of benign prostatic hyperplasia is rising across all SDI quintiles, largely driven by demographic changes, but that the age-standardised rate of benign prostatic hyperplasia appears to be increasing at low SDI quintiles and appears to be decreasing at higher SDI quintiles. As the first formal report of the global benign prostatic hyperplasia burden prepared by the GBD Benign Prostatic Hyperplasia Collaborators, this study also provides the most nuanced discussion to date of the strengths and limitations of the available dataset and current methods for estimation of the benign prostatic hyperplasia burden, which should help guide primary data collection in the future.

**Implications of all the available evidence**

Our study shows that the burden of benign prostatic hyperplasia is increasing in many parts of the world. This increase was most notable in low-income and middle-income countries that are undergoing rapid demographic and epidemiological transitions, highlighting the importance of monitoring and planning for future health system strain. The comprehensive nature of this study provides important policy-relevant information to health-care professionals, policy makers, and international health organisations to assess the burden of benign prostatic hyperplasia at the global, regional, and country levels; prepare effective public health awareness campaigns; and implement effective diagnostic, prevention, and treatment strategies to manage the growing burden of benign prostatic hyperplasia.

Previous studies have shown that benign prostatic hyperplasia contributes to increased health costs^21^ and decreased quality of life.^22^, ^23^, ^24^ It is associated with serious morbidities, including an increased risk of falls, depression, and diminished health-related quality of life based on indicators such as sleep, psychological condition, activities of daily living, and sexual wellbeing.^21^ The effects of benign prostatic hyperplasia are not only seen on the patient but also on the patient's family and on society at large.^21^, ^25^ Beyond its immediate effect on morbidity, benign prostatic hyperplasia is also associated with complications such as urinary tract infection, acute urinary retention, urolithiasis, and acute renal failure.^21^, ^24^, ^26^

Benign prostatic hyperplasia has been identified as a major urological health problem in older men in many countries.^27^, ^28^ The prevalence of benign prostatic hyperplasia from descriptive epidemiology studies ranges from 12% to 42%,^29^, ^30^, ^31^ and one study estimated the lifetime risk of benign prostatic hyperplasia to be 29%.^32^ A systematic review and meta-analysis by Lee and colleagues^29^ in 2017 identified 30 population-based, hospital-based, and community-based epidemiological studies in different countries, and yielded a 26% point prevalence of benign prostatic hyperplasia in older men for the years 1990–2016. Another meta-analysis done in China indicated that the pooled overall prevalence of benign prostatic hyperplasia among men aged 40 years or older was 36·6% during 1989–2014.^33^

The Global Burden of Diseases, Injuries, and Risk Factors Study (GBD) is the largest and most comprehensive scientific effort to produce estimates of health loss due to 369 diseases and injuries. As such, GBD overcomes some of the limitations of the epidemiological studies described above by utilising a large number of administrative datasets from around the world, processing them all in a comparable fashion, and using regional patterns and predictive covariates to provide the most precise annual estimates of disease burden possible for a large number of countries and territories, including those without primary data, over a long time series. The burden of benign prostatic hyperplasia has been estimated and included in comprehensive reports since GBD 2010,^34^, ^35^, ^36^, ^37^, ^38^, ^39^ and Launer and colleagues^40^ previously reported the global benign prostatic hyperplasia burden in GBD 2017. In this report, we extended and improved upon the GBD 2017 results reported by Launer and colleagues by providing estimates for additional countries and territories and more recent estimation years, incorporating more location-years of prevalence data, applying a novel method to adjust for systematic bias in prevalence data sources, and incorporating the use of a validated instrument to estimate benign prostatic hyperplasia symptom severity. We also provided a more detailed account of the dataset and current methods for estimation of the benign prostatic hyperplasia burden to stimulate substantive engagement on data collection priorities and future directions for improvement of estimations.

With the population rapidly ageing in many parts of the world, the burden of benign prostatic hyperplasia is expected to rise. Understanding the current burden of and recent trends in benign prostatic hyperplasia, the role of demographic and other factors in driving the change, and the strengths and limitations of existing datasets is necessary to fill data gaps and help health systems prepare for the challenges associated with this rising global burden.

This manuscript was produced as part of the GBD Collaborator Network and in accordance with the GBD Protocol.

## Methods

### Overview

A comprehensive description of GBD study aims, data sources, methodologies, and analytical tools has been reported previously.^41^ The methods specific to the estimation of health loss due to benign prostatic hyperplasia are summarised below. The analysis presented here complies with the Guidelines for Accurate and Transparent Health Estimates Reporting (GATHER) statement. All input data and the code used to execute all data processing and modelling described below can be found on the Global Health Data Exchange (GHDx) website.

### Case definition

In this study, we defined a case of benign prostatic hyperplasia on the basis of an individual receiving that diagnosis in a clinical encounter, as ascertained from administrative data, using the codes of the International Classification of Diseases, version 9 (ICD-9), and version 10 (ICD-10).^42^ The ICD-9 codes used were 600, 600.0, 600.1, 600.2, 600.3, and 600.9, and the ICD-10 codes used were N40, N40.0, N40.1, N40.2, N40.3, and N40.9. Ascertainment via diagnostic codes in administrative data necessarily means that not all individuals have had a histological diagnosis of benign prostatic hyperplasia, and receipt of clinical care suggests these individuals could be properly described as having lower urinary tract symptoms due to benign prostatic obstruction; for consistency with previous GBD publications and visualisations, we refer to these as cases of benign prostatic hyperplasia in this Article. Complications of benign prostatic hyperplasia that can be mapped to other GBD-defined diseases, such as urinary tract infections, kidney stones, and chronic kidney disease, were not included in the analysis. Only non-fatal health loss was estimated in this analysis, because life-threatening complications of benign prostatic hyperplasia are classified in other GBD-defined diseases and mortality due to these complications should not be double-counted.

### Prevalence data sources

**Figure 1:**
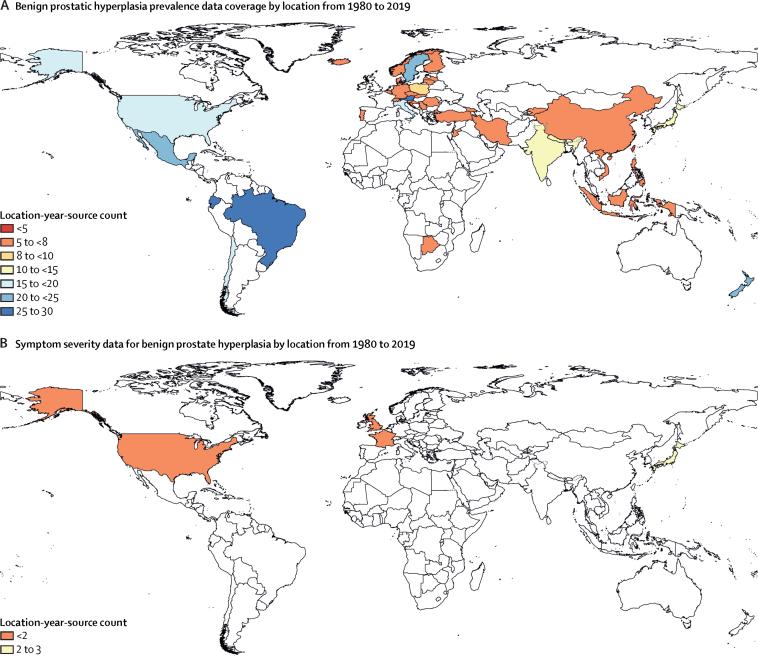
Data coverage for estimating prevalence of benign prostatic hyperplasia (A) Location-years of prevalence data for benign prostatic hyperplasia. These data are combined with Bayesian priors, predictive covariates, and regional patterns to produce estimates of prevalence specific to year, age group, and location. (B) Location-years of symptom severity data for benign prostatic hyperplasia. Due to sparsity, these data were pooled to produce a single estimate of symptomatic versus asymptomatic benign prostatic hyperplasia, which is applied to year-age-location-specific estimates of overall benign prostatic hyperplasia prevalence to determine the prevalence of symptomatic benign prostatic hyperplasia on which to apply disability.

We used international clinical administrative data to estimate the prevalence of benign prostatic hyperplasia. Clinical administrative data included claims from three locations—the USA, Taiwan (province of China), and Poland—and hospital inpatient admissions data from 45 locations. The claims data from the USA consisted of more than 12 billion claims records from the commercially insured population in 2000 and 2010–16. Claims data from Taiwan (province of China) were from the national insurance programme covering more than 99% of the population in 2016, and claims data from Poland were from the national insurance programme covering more than 90% of the population in 2015–17.^43^, ^44^, ^45^ The inpatient admission records came from 297 sources, each covering 1–5 years of data between 1980 and 2018 (figure 1). A complete list of prevalence data sources is available in appendix 2 (pp 22–30) and on the GHDx website.

### Prevalence data processing

The processing of these data sources has been described in detail elsewhere.^41^ Briefly, for claims data, we used the unique enrollee identification numbers to link inpatient and outpatient claims to a single individual. An enrollee was then extracted as a prevalent case if they had at least one inpatient or two outpatient medical encounters with any of the defining ICD codes for benign prostatic hyperplasia in either the primary or secondary diagnostic position. Processing of inpatient admissions data involved converting benign prostatic hyperplasia admission counts into cause fractions, which are the number of hospital admissions specifically for benign prostatic hyperplasia divided by the total number of admissions in the data year. Then, these cause fractions were multiplied by the hospital admission rate per capita and the total population size for each unique source, age, sex, and year combination to convert data to population-level inpatient admission rates. Details of the data and methods used to estimate hospital admission rates and population sizes have been previously described.^41^ Population-level benign prostatic hyperplasia admission rates were then transformed to population prevalence data by applying a ratio of total benign prostatic hyperplasia cases to inpatient benign prostatic hyperplasia admissions modelled from claims data.

We treated claims data and outpatient-adjusted inpatient admission data from Taiwan (province of China) and Poland as reference data, meeting our ICD-based case definition, and representing a general population defined only by year, age, sex and geographical location. The claims data from the USA, however, were adjusted to account for selection bias since these data come from a database of commercial insurance claims, and enrolment in commercial health insurance is generally associated with higher economic status. The adjustment coefficient was estimated with a Bayesian, regularised, trimmed meta-regression (MR-BRT) analysis. MR-BRT is a mixed-effects meta-regression tool that accounts for between-study heterogeneity and has been described previously.^41^, ^46^, ^47^ Once all data were standardised to the reference standard, and the uncertainty of the adjustment process was accounted for, we applied the median absolute deviation (MAD) exclusion criterion to systematically exclude unreasonably high or low datapoints as outliers. Specifically, this was done by calculating the MAD of the age-standardised prevalence of all data and marking any data series greater than two MAD from the median as outliers and excluding them from the analysis (8·8% of processed datapoints were marked as outliers on the basis of the MAD exclusion criterion).

### Prevalence modelling

We used DisMod-MR version 2.1 to estimate the age-specific, year-specific, and location-specific prevalence of benign prostatic hyperplasia. DisMod-MR is a Bayesian mixed-effects meta-regression tool that was designed for disease modelling by the Institute for Health Metrics and Evaluation (Seattle, WA, USA).^41^, ^48^, ^49^ The tool uses a compartmental model with a series of age-integrated differential equations to estimate a set of epidemiological measures (prevalence, incidence, remission, excess mortality rate, relative risk, and cause-specific mortality rate) that are internally consistent with one another. Estimation occurs at each level of a geographical cascade (ie, global, seven GBD super-regions, 21 regions, and 204 countries and territories), in which each subsequent model borrows information from the previous model in the cascade via a Bayesian prior.^41^ A Gaussian data likelihood function is used at each step in the cascade. We provided value priors on incidence, remission, and excess mortality. First, we assumed a benign prostatic hyperplasia incidence of zero in men younger than 40 years. The maximum disease duration above the age of 40 years was set at 10 years, and we assumed excess mortality rates of zero for all ages. We included age-standardised prevalence of diabetes as a predictive covariate because of its known association with benign prostatic hyperplasia. In previous rounds of GBD, we had tested mean BMI as a predictive covariate. This was dropped in GBD 2019, however, because it did not have a significant association with benign prostatic hyperplasia and did not improve prediction.^50^, ^51^, ^52^, ^53^, ^54^

The estimates from the most granular level of location, either at the country or subnational level, were aggregated up along the geographical cascade to obtain the final regional, super-regional, and global prevalence of benign prostatic hyperplasia. Age-specific prevalence was estimated for 12 age groups: 40–44 years, 45–49 years, 50–54 years, 55–59 years, 60–64 years, 65–69 years, 70–74 years, 75–79 years, 80–84 years, 85–89 years, 90–94 years, and 90 years and older. Age-standardised prevalence was estimated across all age groups through the direct method and the GBD reference population.^55^ Input data, outlier designations, and model fit for the benign prostatic hyperplasia DisMod MR model can be viewed on the Epi Visualization Viz Hub.

### Disability weight and severity distribution

The disability weight represents the severity of health loss associated with a generic health state, described in lay language. It ranges from 0 (perfect health) to 1 (death). Disability weights for generic health states were estimated by use of nine large population-based surveys and one open-access internet survey where participants were asked to compare pairwise combinations of health states.^56^, ^57^, ^58^ Two health states were assigned to benign prostatic hyperplasia: asymptomatic and symptomatic. The symptomatic health state has a disability weight of 0·067 (95% CI 0·043–0·097). The asymptomatic health state has a disability weight of zero, assuming no 95% CIs.^35^

To determine what proportion of the estimated benign prostatic hyperplasia prevalence to assign to the symptomatic health state, we used data from four community-based surveys from Japan, the USA, Scotland, and France that recruited men aged 40–84 years.^59^ The surveys used the International Prostate Symptom Score (I-PSS), a validated questionnaire, to measure the severity of lower urinary tract symptoms among men.^22^, ^60^, ^61^ We modelled the cumulative distribution of the I-PSS scores in survey participants using MR-BRT to estimate the mean proportion of individuals with symptomatic lower urinary tract symptoms. The symptomatic and asymptomatic proportions were applied to the prevalence estimates from DisMod-MR to produce estimates of the prevalence of symptomatic and asymptomatic benign prostatic hyperplasia.

### Years lived with disability (YLDs) and disability-adjusted life-years (DALYs)

YLDs due to benign prostatic hyperplasia were estimated by multiplying the disability weights by the prevalence of symptomatic and asymptomatic benign prostatic hyperplasia and summing them together. Because we did not estimate mortality for benign prostatic hyperplasia, we did not produce years of life lost (YLLs) for benign prostatic hyperplasia. Therefore, DALYs equalled YLDs. Final estimates by year, age, and location, including those included in the text, figures, tables, and appendix 2 of this Article, can be viewed on the GBD Compare Viz Hub.

### Socio-demographic Index

The Socio-demographic Index (SDI) is a summary measure that describes a country's development. The SDI is derived from a country's total fertility rate for women younger than 25 years, educational attainment in adults, and lag-distributed income per capita. Details of the SDI estimation methods are available elsewhere.^62^ We grouped all GBD countries and territories in five SDI quintiles on the basis of their SDI values in 2019. For this analysis, we used the most recent year's SDI groupings when describing trends between 2000 and 2019. The list of locations and their assigned SDI quintile can be found in appendix 2 (pp 8–13).

The 95% uncertainty intervals (UIs) were estimated by taking 1000 draws of the distribution of every modelling and computation process. The final mean estimate was calculated by taking the mean value of the 1000 draws, and the 95% UI was set by finding the 25th and 975th of their ordered values. Data are presented to three significant figures.

### Role of the funding source

The funder of the study had no role in study design, data collection, data analysis, data interpretation, or the writing of the report.

## Results

In 2019, there were 94·0 million (95% UI 73·2 to 118) prevalent cases of benign prostatic hyperplasia globally among men aged 40 years and older, corresponding to an age-standardised prevalence of 2480 (1940 to 3090) per 100 000 (table). This was a 70·5% (95% UI 68·6 to 72·7) increase from 51·1 million (43·1 to 69·3) cases in 2000. The global age-standardised prevalence, however, remained largely unchanged during this period (–0·770% [–0·0912 to 1·56] difference).

**Table:** Global, GBD super-region, and country-level prevalence of benign prostatic hyperplasia, and percentage change between 2000 and 2019

			**2000**		**2019**		**Percentage change between 2000 and 2019 (%)**	
			Cases (95% UI)	Age-standardised prevalence per 100 000 (95% UI)	Cases (95% UI)	Age-standardised prevalence per 100 000 (95% UI)	Cases (95% UI)	Age-standardised prevalence (95% UI)
**Global**			**55 100 000 (43 100 000 to 69 300 000)**	**2500 (1960 to 3120)**	**94 000 000 (73 200 000 to 118 000 000)**	**2480 (1940 to 3090)**	**70·5% (68·6 to 72·7)**	**−0·770% (−1·56 to 0·0912)**
**Central Europe, eastern Europe, and central Asia**			**9 830 000 (7 770 000 to 12 300 000)**	**4890 (3900 to 6000)**	**12 200 000 (9 670 000 to 15 200 000)**	**4800 (3840 to 5910)**	**23·8% (20·2 to 27·4)**	**−1·86% (−4·30 to 0·410)**
	Central Asia		511 000 (394 000 to 664 000)	2550 (1980 to 3240)	754 000 (579 000 to 994 000)	2590 (2020 to 3310)	47·7% (39·7 to 56·1)	1·71% (−1·33 to 4·70)
		Armenia	35 500 (26 500 to 46 700)	2540 (1930 to 3300)	46 400 (35 100 to 60 800)	2600 (1980 to 3380)	30·5% (20·8 to 40·6)	2·28% (−4·22 to 8·84)
		Azerbaijan	58 300 (43 600 to 78 400)	2530 (1920 to 3320)	100 000 (75 900 to 134 000)	2590 (1980 to 3380)	72·3% (57·7 to 87·3)	2·53% (−4·50 to 8·83)
		Georgia	77 600 (65 300 to 90 800)	2970 (2520 to 3460)	74 400 (63 300 to 86 600)	3050 (2610 to 3540)	−4·24% (−10·6 to 2·61)	2·61% (−3·85 to 9·58)
		Kazakhstan	117 000 (88 200 to 155 000)	2500 (1910 to 3260)	168 000 (127 000 to 224 000)	2560 (1960 to 3350)	43·6% (32·0 to 55·6)	2·35% (−5·61 to 10·2)
		Kyrgyzstan	27 100 (20 300 to 35 800)	2130 (1610 to 2800)	38 600 (29 400 to 51 300)	2160 (1650 to 2830)	42·6% (28·6 to 58·7)	1·42% (−5·48 to 9·70)
		Mongolia	11 200 (8490 to 14 900)	2480 (1890 to 3250)	22 200 (16 800 to 29 800)	2540 (1920 to 3310)	97·2% (81·1 to 114)	2·25% (−5·36 to 10·0)
		Tajikistan	32 600 (24 600 to 43 000)	2510 (1900 to 3280)	53 900 (40 100 to 71 200)	2550 (1930 to 3320)	65·5% (47·6 to 84·8)	1·80% (−5·40 to 9·73)
		Turkmenistan	22 300 (16 600 to 29 800)	2520 (1890 to 3300)	39 600 (29 800 to 52 200)	2560 (1950 to 3340)	77·7% (63·0 to 93·3)	1·81% (−5·27 to 10·4)
		Uzbekistan	129 000 (96 600 to 172 000)	2510 (1900 to 3280)	211 000 (156 000 to 284 000)	2580 (1950 to 3370)	63·6% (48·6 to 80·5)	2·70% (−3·86 to 10·4)
	Central Europe		2 230 000 (1 840 000 to 2 700 000)	3160 (2620 to 3810)	2 970 000 (2 440 000 to 3 600 000)	3140 (2590 to 3770)	32·9% (29·9 to 36·5)	−0·684% (−2·65 to 1·48)
		Albania	37 800 (28 500 to 50 300)	3000 (2270 to 3940)	64 200 (48 500 to 85 100)	3000 (2290 to 3970)	70·0% (59·7 to 82·7)	0·238% (−5·14 to 7·26)
		Bosnia and Herzegovina	65 000 (48 600 to 85 400)	3060 (2340 to 3970)	84 000 (63 700 to 111 000)	3090 (2360 to 4070)	29·3% (18·2 to 41·1)	0·982% (−6·93 to 9·42)
		Bulgaria	186 000 (141 000 to 248 000)	3080 (2340 to 4030)	196 000 (150 000 to 258 000)	3060 (2360 to 4010)	5·32% (−2·58 to 12·5)	−0·493% (−7·40 to 5·85)
		Croatia	90 400 (76 600 to 106 000)	2990 (2560 to 3500)	116 000 (100 000 to 135 000)	2990 (2590 to 3480)	28·1% (19·3 to 37·9)	0·0853% (−6·75 to 7·46)
		Czechia	252 000 (194 000 to 311 000)	3970 (3080 to 4860)	380 000 (295 000 to 475 000)	3990 (3080 to 4940)	50·8% (38·6 to 62·9)	0·566% (−7·25 to 7·72)
		Hungary	197 000 (150 000 to 258 000)	3080 (2380 to 4020)	245 000 (186 000 to 321 000)	3070 (2350 to 3990)	24·8% (15·5 to 35·9)	−0·286% (−7·39 to 7·98)
		Montenegro	10 400 (7800 to 13 800)	3070 (2300 to 4040)	13 800 (10 400 to 18 200)	3070 (2340 to 4010)	33·3% (23·9 to 42·8)	−0·0975% (−7·46 to 7·10)
		North Macedonia	32 500 (24 300 to 42 900)	3090 (2330 to 4040)	48 300 (36 600 to 63 600)	3100 (2380 to 4040)	48·7% (37·7 to 60·8)	0·274% (−6·49 to 8·26)
		Poland	518 000 (429 000 to 626 000)	2600 (2160 to 3100)	777 000 (644 000 to 936 000)	2600 (2170 to 3110)	49·9% (45·5 to 54·1)	0·105% (−1·89 to 2·02)
		Romania	505 000 (423 000 to 599 000)	3640 (3070 to 4280)	592 000 (496 000 to 698 000)	3660 (3080 to 4310)	17·2% (8·91 to 25·3)	0·598% (−5·94 to 6·98)
		Serbia	220 000 (187 000 to 261 000)	3530 (3040 to 4160)	271 000 (231 000 to 317 000)	3560 (3040 to 4150)	23·1% (14·3 to 32·9)	0·625% (−6·22 to 7·96)
		Slovakia	91 700 (81 700 to 103 000)	3360 (3000 to 3750)	135 000 (120 000 to 153 000)	3350 (2980 to 3760)	47·6% (38·1 to 57·2)	−0·278% (−6·51 to 6·10)
		Slovenia	28 500 (24 400 to 33 500)	2400 (2070 to 2810)	45 700 (38 900 to 54 300)	2370 (2010 to 2800)	60·6% (48·6 to 72·4)	−1·61% (−8·77 to 5·27)
	Eastern Europe		7 080 000 (5 460 000 to 8 910 000)	6490 (5100 to 8050)	8 450 000 (6 600 000 to 10 700 000)	6480 (5130 to 8080)	19·3% (14·5 to 23·9)	−0·0578% (−3·39 to 3·15)
		Belarus	326 000 (256 000 to 412 000)	6250 (4920 to 7780)	367 000 (285 000 to 468 000)	6230 (4890 to 7830)	12·6% (5·35 to 19·4)	−0·243% (−6·31 to 5·86)
		Estonia	49 500 (38 700 to 61 600)	6290 (4980 to 7770)	63 200 (49 300 to 78 800)	6320 (4940 to 7890)	27·7% (19·7 to 35·8)	0·385% (−5·57 to 6·04)
		Latvia	82 500 (69 800 to 96 200)	6300 (5350 to 7300)	93 500 (79 400 to 108 000)	6370 (5420 to 7390)	13·2% (5·01 to 20·3)	1·08% (−5·71 to 7·01)
		Lithuania	130 000 (110 000 to 151 000)	6890 (5900 to 7920)	148 000 (125 000 to 170 000)	6910 (5830 to 7940)	13·9% (7·75 to 20·4)	0·227% (−5·15 to 5·44)
		Moldova	118 000 (92 900 to 150 000)	6290 (4970 to 7900)	147 000 (114 000 to 185 000)	6290 (4950 to 7900)	24·1% (17·4 to 31·5)	−0·0508% (−5·50 to 5·78)
		Russia	4 590 000 (3 520 000 to 5 790 000)	6510 (5040 to 8100)	5 780 000 (4 500 000 to 7 430 000)	6510 (5110 to 8130)	25·9% (18·6 to 33·1)	−0·0348% (−5·33 to 5·48)
		Ukraine	1 790 000 (1 380 000 to 2 270 000)	6460 (5030 to 8080)	1 850 000 (1 440 000 to 2 330 000)	6450 (5030 to 8050)	3·56% (−2·44 to 9·82)	−0·224% (−5·09 to 4·81)
**High income**			**11 700 000 (9 620 000 to 14 400 000)**	**1880 (1550 to 2290)**	**17 600 000 (14 400 000 to 21 500 000)**	**1840 (1520 to 2240)**	**49·5% (47·2 to 52·1)**	**−2·16% (−3·54 to −0·880)**
	Australasia		271 000 (204 000 to 357 000)	1980 (1500 to 2600)	476 000 (359 000 to 624 000)	1990 (1510 to 2600)	75·5% (63·6 to 89·0)	0·277% (−6·67 to 7·74)
		Australia	223 000 (163 000 to 300 000)	1940 (1430 to 2600)	393 000 (286 000 to 530 000)	1950 (1430 to 2600)	76·5% (62·2 to 92·8)	0·567% (−7·77 to 10·0)
		New Zealand	48 800 (41 300 to 58 000)	2220 (1890 to 2640)	83 500 (70 800 to 99 400)	2200 (1870 to 2600)	71·1% (57·5 to 84·9)	−0·905% (−8·67 to 6·81)
	High-income Asia Pacific		1 530 000 (1 160 000 to 2 010 000)	1230 (943 to 1620)	2 380 000 (1 810 000 to 3 110 000)	1180 (906 to 1550)	55·8% (48·4 to 64·6)	−3·92% (−6·37 to −1·48)
		Brunei	664 (488 to 920)	1230 (903 to 1680)	1540 (1140 to 2090)	1190 (869 to 1580)	132% (110 to 156)	−4·00% (−12·4 to 5·61)
		Japan	1 320 000 (1 010 000 to 1 720 000)	1250 (967 to 1630)	1 870 000 (1 430 000 to 2 440 000)	1210 (940 to 1590)	41·8% (34·7 to 50·3)	−3·18% (−5·24 to −1·14)
		Singapore	15 800 (11 600 to 21 300)	1130 (832 to 1520)	42 600 (31 100 to 57 700)	1090 (799 to 1460)	170% (147 to 193)	−3·18% (−11·1 to 4·46)
		South Korea	193 000 (141 000 to 267 000)	1130 (825 to 1530)	467 000 (340 000 to 625 000)	1110 (807 to 1470)	142% (118 to 167)	−1·58% (−10·7 to 7·92)
	High-income North America		3 080 000 (2 730 000 to 3 520 000)	1750 (1550 to 2000)	5 190 000 (4 570 000 to 5 960 000)	1800 (1590 to 2060)	68·8% (65·6 to 71·9)	2·93% (1·42 to 4·44)
		Canada	319 000 (238 000 to 435 000)	1760 (1320 to 2390)	567 000 (421 000 to 767 000)	1770 (1320 to 2390)	78·0% (64·3 to 92·3)	0·707% (−6·44 to 8·36)
		Greenland	363 (272 to 495)	1740 (1300 to 2330)	660 (495 to 903)	1750 (1320 to 2370)	81·6% (66·4 to 96·5)	0·504% (−6·54 to 7·56)
		USA	2 760 000 (2 460 000 to 3 120 000)	1750 (1560 to 1980)	4 630 000 (4 130 000 to 5 250 000)	1800 (1620 to 2030)	67·7% (64·6 to 70·9)	3·20% (1·73 to 4·61)
	Southern Latin America		304 000 (222 000 to 410 000)	1250 (909 to 1680)	461 000 (337 000 to 627 000)	1240 (911 to 1680)	51·8% (43·1 to 63·2)	−0·0610% (−5·64 to 7·28)
		Argentina	197 000 (143 000 to 265 000)	1180 (865 to 1580)	278 000 (204 000 to 375 000)	1170 (865 to 1570)	40·9% (30·7 to 55·3)	−1·17% (−8·25 to 8·43)
		Chile	84 900 (61 900 to 116 000)	1450 (1050 to 1990)	157 000 (112 000 to 216 000)	1430 (1020 to 1960)	85·1% (67·8 to 101)	−1·68% (−10·5 to 6·78)
		Uruguay	21 700 (15 800 to 29 300)	1160 (849 to 1560)	26 100 (19 200 to 35 200)	1150 (846 to 1550)	20·1% (11·3 to 31·0)	−0·623% (−7·80 to 7·81)
	Western Europe		6 560 000 (5 210 000 to 8 220 000)	2270 (1810 to 2820)	9 040 000 (7 240 000 to 11 200 000)	2250 (1800 to 2790)	37·8% (34·7 to 41·2)	−0·975% (−3·06 to 1·21)
		Andorra	712 (529 to 952)	1640 (1220 to 2190)	1140 (850 to 1530)	1610 (1200 to 2150)	60·0% (45·5 to 74·5)	−1·37% (−10·4 to 6·91)
		Austria	246 000 (220 000 to 273 000)	4560 (4100 to 5060)	363 000 (326 000 to 401 000)	4580 (4110 to 5080)	47·8% (39·2 to 57·0)	0·428% (−5·64 to 6·47)
		Belgium	286 000 (185 000 to 363 000)	3750 (2430 to 4750)	369 000 (235 000 to 478 000)	3600 (2310 to 4690)	28·8% (6·85 to 43·4)	−3·91% (−20·1 to 6·96)
		Cyprus	7900 (6340 to 9640)	1620 (1310 to 1970)	15 400 (12 300 to 18 800)	1590 (1270 to 1940)	95·3% (80·2 to 114)	−2·32% (−9·41 to 6·48)
		Denmark	64 700 (45 500 to 85 000)	1730 (1220 to 2280)	94 600 (65 300 to 126 000)	1720 (1210 to 2290)	46·3% (33·1 to 61·5)	−0·398% (−8·90 to 9·56)
		Finland	137 000 (121 000 to 152 000)	3990 (3530 to 4420)	229 000 (201 000 to 256 000)	3970 (3490 to 4430)	67·4% (56·6 to 78·7)	−0·331% (−6·58 to 6·06)
		France	666 000 (485 000 to 894 000)	1620 (1190 to 2160)	948 000 (704 000 to 1 270 000)	1610 (1190 to 2160)	42·4% (31·3 to 54·9)	−0·913% (−8·35 to 6·81)
		Germany	1 000 000 (715 000 to 1 370 000)	1630 (1170 to 2200)	1 380 000 (1 000 000 to 1 890 000)	1620 (1180 to 2230)	36·9% (25·0 to 50·6)	−0·723% (−8·60 to 8·81)
		Greece	139 000 (102 000 to 187 000)	1620 (1190 to 2160)	165 000 (124 000 to 218 000)	1600 (1190 to 2150)	18·6% (8·64 to 30·1)	−1·16% (−9·59 to 7·11)
		Iceland	2460 (1790 to 3340)	1560 (1140 to 2110)	4100 (2980 to 5530)	1540 (1120 to 2060)	66·4% (50·5 to 82·0)	−1·50% (−10·5 to 7·14)
		Ireland	34 700 (25 600 to 46 100)	1650 (1220 to 2190)	58 600 (43 500 to 78 600)	1630 (1210 to 2190)	69·0% (54·6 to 83·8)	−1·45% (−9·62 to 6·64)
		Israel	48 500 (35 900 to 64 100)	1670 (1240 to 2220)	87 900 (65 700 to 118 000)	1650 (1240 to 2220)	81·5% (67·1 to 97·8)	−1·33% (−8·92 to 7·17)
		Italy	1 540 000 (1 280 000 to 1 880 000)	3390 (2830 to 4140)	1 990 000 (1 660 000 to 2 410 000)	3380 (2820 to 4110)	29·0% (25·7 to 32·2)	−0·498% (−2·71 to 1·94)
		Luxembourg	4430 (3290 to 5980)	1630 (1220 to 2210)	7520 (5560 to 10 100)	1620 (1200 to 2180)	69·5% (55·4 to 84·7)	−0·976% (−9·14 to 7·86)
		Malta	5380 (4310 to 6290)	2210 (1770 to 2580)	9820 (7850 to 11 600)	2190 (1750 to 2580)	82·6% (68·9 to 98·1)	−0·988% (−7·94 to 6·48)
		Monaco	571 (424 to 757)	1650 (1230 to 2200)	713 (520 to 945)	1630 (1200 to 2190)	24·7% (14·9 to 34·3)	−1·09% (−8·56 to 6·28)
		Netherlands	167 000 (122 000 to 225 000)	1660 (1220 to 2230)	268 000 (197 000 to 352 000)	1640 (1210 to 2150)	60·0% (46·8 to 73·5)	−0·890% (−8·44 to 7·55)
		Norway	165 000 (132 000 to 201 000)	5380 (4310 to 6600)	246 000 (197 000 to 302 000)	5380 (4300 to 6620)	49·2% (46·0 to 52·0)	−0·157% (−1·79 to 1·45)
		Portugal	124 000 (89 800 to 164 000)	1650 (1210 to 2170)	168 000 (125 000 to 221 000)	1630 (1220 to 2160)	34·8% (24·0 to 47·3)	−1·00% (−8·49 to 7·28)
		San Marino	329 (239 to 432)	1650 (1210 to 2170)	483 (355 to 639)	1630 (1200 to 2200)	46·8% (34·4 to 58·4)	−0·898% (−8·87 to 6·72)
		Spain	495 000 (364 000 to 662 000)	1650 (1220 to 2220)	671 000 (497 000 to 894 000)	1630 (1210 to 2170)	35·6% (25·4 to 48·1)	−1·07% (−8·83 to 7·76)
		Sweden	139 000 (104 000 to 184 000)	1970 (1470 to 2610)	193 000 (142 000 to 258 000)	1970 (1450 to 2640)	39·5% (28·5 to 51·5)	0·227% (−7·75 to 8·28)
		Switzerland	247 000 (223 000 to 273 000)	4890 (4410 to 5390)	382 000 (346 000 to 419 000)	4840 (4380 to 5320)	54·5% (45·0 to 65·0)	−1·07% (−7·19 to 5·64)
		UK	1 030 000 (866 000 to 1 230 000)	2420 (2040 to 2890)	1 390 000 (1 170 000 to 1 660 000)	2380 (2010 to 2860)	34·9% (32·2 to 37·3)	−1·52% (−3·35 to 0·161)
**Latin America and Caribbean**			**4 040 000 (3 210 000 to 5 030 000)**	**2960 (2360 to 3670)**	**7 830 000 (6 270 000 to 9 740 000)**	**2990 (2390 to 3710)**	**93·9% (90·9 to 96·9)**	**0·854% (−0·686 to 2·32)**
	Andean Latin America		501 000 (382 000 to 647 000)	3710 (2840 to 4750)	943 000 (706 000 to 1 210 000)	3610 (2700 to 4610)	88·1% (78·6 to 98·3)	−2·81% (−7·83 to 2·48)
		Bolivia	71 200 (52 900 to 93 800)	3440 (2560 to 4510)	136 000 (101 000 to 180 000)	3350 (2480 to 4390)	91·5% (77·2 to 109)	−2·76% (−10·0 to 5·93)
		Ecuador	170 000 (125 000 to 212 000)	4590 (3390 to 5710)	315 000 (231 000 to 398 000)	4430 (3270 to 5580)	85·8% (72·7 to 102)	−3·45% (−10·2 to 4·54)
		Peru	261 000 (193 000 to 342 000)	3360 (2500 to 4390)	492 000 (363 000 to 639 000)	3290 (2410 to 4270)	88·7% (75·2 to 105)	−2·24% (−9·36 to 6·57)
	Caribbean		433 000 (323 000 to 573 000)	2820 (2100 to 3710)	671 000 (500 000 to 891 000)	2780 (2080 to 3690)	55·1% (48·7 to 61·2)	−1·21% (−5·33 to 2·62)
		Antigua and Barbuda	734 (546 to 972)	2890 (2150 to 3860)	1350 (986 to 1810)	2840 (2110 to 3760)	83·9% (67·8 to 101)	−1·58% (−9·19 to 6·54)
		The Bahamas	2410 (1790 to 3180)	2880 (2140 to 3800)	4710 (3480 to 6260)	2830 (2100 to 3740)	95·9% (81·3 to 112)	−1·75% (−9·11 to 6·24)
		Barbados	3890 (2900 to 5110)	2920 (2180 to 3870)	6640 (4860 to 8850)	2880 (2120 to 3810)	70·6% (56·7 to 88·9)	−1·67% (−9·25 to 7·70)
		Belize	1680 (1250 to 2230)	2900 (2150 to 3800)	3730 (2750 to 4930)	2880 (2120 to 3800)	121% (103 to 141)	−0·663% (−8·46 to 7·72)
		Bermuda	924 (683 to 1220)	2800 (2070 to 3640)	1640 (1210 to 2160)	2760 (2030 to 3640)	77·3% (62·2 to 93·9)	−1·46% (−9·18 to 7·08)
		Cuba	166 000 (124 000 to 222 000)	2760 (2050 to 3700)	242 000 (181 000 to 322 000)	2710 (2030 to 3600)	46·3% (35·0 to 58·1)	−1·56% (−9·27 to 6·45)
		Dominica	953 (700 to 1270)	2920 (2150 to 3910)	1280 (944 to 1680)	2870 (2110 to 3750)	34·6% (23·7 to 46·4)	−1·84% (−8·55 to 6·03)
		Dominican Republic	68 800 (51 000 to 90 500)	2740 (2020 to 3580)	116 000 (86 200 to 154 000)	2720 (2020 to 3590)	68·7% (56·4 to 82·7)	−0·562% (−7·99 to 7·60)
		Grenada	865 (644 to 1140)	2920 (2170 to 3880)	1530 (1140 to 2020)	2910 (2160 to 3800)	77·0% (63·4 to 92·5)	−0·435% (−7·19 to 7·38)
		Guyana	5360 (3990 to 7070)	2990 (2220 to 3920)	8020 (6010 to 10 700)	2920 (2190 to 3850)	49·8% (37·0 to 60·0)	−2·17% (−10·4 to 4·33)
		Haiti	53 300 (39 400 to 71 800)	2850 (2120 to 3770)	83 400 (61 300 to 112 000)	2800 (2070 to 3710)	56·5% (45·0 to 68·8)	−1·72% (−9·00 to 5·44)
		Jamaica	29 000 (21 400 to 37 900)	2940 (2170 to 3840)	41 300 (30 700 to 54 400)	2940 (2180 to 3870)	42·2% (31·8 to 55·5)	0·00 491% (−7·46 to 8·99)
		Puerto Rico	61 800 (45 500 to 81 300)	2950 (2170 to 3890)	95 000 (70 000 to 125 000)	2920 (2170 to 3870)	53·8% (41·7 to 65·9)	−1·02% (−8·95 to 6·39)
		Saint Kitts and Nevis	480 (356 to 630)	2890 (2140 to 3820)	881 (643 to 1210)	2860 (2110 to 3840)	83·5% (59·6 to 107)	−1·30% (−9·86 to 5·94)
		Saint Lucia	1530 (1130 to 2040)	2930 (2180 to 3890)	2840 (2100 to 3740)	2840 (2110 to 3720)	85·8% (71·0 to 106)	−3·02% (−9·94 to 7·16)
		Saint Vincent and the Grenadines	1120 (826 to 1470)	3000 (2210 to 3950)	2080 (1550 to 2740)	2970 (2220 to 3880)	86·6% (70·1 to 104)	−1·15% (−9·41 to 7·27)
		Suriname	4280 (3130 to 5730)	2860 (2110 to 3780)	7630 (5630 to 10 100)	2880 (2130 to 3780)	78·3% (65·8 to 92·6)	0·853% (−5·85 to 8·60)
		Trinidad and Tobago	13 700 (10 100 to 17 900)	2800 (2070 to 3640)	25 200 (18 400 to 33 500)	2760 (2030 to 3640)	84·2% (70·0 to 101)	−1·46% (−9·18 to 7·08)
		Virgin Islands	1500 (1110 to 2030)	2870 (2130 to 3820)	2570 (1910 to 3410)	2840 (2120 to 3740)	71·2% (53·6 to 89·4)	−0·988% (−8·59 to 7·04)
	Central Latin America		2 170 000 (1 750 000 to 2 660 000)	4100 (3300 to 5010)	4 360 000 (3 510 000 to 5 370 000)	4140 (3340 to 5090)	101% (96·5 to 105)	1·12% (−1·15 to 3·37)
		Colombia	433 000 (321 000 to 563 000)	3690 (2750 to 4780)	872 000 (654 000 to 1 130 000)	3700 (2780 to 4810)	102% (86·2 to 119)	0·347% (−7·33 to 8·69)
		Costa Rica	43 400 (32 700 to 56 300)	3730 (2810 to 4820)	86 700 (65 200 to 113 000)	3720 (2800 to 4840)	99·8% (83·4 to 115)	−0·232% (−8·26 to 7·29)
		El Salvador	63 500 (47 600 to 81 800)	3750 (2810 to 4830)	94 500 (71 400 to 122 000)	3810 (2870 to 4920)	48·9% (39·1 to 59·6)	1·61% (−5·36 to 9·32)
		Guatemala	95 700 (71 600 to 125 000)	3800 (2840 to 4880)	188 000 (141 000 to 244 000)	3890 (2910 to 5020)	96·8% (81·8 to 113)	2·60% (−5·02 to 10·8)
		Honduras	49 500 (37 100 to 64 400)	3740 (2800 to 4860)	102 000 (76 400 to 134 000)	3790 (2830 to 4920)	107% (91·0 to 124)	1·15% (−6·67 to 9·85)
		Mexico	1 190 000 (1 010 000 to 1 410 000)	4460 (3790 to 5270)	2 370 000 (2 010 000 to 2 810 000)	4530 (3840 to 5350)	99·2% (96·1 to 103)	1·59% (0·0138 to 3·22)
		Nicaragua	36 500 (27 300 to 47 100)	3720 (2780 to 4790)	69 800 (52 100 to 92 000)	3780 (2830 to 4960)	91·1% (77·8 to 108)	1·64% (−5·51 to 10·5)
		Panama	36 800 (27 500 to 48 000)	3730 (2780 to 4850)	74 600 (55 600 to 96 600)	3770 (2810 to 4890)	103% (88·2 to 116)	1·20% (−5·81 to 8·02)
		Venezuela	222 000 (167 000 to 285 000)	3780 (2830 to 4820)	503 000 (376 000 to 657 000)	3810 (2860 to 4950)	126% (110 to 145)	0·913% (−6·53 to 9·82)
	Tropical Latin America		938 000 (775 000 to 1 150 000)	1710 (1410 to 2080)	1 860 000 (1 530 000 to 2 310 000)	1730 (1430 to 2130)	98·4% (93·5 to 104)	1·51% (−0·990 to 4·34)
		Brazil	912 000 (756 000 to 1 120 000)	1700 (1410 to 2070)	1 810 000 (1 490 000 to 2 240 000)	1730 (1430 to 2120)	98·6% (93·6 to 104)	1·50% (−1·05 to 4·33)
		Paraguay	26 000 (19 000 to 34 200)	1910 (1390 to 2510)	49 600 (36 100 to 65 900)	1960 (1430 to 2580)	91·2% (75·7 to 107)	2·58% (−5·64 to 11·4)
**North Africa and Middle East**			**1 080 000 (791 000 to 1 470 000)**	**977 (720 to 1320)**	**2 030 000 (1 510 000 to 2 750 000)**	**987 (732 to 1320)**	**88·6% (82·3 to 95·4)**	**0·967% (−1·80 to 3·93)**
	Afghanistan		39 300 (28 900 to 53 700)	987 (735 to 1330)	48 500 (36 000 to 65 600)	1010 (736 to 1360)	23·4% (9·73 to 38·8)	2·37% (−7·14 to 11·5)
	Algeria		76 500 (55 300 to 105 000)	969 (705 to 1320)	161 000 (119 000 to 222 000)	979 (721 to 1330)	111% (95·1 to 128)	1·03% (−6·51 to 8·59)
	Bahrain		1240 (914 to 1680)	1080 (789 to 1450)	6330 (4520 to 8750)	1070 (781 to 1460)	409% (354 to 456)	−0·622% (−9·23 to 6·22)
	Egypt		175 000 (127 000 to 241 000)	970 (710 to 1320)	324 000 (238 000 to 440 000)	985 (726 to 1320)	84·8% (71·3 to 102)	1·55% (−5·38 to 10·9)
	Iran		189 000 (138 000 to 259 000)	1010 (748 to 1390)	354 000 (263 000 to 482 000)	1030 (761 to 1400)	87·3% (74·9 to 102)	1·46% (−3·35 to 8·05)
	Iraq		49 300 (36 100 to 66 800)	1020 (745 to 1390)	106 000 (78 700 to 145 000)	1040 (769 to 1410)	116% (101 to 136)	1·51% (−5·35 to 10·7)
	Jordan		10 800 (7830 to 14 500)	1160 (851 to 1570)	36 400 (26 600 to 48 500)	1150 (844 to 1540)	237% (210 to 265)	−1·01% (−8·68 to 7·36)
	Kuwait		5040 (3740 to 6820)	1010 (739 to 1360)	13 300 (9890 to 18 100)	993 (739 to 1330)	164% (138 to 190)	−1·16% (−9·21 to 6·74)
	Lebanon		15 900 (11 500 to 21 900)	967 (705 to 1310)	22 400 (16 500 to 30 600)	959 (706 to 1310)	40·6% (27·4 to 53·3)	−0·835% (−9·43 to 7·44)
	Libya		11 700 (8640 to 15 800)	976 (720 to 1310)	22 700 (16 900 to 30 600)	972 (715 to 1300)	93·4% (75·3 to 112)	−0·374% (−9·03 to 8·01)
	Morocco		81 900 (60 500 to 111 000)	969 (720 to 1310)	147 000 (107 000 to 197 000)	979 (722 to 1310)	79·3% (64·7 to 96·6)	1·09% (−6·17 to 10·0)
	Oman		4200 (3060 to 5840)	1010 (736 to 1370)	8890 (6590 to 12 400)	1030 (760 to 1400)	112% (94·2 to 131)	2·80% (−4·53 to 11·5)
	Palestine		4840 (3540 to 6580)	976 (721 to 1320)	10 500 (7700 to 14 300)	996 (733 to 1340)	116% (97·1 to 136)	2·04% (−5·75 to 11·2)
	Qatar		1200 (864 to 1680)	1090 (804 to 1500)	7200 (5090 to 10 200)	1070 (787 to 1450)	500% (447 to 559)	−1·64% (−8·76 to 7·96)
	Saudi Arabia		41 600 (30 700 to 56 900)	972 (713 to 1330)	87 400 (63 900 to 119 000)	968 (710 to 1300)	110% (94·3 to 128)	−0·445% (−6·92 to 7·74)
	Sudan		57 000 (42 000 to 77 300)	951 (701 to 1280)	89 100 (65 800 to 122 000)	958 (703 to 1300)	56·3% (43·1 to 69·7)	0·725% (−7·60 to 8·89)
	Syria		32 500 (23 900 to 44 400)	949 (695 to 1280)	58 900 (42 700 to 81 500)	949 (694 to 1290)	81·0% (67·1 to 96·4)	0·0210% (−7·18 to 8·70)
	Tunisia		33 100 (24 000 to 46 200)	967 (705 to 1340)	59 800 (43 600 to 81 400)	974 (713 to 1310)	80·4% (64·4 to 99·9)	0·708% (−7·72 to 10·2)
	Türkiye		213 000 (154 000 to 289 000)	957 (695 to 1290)	387 000 (286 000 to 526 000)	950 (709 to 1280)	81·9% (66·3 to 99·0)	−0·707% (−8·65 to 8·42)
	United Arab Emirates		3850 (2770 to 5310)	1020 (746 to 1370)	22 900 (15 900 to 32 000)	1010 (735 to 1400)	494% (444 to 545)	−0·0445% (−7·34 to 8·28)
	Yemen		29 600 (21 500 to 41 200)	948 (693 to 1300)	56 500 (41 300 to 76 600)	949 (702 to 1280)	90·7% (75·6 to 107)	0·00 874% (−7·36 to 8·07)
**South Asia**			**11 200 000 (8 470 000 to 14 600 000)**	**3260 (2480 to 4200)**	**21 400 000 (16 200 000 to 27 800 000)**	**3270 (2480 to 4200)**	**91·4% (87·4 to 96·3)**	**0·333% (−1·09 to 1·95)**
	Bangladesh		686 000 (516 000 to 909 000)	2320 (1740 to 3060)	1 570 000 (1 170 000 to 2 080 000)	2340 (1740 to 3080)	129% (110 to 147)	0·685% (−7·38 to 7·80)
	Bhutan		3690 (2750 to 4870)	2310 (1740 to 3050)	6540 (4930 to 8670)	2380 (1790 to 3160)	77·4% (63·9 to 91·7)	2·96% (−4·55 to 11·1)
	India		9 550 000 (7 250 000 to 12 500 000)	3510 (2670 to 4510)	18 200 000 (13 900 000 to 23 700 000)	3480 (2640 to 4470)	90·9% (86·4 to 96·1)	−0·782% (−2·24 to 0·874)
	Nepal		120 000 (98 300 to 146 000)	2000 (1650 to 2430)	218 000 (178 000 to 267 000)	2050 (1700 to 2510)	81·9% (68·7 to 95·6)	2·85% (−4·21 to 10·4)
	Pakistan		800 000 (595 000 to 1 070 000)	2470 (1850 to 3290)	1 340 000 (997 000 to 1 780 000)	2600 (1950 to 3430)	67·6% (58·6 to 77·0)	5·08% (−0·0329 to 10·5)
**Southeast Asia, east Asia, and Oceania**			**15 900 000 (12 100 000 to 20 300 000)**	**2490 (1930 to 3160)**	**30 700 000 (23 600 000 to 39 000 000)**	**2530 (1970 to 3200)**	**93·8% (90·7 to 97·3)**	**1·75% (0·316 to 2·95)**
	East Asia		10 800 000 (8 180 000 to 14 000 000)	2240 (1720 to 2850)	21 400 000 (16 300 000 to 27 500 000)	2270 (1760 to 2900)	97·5% (93·3 to 102)	1·53% (−0·0784 to 2·91)
		China	10 200 000 (7 720 000 to 13 300 000)	2180 (1680 to 2790)	20 300 000 (15 500 000 to 26 200 000)	2220 (1710 to 2840)	99·0% (94·5 to 104)	1·69% (0·0412 to 3·18)
		North Korea	187 000 (141 000 to 248 000)	2990 (2260 to 3870)	339 000 (255 000 to 444 000)	3040 (2300 to 3970)	81·0% (64·6 to 97·0)	1·80% (−5·90 to 9·16)
		Taiwan (province of China)	424 000 (332 000 to 527 000)	3860 (3070 to 4790)	718 000 (570 000 to 895 000)	3890 (3100 to 4830)	69·5% (56·0 to 81·3)	0·779% (−5·72 to 6·99)
		Oceania	51 400 (38 900 to 68 300)	3190 (2430 to 4140)	97 200 (73 000 to 127 000)	3280 (2480 to 4240)	89·0% (79·7 to 100)	3·11% (−1·98 to 7·83)
		American Samoa	410 (310 to 546)	3110 (2360 to 4120)	676 (515 to 880)	3210 (2460 to 4180)	64·8% (51·3 to 75·8)	3·02% (−4·97 to 9·76)
		Cook Islands	260 (195 to 345)	3250 (2460 to 4260)	417 (313 to 546)	3370 (2540 to 4420)	60·1% (46·7 to 71·3)	3·77% (−3·85 to 10·1)
		Federated States of Micronesia	648 (485 to 842)	3220 (2420 to 4150)	893 (669 to 1190)	3340 (2520 to 4270)	37·8% (24·7 to 51·9)	3·93% (−2·93 to 10·8)
		Fiji	6400 (4870 to 8350)	3720 (2860 to 4760)	11 400 (8510 to 15 100)	3750 (2830 to 4820)	78·9% (66·2 to 92·0)	0·613% (−6·41 to 7·86)
		Guam	1480 (1110 to 1930)	3050 (2300 to 3960)	2770 (2080 to 3640)	3160 (2380 to 4120)	87·4% (75·3 to 102)	3·50% (−2·71 to 10·7)
		Kiribati	509 (378 to 668)	3470 (2630 to 4520)	805 (603 to 1080)	3560 (2680 to 4590)	58·2% (45·0 to 72·4)	2·61% (−4·03 to 8·94)
		Marshall Islands	232 (176 to 309)	3110 (2360 to 4120)	463 (346 to 609)	3230 (2430 to 4120)	99·7% (83·8 to 115)	3·62% (−3·48 to 9·85)
		Nauru	42·1 (31·7 to 56·3)	3190 (2400 to 4110)	39·7 (29·6 to 53·9)	3330 (2500 to 4320)	−5·69% (−13·1 to 2·22)	4·37% (−2·32 to 11·4)
		Niue	32·0 (23·9 to 41·9)	3250 (2450 to 4210)	32·0 (24·0 to 41·7)	3380 (2540 to 4380)	−0·203% (−7·60 to 7·87)	4·07% (−2·89 to 11·1)
		Northern Mariana Islands	306 (235 to 404)	3090 (2330 to 4010)	749 (559 to 1000)	3180 (2400 to 4130)	145% (124 to 167)	2·95% (−5·05 to 9·90)
		Palau	189 (143 to 251)	3250 (2470 to 4260)	307 (229 to 410)	3370 (2510 to 4340)	62·8% (49·9 to 77·6)	3·74% (−2·79 to 12·6)
		Papua New Guinea	31 700 (23 900 to 42 300)	3110 (2360 to 4120)	64 000 (48 200 to 84 100)	3220 (2440 to 4160)	102% (87·3 to 119)	3·50% (−4·34 to 10·0)
		Samoa	1470 (1100 to 1940)	3150 (2370 to 4100)	2030 (1540 to 2650)	3240 (2450 to 4200)	38·2% (27·0 to 49·7)	2·98% (−4·15 to 9·56)
		Solomon Islands	2710 (2000 to 3600)	3140 (2350 to 4090)	4000 (3020 to 5260)	3300 (2500 to 4270)	47·5% (36·5 to 61·9)	5·35% (−1·68 to 15·1)
		Tokelau	17·2 (13·0 to 22·3)	3170 (2380 to 4080)	21·6 (16·1 to 28·2)	3320 (2500 to 4300)	25·2% (15·4 to 35·4)	4·61% (−2·67 to 12·5)
		Tonga	966 (719 to 1270)	3240 (2430 to 4210)	1170 (878 to 1510)	3380 (2540 to 4330)	20·7% (13·0 to 28·2)	4·47% (−1·87 to 11·5)
		Tuvalu	104 (76·5 to 136)	3180 (2370 to 4130)	145 (109 to 190)	3300 (2500 to 4270)	40·2% (30·8 to 50·5)	3·87% (−3·16 to 10·8)
		Vanuatu	1160 (862 to 1520)	2980 (2240 to 3880)	2580 (1920 to 3410)	3150 (2370 to 4140)	123% (106 to 141)	5·53% (−1·73 to 13·6)
	Southeast Asia		4 970 000 (3 850 000 to 6 380 000)	3470 (2690 to 4420)	9 230 000 (7 120 000 to 11 900 000)	3520 (2700 to 4460)	85·8% (81·4 to 90·4)	1·42% (−0·527 to 3·26)
		Cambodia	71 300 (52 500 to 94 200)	3180 (2370 to 4140)	147 000 (110 000 to 195 000)	3260 (2460 to 4210)	106% (92·1 to 124)	2·59% (−4·04 to 10·5)
		Indonesia	2 010 000 (1 560 000 to 2 540 000)	3620 (2850 to 4520)	3 500 000 (2 720 000 to 4 450 000)	3700 (2890 to 4650)	74·5% (69·3 to 79·8)	2·22% (−0·833 to 4·97)
		Laos	36 200 (27 000 to 48 200)	3230 (2420 to 4230)	62 700 (47 700 to 83 400)	3310 (2510 to 4310)	73·0% (59·9 to 88·0)	2·60% (−4·67 to 10·3)
		Malaysia	187 000 (141 000 to 250 000)	3290 (2500 to 4330)	430 000 (321 000 to 567 000)	3350 (2510 to 4340)	130% (114 to 149)	1·75% (−4·48 to 9·24)
		Maldives	2290 (1690 to 3040)	3210 (2400 to 4180)	4620 (3490 to 6080)	3270 (2460 to 4220)	102% (85·0 to 121)	1·85% (−5·71 to 9·43)
		Mauritius	13 300 (10 100 to 17 600)	3370 (2550 to 4400)	29 700 (22 300 to 39 300)	3700 (2810 to 4790)	123% (105 to 141)	9·81% (1·51 to 18·1)
		Myanmar	386 000 (289 000 to 507 000)	3170 (2390 to 4110)	613 000 (462 000 to 808 000)	3270 (2450 to 4250)	58·8% (47·0 to 71·5)	3·21% (−2·97 to 12·0)
		Philippines	516 000 (390 000 to 692 000)	2980 (2240 to 3900)	983 000 (736 000 to 1 320 000)	3010 (2270 to 3950)	90·7% (86·8 to 94·8)	0·989% (−0·382 to 2·48)
		Seychelles	875 (655 to 1160)	3200 (2420 to 4200)	1630 (1230 to 2170)	3300 (2470 to 4310)	85·9% (69·6 to 105)	3·06% (−4·88 to 12·0)
		Sri Lanka	204 000 (153 000 to 272 000)	3230 (2420 to 4250)	387 000 (289 000 to 516 000)	3380 (2540 to 4450)	89·5% (75·7 to 105)	4·54% (−2·40 to 12·9)
		Thailand	691 000 (521 000 to 919 000)	3240 (2450 to 4260)	1 500 000 (1 130 000 to 1 960 000)	3220 (2430 to 4170)	117% (101 to 134)	−0·410% (−7·82 to 7·46)
		Timor-Leste	5960 (4410 to 8020)	3160 (2380 to 4120)	12 700 (9390 to 17 000)	3220 (2420 to 4280)	112% (95·6 to 132)	2·01% (−5·47 to 9·87)
		Viet Nam	842 000 (659 000 to 1 060 000)	4090 (3220 to 5130)	1 550 000 (1 210 000 to 1 960 000)	4220 (3310 to 5230)	83·9% (69·0 to 99·4)	3·07% (−4·06 to 9·19)
**Sub-Saharan Africa**			**1 420 000 (1 050 000 to 1 930 000)**	**1200 (888 to 1610)**	**2 310 000 (1 710 000 to 3 130 000)**	**1200 (889 to 1600)**	**62·4% (59·6 to 65·1)**	**−0·0283% (−1·25 to 1·21)**
	Central sub-Saharan Africa		126 000 (92 100 to 170 000)	1110 (812 to 1470)	227 000 (166 000 to 311 000)	1100 (812 to 1480)	79·9% (69·1 to 92·0)	−0·255% (−5·28 to 5·94)
		Angola	23 000 (16 800 to 31 200)	1090 (794 to 1450)	47 600 (34 800 to 64 500)	1100 (816 to 1480)	107% (89·5 to 129)	1·20% (−6·59 to 10·6)
		Central African Republic	5760 (4190 to 7940)	1100 (804 to 1490)	8670 (6220 to 12 000)	1100 (796 to 1480)	50·6% (36·5 to 65·9)	−0·119% (−7·98 to 7·99)
		Congo (Brazzaville)	5820 (4250 to 7950)	1120 (825 to 1520)	12 000 (8700 to 16 300)	1110 (807 to 1470)	107% (90·2 to 123)	−0·901% (−7·61 to 6·27)
		Democratic Republic of the Congo	87 300 (62 900 to 119 000)	1110 (809 to 1480)	151 000 (110 000 to 209 000)	1100 (797 to 1470)	73·2% (58·1 to 89·8)	−0·603% (−8·18 to 7·86)
		Equatorial Guinea	1000 (732 to 1370)	1120 (831 to 1510)	1960 (1440 to 2630)	1130 (835 to 1520)	95·0% (77·1 to 113)	0·830% (−7·78 to 10·7)
		Gabon	3030 (2200 to 4110)	1130 (823 to 1510)	5030 (3650 to 6990)	1130 (836 to 1530)	66·2% (52·2 to 79·8)	−0·368% (−7·46 to 7·45)
	Eastern sub-Saharan Africa		473 000 (349 000 to 643 000)	1170 (862 to 1560)	799 000 (587 000 to 1 080 000)	1160 (853 to 1540)	68·7% (64·1 to 73·7)	−0·552% (−2·98 to 2·05)
		Burundi	11 700 (8520 to 15 900)	1120 (813 to 1510)	23 200 (16 800 to 31 900)	1110 (811 to 1500)	98·7% (82·8 to 118)	−0·857% (−8·66 to 7·22)
		Comoros	1420 (1050 to 1900)	1120 (832 to 1490)	2280 (1680 to 3070)	1110 (819 to 1480)	61·2% (46·1 to 74·9)	−0·668% (−9·15 to 7·74)
		Djibouti	1110 (799 to 1530)	1120 (815 to 1510)	3180 (2320 to 4320)	1120 (816 to 1490)	187% (166 to 211)	−0·0184% (−7·56 to 8·21)
		Eritrea	4880 (3550 to 6750)	1120 (823 to 1490)	10 200 (7450 to 13 900)	1110 (819 to 1470)	108% (91·9 to 124)	−0·704% (−8·59 to 6·69)
		Ethiopia	144 000 (105 000 to 195 000)	1180 (864 to 1600)	224 000 (164 000 to 305 000)	1160 (851 to 1570)	55·6% (41·9 to 68·4)	−1·24% (−8·75 to 6·46)
		Kenya	64 600 (47 300 to 87 400)	1380 (1020 to 1840)	126 000 (93 000 to 171 000)	1370 (1010 to 1830)	95·2% (90·1 to 100)	−0·912% (−2·74 to 0·835)
		Madagascar	28 500 (21 000 to 38 600)	1120 (831 to 1500)	50 300 (36 600 to 69 600)	1110 (816 to 1490)	76·4% (61·0 to 95·0)	−0·798% (−8·49 to 8·94)
		Malawi	20 400 (15 000 to 27 700)	1120 (819 to 1510)	31 600 (23 000 to 43 000)	1110 (815 to 1490)	54·8% (42·5 to 69·7)	−0·853% (−8·67 to 8·78)
		Mozambique	32 600 (23 800 to 44 200)	1110 (827 to 1480)	47 400 (34 800 to 64 100)	1110 (807 to 1490)	45·5% (33·1 to 59·6)	−0·291% (−8·49 to 8·60)
		Rwanda	11 600 (8600 to 15 600)	1120 (832 to 1490)	25 000 (18 100 to 34 000)	1110 (810 to 1490)	116% (96·5 to 136)	−1·03% (−8·83 to 7·76)
		Somalia	13 800 (9830 to 18 900)	1120 (813 to 1510)	25 900 (18 500 to 35 900)	1120 (819 to 1510)	87·8% (75·2 to 102)	−0·191% (−6·76 to 6·82)
		South Sudan	14 800 (10 900 to 20 300)	1120 (821 to 1500)	20 200 (14 800 to 27 900)	1120 (818 to 1510)	36·4% (25·3 to 49·1)	0·117% (−7·60 to 9·06)
		Tanzania	68 700 (50 200 to 92 900)	1110 (822 to 1490)	118 000 (85 300 to 159 000)	1110 (800 to 1480)	71·2% (58·5 to 86·1)	−0·300% (−7·63 to 8·44)
		Uganda	36 900 (26 900 to 50 800)	1130 (832 to 1530)	60 300 (44 200 to 81 900)	1120 (819 to 1520)	63·4% (50·4 to 76·3)	−0·853% (−8·93 to 7·18)
		Zambia	17 900 (13 100 to 24 300)	1130 (831 to 1510)	30 600 (22 200 to 40 900)	1130 (824 to 1500)	71·0% (57·3 to 84·9)	−0·324% (−8·34 to 6·94)
	Southern sub-Saharan Africa		236 000 (175 000 to 318 000)	1730 (1280 to 2320)	386 000 (286 000 to 518 000)	1760 (1310 to 2310)	63·6% (59·4 to 68·3)	1·72% (−0·724 to 4·39)
		Botswana	6300 (4620 to 8450)	2160 (1590 to 2900)	11 200 (8230 to 15 000)	2170 (1600 to 2870)	78·6% (65·5 to 93·0)	0·736% (−6·35 to 8·57)
		Eswatini	2540 (1850 to 3480)	1740 (1280 to 2330)	3590 (2630 to 4890)	1780 (1300 to 2360)	41·4% (29·0 to 54·5)	2·29% (−5·26 to 11·4)
		Lesotho	7030 (5120 to 9580)	1670 (1220 to 2230)	7900 (5750 to 10 700)	1720 (1260 to 2300)	12·3% (4·17 to 21·3)	3·49% (−3·84 to 10·7)
		Namibia	6530 (4780 to 8750)	1660 (1220 to 2220)	8890 (6630 to 12 000)	1640 (1230 to 2210)	36·2% (24·7 to 48·3)	−1·25% (−9·10 to 6·90)
		South Africa	179 000 (133 000 to 243 000)	1740 (1290 to 2350)	312 000 (231 000 to 418 000)	1770 (1310 to 2330)	74·3% (69·0 to 80·8)	1·50% (−1·64 to 4·72)
		Zimbabwe	34 600 (25 400 to 47 400)	1640 (1210 to 2210)	42 400 (31 000 to 56 900)	1650 (1220 to 2200)	22·6% (12·9 to 33·1)	0·826% (−6·49 to 9·54)
	Western sub-Saharan Africa		589 000 (431 000 to 798 000)	1120 (818 to 1490)	900 000 (666 000 to 1 220 000)	1120 (817 to 1490)	53·0% (50·1 to 56·5)	−0·120% (−1·58 to 1·48)
		Benin	12 000 (8780 to 16 400)	1080 (799 to 1470)	22 200 (16 500 to 30 500)	1090 (806 to 1470)	84·6% (69·4 to 102)	0·512% (−6·93 to 9·05)
		Burkina Faso	25 900 (18 800 to 35 100)	1090 (794 to 1460)	40 500 (29 600 to 55 000)	1090 (792 to 1460)	56·4% (44·0 to 69·4)	0·187% (−7·07 to 7·79)
		Cameroon	29 900 (21 600 to 40 200)	1100 (805 to 1470)	56 900 (41 200 to 77 900)	1110 (804 to 1490)	90·4% (73·8 to 107)	0·791% (−7·80 to 8·75)
		Cape Verde	1210 (878 to 1630)	1090 (795 to 1470)	1840 (1350 to 2490)	1100 (790 to 1500)	52·0% (36·6 to 70·6)	0·601% (−7·38 to 8·42)
		Chad	17 100 (12 500 to 23 000)	1080 (790 to 1450)	30 400 (22 000 to 41 600)	1090 (800 to 1460)	77·8% (63·3 to 91·8)	0·792% (−7·31 to 8·33)
		Côte d'Ivoire	29 600 (21 300 to 41 100)	1100 (797 to 1500)	53 100 (39 100 to 71 700)	1100 (799 to 1480)	79·5% (66·5 to 95·8)	−0·169% (−6·75 to 8·71)
		The Gambia	2730 (1970 to 3710)	1080 (786 to 1440)	4590 (3390 to 6330)	1080 (797 to 1470)	67·9% (53·9 to 81·1)	0·0583% (−7·68 to 7·30)
		Ghana	42 000 (30 400 to 57 100)	1100 (805 to 1460)	70 700 (51 800 to 96 300)	1110 (812 to 1490)	68·3% (54·7 to 84·9)	1·22% (−6·76 to 10·2)
		Guinea	22 400 (16 300 to 30 100)	1090 (796 to 1460)	28 500 (20 900 to 38 800)	1090 (801 to 1460)	27·2% (16·3 to 38·3)	0·0506% (−7·86 to 7·55)
		Guinea-Bissau	2090 (1540 to 2820)	1100 (812 to 1480)	3120 (2280 to 4280)	1100 (815 to 1490)	49·4% (36·7 to 61·7)	−0·239% (−8·16 to 7·34)
		Liberia	6880 (5020 to 9250)	1090 (798 to 1470)	9950 (7250 to 13 400)	1090 (798 to 1470)	44·6% (31·4 to 57·8)	0·184% (−8·00 to 8·06)
		Mali	26 500 (19 300 to 36 100)	1090 (801 to 1460)	44 500 (32 400 to 60 400)	1090 (797 to 1460)	67·8% (52·9 to 84·0)	−0·129% (−8·26 to 8·99)
		Mauritania	6200 (4510 to 8350)	1100 (798 to 1480)	10 900 (7930 to 14 800)	1100 (802 to 1480)	76·4% (61·7 to 89·3)	−0·118% (−8·47 to 6·93)
		Niger	18 700 (13 800 to 25 300)	1080 (797 to 1450)	37 700 (27 500 to 51 500)	1090 (797 to 1470)	102% (85·6 to 121)	0·651% (−6·64 to 9·47)
		Nigeria	305 000 (224 000 to 413 000)	1140 (843 to 1520)	415 000 (308 000 to 566 000)	1140 (840 to 1520)	36·3% (33·3 to 40·4)	−0·338% (−2·01 to 1·42)
		São Tomé and Príncipe	363 (263 to 496)	1080 (791 to 1470)	488 (355 to 656)	1080 (790 to 1450)	34·4% (21·9 to 49·7)	−0·0927% (−8·35 to 9·25)
		Senegal	22 100 (16 000 to 29 800)	1090 (802 to 1460)	37 100 (26 800 to 50 000)	1100 (798 to 1460)	67·8% (52·2 to 83·0)	0·472% (−8·35 to 9·58)
		Sierra Leone	10 900 (7920 to 14 600)	1080 (786 to 1440)	17 800 (12 800 to 24 200)	1090 (791 to 1470)	63·2% (50·5 to 79·9)	0·697% (−6·61 to 9·88)
		Togo	7420 (5430 to 10 200)	1080 (797 to 1470)	14 800 (10 700 to 20 400)	1090 (799 to 1470)	98·8% (83·3 to 116)	0·658% (−6·44 to 8·55)

Data in parentheses are 95% uncertainty intervals (UIs). Data are presented to three significant figures. GBD=Global Burden of Diseases, Injuries and Risk Factors Study.

**Figure 2:**
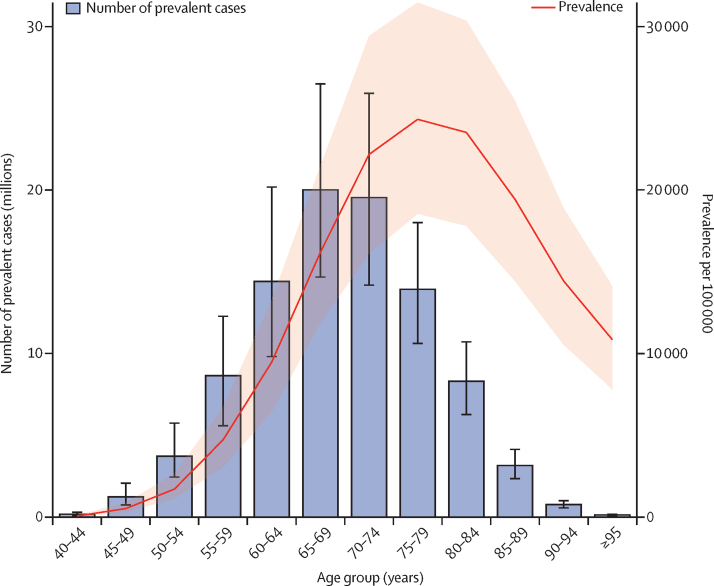
Global age-specific distribution of benign prostatic hyperplasia prevalence in 2019 Bars represent number of prevalent cases, whereas the red line represents age-specific prevalence per 100 000. The shaded area and error bars represent 95% uncertainty intervals.

Globally, men aged 65–74 years shared the greatest absolute burden of benign prostatic hyperplasia (figure 2), accounting for 42% of the total prevalent cases among men aged 40 years and older. The age-specific prevalence was highest in men aged 75–79 years, at 24 300 (95% UI 18 600–31 500) per 100 000, followed by the those aged 80–84 years, at 23 500 (17 800–30 400) per 100 000, and those aged 70–74 years, at 22 200 (16 100–29 400) per 100 000. Between 2000 and 2019, the number of prevalent cases of benign prostatic hyperplasia increased rapidly in all age groups (appendix 2 p 31). In men aged 40–44 years, the percentage increase was 22·6% (16·7–26·8). For men aged 80 years and older, the percentage increase was 173% (166–179).

**Figure 3:**
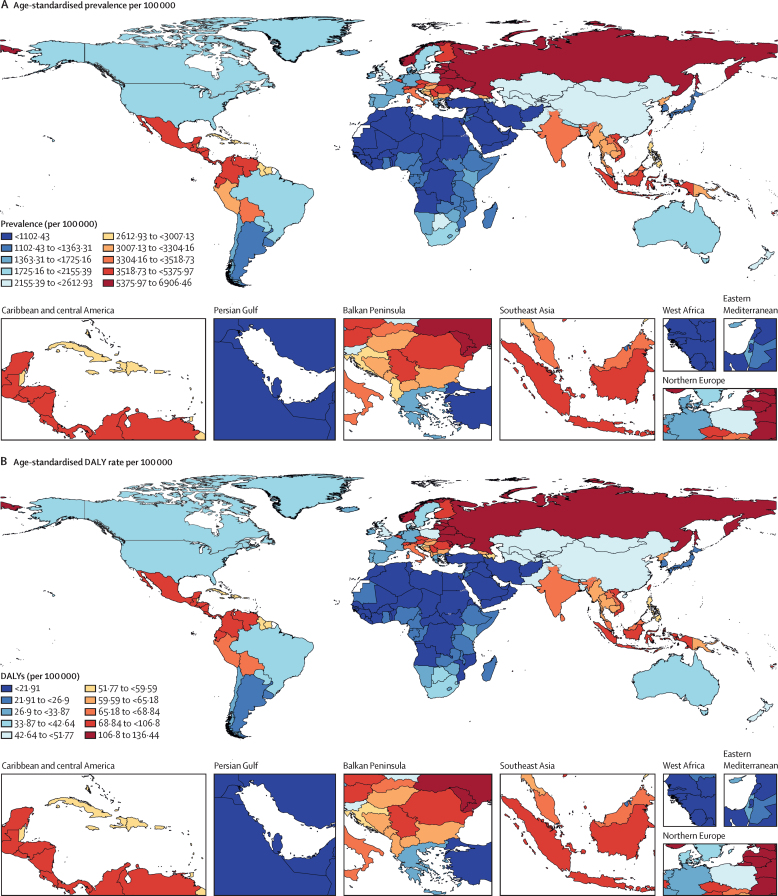
Global distribution of benign prostatic hyperplasia burden in 2019 (A) Age-standardised prevalence per 100 000. (B) Age-standardised rate of disability-adjusted life-years (DALYs) per 100 000.

There was substantial geographical variation in the prevalence of benign prostatic hyperplasia in 2019 (figure 3A). The highest age-standardised prevalence was observed in eastern Europe (6480 [95% UI 5130–8080] per 100 000), followed by central Latin America (4140 [3340–5090] per 100 000) and Andean Latin America (3610 [2700–4610] per 100 000). The lowest age-standardised prevalence was recorded in north Africa and the Middle East (987 [732–1320] per 100 000) and three sub-Saharan African regions: eastern sub-Saharan Africa (1160 [852–1540] per 100 000), western sub-Saharan Africa (1120 [817–1490] per 100 000), and central sub-Saharan Africa (1110 [812–1480] per 100 000). The age-standardised prevalence in five high-income regions (western Europe, high-income North America, high-income Asia Pacific, Australasia, and southern Latin America) ranged from 2250 (1800–2790) per 100 000 in western Europe to 1180 (906–1550) per 100 000 in the high-income Asia Pacific in 2019 (table).

**Figure 4:**
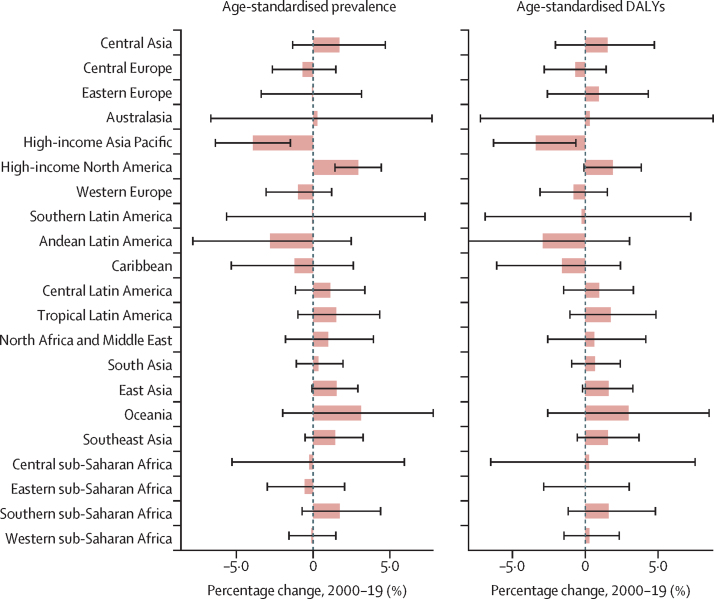
Percentage change in age-standardised prevalence of, and DALYs associated with, benign prostatic hyperplasia in 21 regions, 2000–19 Bars represent mean percentage change, and error bars represent 95% uncertainty intervals. DALY=disability-adjusted life-year.

Prevalent cases of benign prostatic hyperplasia increased in all 21 GBD regions between 2000 and 2019, with the percentage change ranging from 19·3% to 101%. The greatest increase was noted in central Latin America, with a 101% change (95% UI 96·5–105), followed by tropical Latin America (98·4% [93·5–104]), east Asia (97·5% [93·3–102]), and south Asia (91·4% [87·4–96·3]). The smallest increase was noted in three European regions (eastern, central, and western Europe). Despite this steady increase in the number of prevalent cases, 17 regions had a less than 2% change in age-standardised prevalence during the same period (figure 4). Of the remaining four regions, only two had change estimates that excluded zero in their uncertainty intervals: the high-income Asia Pacific, which saw a 3·92% (1·48–6·37) decrease, and high-income North America, which saw a 2·93% (1·42–4·44) increase (table).

The global distribution of the benign prostatic hyperplasia burden, as represented by age-standardised DALY rates, was the same as that for prevalence (figure 3A, B). In 2019, benign prostatic hyperplasia was responsible for 1·86 million (95% UI 1·13–2·78) DALYs globally, equating to an age-standardised DALY rate of 48·9 (29·7–72·6) per 100 000. Consistent with the findings for prevalence, the majority of the disease burden was concentrated in countries in eastern Europe, central Latin America, Andean Latin America, and southeast Asia. In 2019, the age-standardised DALY rate was 128 (76·5–190) per 100 000 in eastern Europe, 81·8 (50·2–121) per 100 000 in central Latin America, 71·9 (43·1–109) per 100 000 in Andean Latin America, and 69·7 (41·8–104) per 100 000 in southeast Asia (appendix pp 32–48). The temporal variation of DALYs was the same as that for prevalence.

At the national level, the age-standardised prevalence ranged from 949 per 100 000 to 6910 per 100 000 across countries and territories in 2019 (table). The highest age-standardised prevalences of benign prostatic hyperplasia were observed in Lithuania (6910 [95% UI 5830 to 7940] per 100 000), Russia (6510 [5110 to 8130] per 100 000), and Ukraine (6450 [5030 to 8050] per 100 000), whereas the lowest age-standardised prevalences were observed in Yemen (949 [702 to 1280] per 100 000), Syria (949 [694 to 1290] per 100 000), Sudan (958 [703 to 1300] per 100 000), and Lebanon (959 [706 to 1310] per 100 000). As noted for many regional estimates above, the percentage change in the age-standardised prevalence of individual countries during the 2000–19 period was small and often non-significant; the highest percentage increases in age-standardised prevalence were noted in Mauritius (9·81% [1·51 to 18·1]), followed by Vanuatu (5·53% [–1·73 to 13·6]), and the Solomon Islands (5·35% [–1·68 to 15·1]), whereas the greatest decreases were recorded in Brunei (4·00% [–5·61 to 12·4]), Belgium (3·91% [–6·96 to 20·1]), Ecuador (3·45% [–4·54 to 10·2]), and Singapore (3·18% [–4·46 to 11·1]; table).

**Figure 5:**
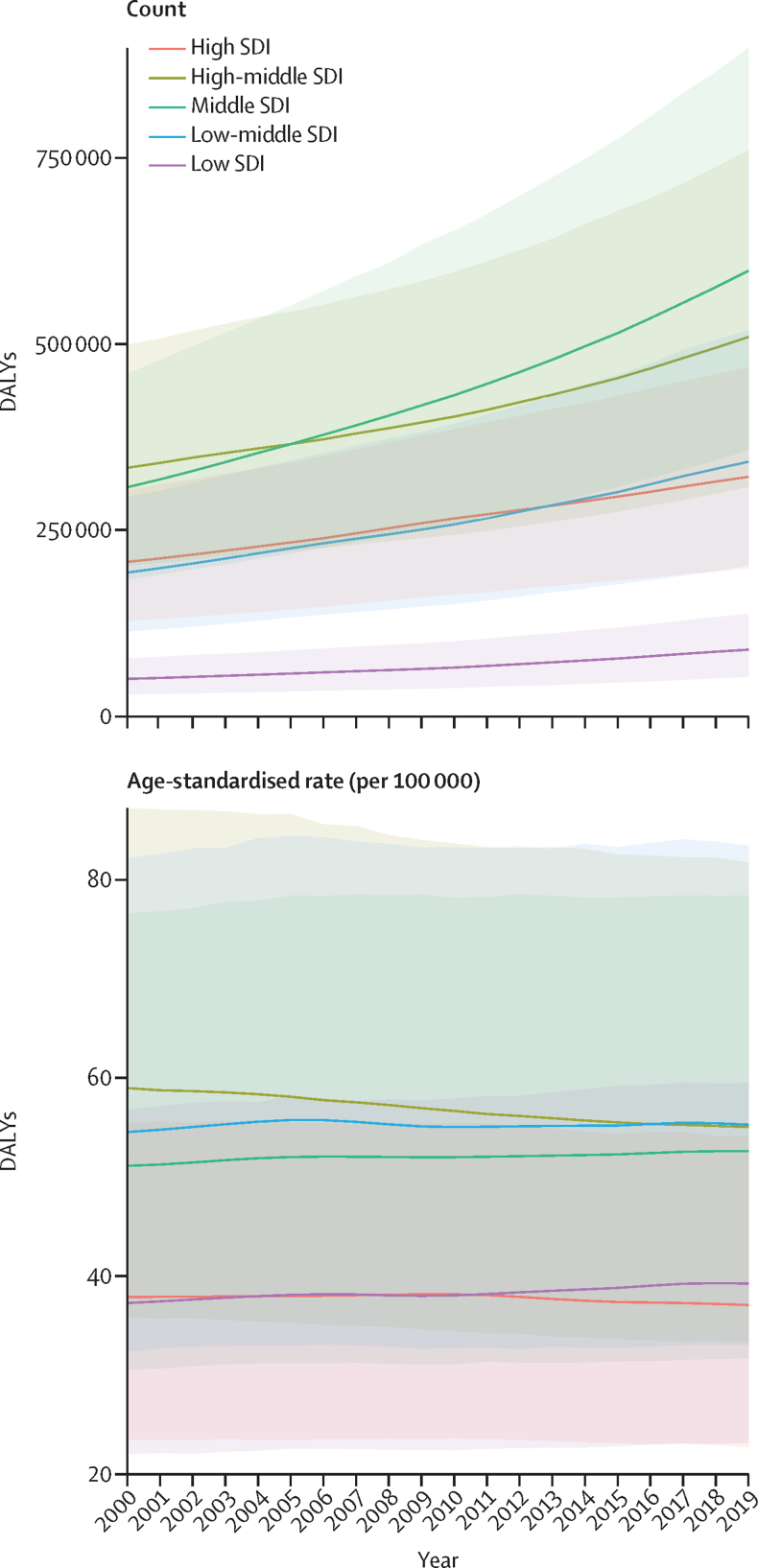
Temporal trend of count and age-standardised rate of DALYs by SDI quintile, 2000–19 The top panel illustrates changes in absolute DALY counts, and the bottom panel illustrates changes in age-standardised DALY rates. Countries were assigned to SDI quintiles on the basis of their SDI in the year 2019. The shaded areas represent 95% uncertainty intervals. DALY=disability-adjusted life-year. SDI=Socio-demographic Index.

We also assessed the disease burden in five SDI quintiles (figure 5). Between 2000 and 2019, the majority of the absolute DALY burden of benign prostatic hyperplasia was concentrated in the high-middle and middle SDI quintiles, with the fewest DALYs in the low SDI quintile. All five SDI quintiles observed an increase in the absolute DALY burden between 2000 and 2019. The most rapid increases were seen in the middle SDI quintile (94·7% [95% UI 91·8–97·6]), the low-middle SDI quintile (77·3% [74·1–81·2]), and the low SDI quintile (77·7% [72·9–83·2]). The high SDI quintile saw a 55·2% (52·6–58·2) increase and the high-middle SDI quintile saw a 52·8% (49·4–56·3) increase. During the same period, the high and high-middle SDI quintiles saw a small decrease in the age-standardised DALY rate, whereas the middle, low-middle, and low SDI quintiles saw small increases. The greatest increase in the age-standardised DALY rate was seen in the low SDI quintile (5·28% [2·41–8·36]). In contrast to the patterns we observed in the absolute DALY burden, the low-middle SDI quintile had the highest age-standardised DALY rate in 2019, surpassing that of the high-middle SDI quintile in 2016.

## Discussion

We present a comprehensive assessment of the temporal and geographical patterns of the benign prostatic hyperplasia burden from GBD 2019. Our findings are consistent with previous reports, showing that the absolute disease burden is rising in many parts of the world. The global number of prevalent cases almost doubled in the past 20 years. Despite the increase in the absolute benign prostatic hyperplasia burden, the global age-standardised prevalence and DALY rates remained largely unchanged during the study period, suggesting that population growth and ageing have a greater impact on driving the increased prevalence of, and DALYs associated with, benign prostatic hyperplasia at the global level than other risk factors for benign prostatic hyperplasia do.

Our study shows that the peak absolute benign prostatic hyperplasia burden occurred in men aged 65–69 years and the age-specific prevalence was highest in men aged 75–79 years. This trend contrasts with the age trend found in autopsy studies, where the histological prevalence continues to rise with advancing age,^4^, ^63^, ^64^, ^65^ but was similar to the age trend found in community-based studies, where the diagnosis of benign prostatic hyperplasia was made on the basis of lower urinary tract symptoms and prostatic enlargement in clinical practice.^23^, ^53^, ^66^, ^67^ Geographically, the age-standardised prevalence and DALY rates were lowest in countries in north Africa and the Middle East and sub-Saharan Africa, and highest in countries in eastern Europe. Although this geographical variation could be attributable to the varying stages at which each country is undergoing demographic and epidemiological transitions, it could also partially be explained by differences in the underlying risk factors in these populations.

Our study suggests a close and nuanced link between the benign prostatic hyperplasia burden and national sociodemographic status, and the potential for intervention to make an impact. As noted, the absolute benign prostatic hyperplasia burden has increased globally between 2000 and 2019, but age-standardised prevalence and DALY rates were more stable. This pattern of rising absolute burden with stability or only small changes in age-standardised rates was seen in most regions and many countries, reflecting the major role of widespread population growth and ageing in the substantial increase in benign prostatic hyperplasia cases. A rising absolute burden of benign prostatic hyperplasia was seen across all SDI quintiles, and the middle SDI quintile in particular carried the greatest absolute DALY burden by 2019. Notably, however, countries in the lowest three SDI quintiles (low, low-middle, and middle) had the largest percentage change in absolute DALYs between 2000 and 2019, and also had age-standardised DALY rates that overall trended upwards over the study period. Countries in the highest two SDI quintiles (high-middle and high) had somewhat smaller relative increases in absolute DALY counts and had age-standardised rates that overall trended downwards. Although population growth and ageing are the two most important factors contributing to the rising burden of benign prostatic hyperplasia worldwide, divergent trends in age-standardised rates suggest some influence from other risk factors for benign prostatic hyperplasia, such as metabolic syndrome, obesity, diabetes, and acute and chronic prostatic inflammation.^5^, ^9^, ^10^, ^11^, ^12^, ^13^, ^14^, ^15^ Rising age-standardised DALY rates in the bottom three SDI quintiles could reflect increased detection and diagnosis, or a true increase in disease frequency driven by rising levels of upstream risk factors. Downward trends in age-standardised DALY rates could reflect increased treatment initiation, advancement in surgical care and access, or improved control of upstream risk factors. Although the age-standardised prevalence and DALY rates declined in many high-income countries during the study period, we saw increasing age-standardised rates in the USA. This finding was consistent with the rising prevalence of major comorbidities associated with benign prostatic hyperplasia, such as diabetes, hypertension, cardiac disease, and hyperlipidaemia in the USA compared with other high-income countries.^47^, ^68^ This observation indicates that even high-income countries with similar advancement in economic development and similar age structures could have varying levels of benign prostatic hyperplasia depending on the prevalence of the underlying causes of benign prostatic hyperplasia in the population. This finding emphasises the broader relevance of benign prostatic hyperplasia to other non-communicable diseases and their control measures and serves as an urgent call for countries to strengthen efforts to address these public health challenges together.

With medical, social, and economic advances, people are living longer worldwide. Many countries are undergoing rapid changes in their population size, as well as the proportion of older people in their population. Consequently, addressing the burden of ageing-related diseases, such as benign prostatic hyperplasia, has to become one of the top global health priorities. In addition to imposing a substantial health burden, as shown in the present analysis, benign prostatic hyperplasia imposes substantial economic costs on societies. An analysis of the National Health and Nutrition Examination Survey-III done in the USA revealed that there were close to 8 million clinic visits for a primary or secondary diagnosis of benign prostatic hyperplasia in 2000, resulting in a direct cost of US$1·1 billion for treatment, excluding outpatient medication costs.^64^ The estimated economic burden of benign prostatic hyperplasia in the global population of men older than 65 years was $73·8 billion per year.^25^ Thus, our study findings have important implications for health service structure, human resource capacity building, and economic burden prediction. Although well documented, evidence-based prevention of benign prostatic hyperplasia is limited, disability and complications related to benign prostatic hyperplasia can be mitigated. In particular, there are several medical and surgical therapy options to reduce disability due to benign prostatic hyperplasia. Medical therapy involves the use of alpha blockers, 5-alpha reductase inhibitors, phosphodiesterase-5 inhibitors, anticholinergic agents, beta-3-agonists, or therapy with a combination of the above.^69^ Surgical therapy can be used for selected patients, such as those with renal insufficiency associated with benign prostatic hyperplasia, refractory urinary retention, recurrent urinary tract infection, recurrent bladder stone, gross haematuria, or failed medical therapy.^70^, ^71^ With the rising number of benign prostatic hyperplasia cases, the demand for diagnostic tools, medications, and hospital care will increase enormously. Therefore, the health structure and human resource capacity-building of a nation should be organised to meet these increasing demands. Despite its burden and increasing trends, efforts from the global community to design prevention strategies for benign prostatic hyperplasia are inadequate. Global, regional, and national efforts should begin and be integrated into broader non-communicable disease control efforts to prevent health loss due to benign prostatic hyperplasia.

This study has several limitations, which can be broadly organised into limitations of prevalence inputs, limitations of severity inputs, and analytical considerations. First, data to estimate the prevalence of benign prostatic hyperplasia are sparse and heterogeneous, and theycarry with them the inherent biases of administrative records from medical facilities and claims. With regard to scarcity, despite the international administrative data we used in our analyses, we did not have prevalence data for many countries, especially in sub-Saharan Africa, Australasia, south Asia, Andean Latin America, and eastern Europe. We partially overcame prevalence data scarcity by using regional estimates and predictive covariates to produce estimates of the prevalence of benign prostatic hyperplasia in locations without local data. Our predictive covariates are, themselves, estimated for all year, age, and location combinations, generally with much stronger input databases than are available for benign prostatic hyperplasia, but with some uncertainty in estimation. Although the uncertainty intervals we report with our final prevalence estimates include uncertainty due to sampling and bias adjustment of input data, and uncertainty in the model fitting itself, the uncertainties in the covariate estimation processes are not reflected; future rounds of GBD should better account for covariate uncertainty. With regard to heterogeneity, the data we do have might differ on the basis of health-care-seeking behaviours and access to quality health care, rather than differences in underlying disease. This is partially addressed by processing hospital data as admission cause fractions, applying the fractions to estimates of health-care utilisation modelled from large household surveys,^41^ and then applying estimates of outpatient cases to inpatient admissions modelled from individual-level data using the Healthcare Access and Quality (HAQ) index as a predictor. Heterogeneity related to access was further accounted for in the USA by adjusting commercial claims data towards a general population reference standard through MR-BRT methods. Nonetheless, our ratios of outpatient cases to inpatient admissions were modelled from US data and might reflect a relationship between inpatient and outpatient care that is unique to the USA, and we did not have sufficient data to identify, quantify, and develop MR-BRT adjustments to account for all instances of heterogeneity due to access worldwide. We attempted to account for the most egregious heterogeneity by excluding outliers more than 2 MAD above or below the median, but this approach does not distinguish heterogeneity in the sources from heterogeneity in underlying disease. Regarding the general level of benign prostatic hyperplasia ascertainment in administrative data, these data sources might miss undiagnosed cases of benign prostatic hyperplasia that have not been seen by a medical provider. According to the Multinational Survey of Aging Male (MSAM-7) study conducted in the UK, the USA, France, Germany, the Netherlands, Italy, and Spain, only 19% of men with lower urinary tract symptoms sought care for urinary problems and only 10·2% had been medically treated.^68^ Another community-based study done in Singapore found that more than 70% of study participants with moderate-to-severe lower urinary tract symptoms did not seek care from a health-care provider.^72^ These studies suggest that we could be underestimating the prevalence of benign prostatic hyperplasia by relying on administrative data from clinical care encounters. In future rounds of GBD, we should augment our prevalence input data set via a systematic review of population-based studies, both to close gaps in countries without data and to facilitate nuanced quantification of the association between provider-diagnosed and overall benign prostatic hyperplasia prevalence and more accurately correct administrative data sources from diverse settings.

Second, data used for estimating the symptomatic proportion of benign prostatic hyperplasia prevalence and for estimating disability weights were more limited than prevalence data. Disability weights associated with health state descriptions used in GBD are derived from a series of face-to-face, telephone, and internet surveys conducted over several years and in nine countries, and reported in a series of publications.^56^, ^57^, ^58^ If these nine countries are poorly representative of the values surrounding health in other countries, this would misrepresent the disability globally. Estimation of the proportion of doctor-diagnosed symptomatic versus asymptomatic benign prostatic hyperplasia cases rests on an even smaller database; we made use of four community-based surveys of I-PSS scores to calculate the pooled proportion of symptomatic cases of benign prostatic hyperplasia. This approach makes two important assumptions: that the distribution of I-PSS scores in community-based samples is similar to the distribution of I-PSS scores among cases ascertained from administrative data, and that the distribution of I-PSS scores from these four surveys done in Japan, the USA, Scotland, and France is reflective of the global distribution.

Third, we acknowledge a pair of analytical limitations. Because of the GBD principle of assigning every death in our estimation framework to a single underlying cause of death, we elected to assign deaths related to benign prostatic hyperplasia to other diseases in the cascade of events that lead to death, and mortality related to benign prostatic hyperplasia was thus accounted for in various complications (eg, urinary tract infection or urolithiasis) and not included in the estimates for benign prostatic hyperplasia. Additionally, GBD estimation to date has largely focused on producing estimates for general populations defined only by year, age, sex, and location. We acknowledge the important matter of the disparate burden by race and ethnicity within these geographically defined populations. Future rounds of GBD should attempt to estimate the proportion of other deaths due to urological diseases that can be reasonably attributed to benign prostatic hyperplasia as an upstream risk factor and should disaggregate estimates of burden by race and ethnicity within populations.

The burden of benign prostatic hyperplasia is rising throughout the world, primarily due to population growth and ageing. Consequently, the male burden on the existing health-care system is expected to grow substantially in the coming years. This growth could be modified by control of upstream risk factors, and technologies exist to treat and mitigate the symptoms of benign prostatic hyperplasia. Coordinated and collaborative efforts from global, regional, and national policy makers, researchers, and advocates are needed to tackle this challenge.

## Data sharing

To download the source data and analytic code used in these analyses, please visit the Global Health Data Exchange GBD 2019 website.

## Declaration of interests

B Bikbov reports grants or contracts from the Lombardy Region outside the submitted work to their institution. T Garg reports support for attending meetings or travel from Siemens Healthineers outside the submitted work. N E Ismail reports unpaid leadership or fiduciary roles in board, society, committee, or advocacy groups with the Malaysian Academy of Pharmacy as a council member outside the submitted work. N Perico reports support for attending meetings or travel from and participation on a data safety monitoring board or advisory board with Bayer AG outside the submitted work. J A Singh reports consulting fees from Crealta Horizon, Medisys, Fidia, PK Med, Two Labs, Adept Field Solutions, Clinical Care Options, Clearview Healthcare Partners, Putnam Associates, Focus Forward, Navigant Consulting, Spherix, MedIQ, Jupiter Life Science, UBM, Trio Health, Medscape, WebMD, Practice Point Communications, the National Institutes of Health, and the American College of Rheumatology; payment or honoraria for lectures, presentations, speakers' bureaus, manuscript writing or educational events from Simply Speaking; support for attending meetings or travel from the steering committee of OMERACT; participation on a data safety monitoring board or advisory board with the US Food and Drug Administration Arthritis Advisory Committee; a leadership or fiduciary role in a board, society, committee or advocacy group, paid or unpaid, with OMERACT as a steering committee member, with the Veterans Affairs Rheumatology Field Advisory Committee as Chair (unpaid), and with the UAB Cochrane Musculoskeletal Group Satellite Center on Network Meta-analysis and editor and director (unpaid); stock or stock options in TPT Global Tech, Vaxart Pharmaceuticals, Atyu Biopharma, Adaptimmune Therapeutics, GeoVax Labs, Pieris Pharmaceuticals, Enzolytics, Seres Therapeutics, Tonix Pharmaceuticals and Charlotte's Web Holdings; and having previously owned stock options in Amarin, Viking, and Moderna Pharmaceuticals, all outside the submitted work. All other authors declare no competing interests.

## Supplementary Materials

Amharic translation of the abstract
