# Supplementary material for: The global, regional, and national burden of benign prostatic hyperplasia in 204 countries and territories from 2000 to 2019: a systematic analysis for the Global Burden of Disease Study 2019
Source: Lancet Healthy Longev. 2022 Nov;3(11):e754–76. doi: 10.1016/S2666-7568(22)00213-6 (PMC9640930; doi:10.1016/S2666-7568(22)00213-6)
Supplement: Amharic translation of the abstract [file mmc1.pdf]

THE LANCET
Healthy Longevity

Supplementary appendix 1

This translation in Amharic was submitted by the authors and we reproduce it as supplied. It has not been peer reviewed. *The Lancet*’s editorial processes have only been applied to the original in English, which should serve as reference for this manuscript.

ይህ የአማርኛ ትርጉም ተመራማሪዎቹ ባዘጋጁት መሰረት የቀረበ እና በሌላ ወገን ያልተገመገመ መሆኑን እናስገነዝባለን። በላንሴት የአርትዖት ሂደቶች የተገመገመው በእንግሊዝኛ ቋንቋ የቀረበው ጽሑፍ ብቻ በመሆኑ ለዚህ ጽሑፍ ማጣቀሻ ሆኖ ማገልገል ያለበት በእንግሊዝኛ የተዘጋጀ መሆኑን እናሳስባለን።

Supplement to: GBD 2019 Benign Prostatic Hyperplasia Collaborators. The global, regional, and national burden of benign prostatic hyperplasia in 204 countries and territories from 2000 to 2019: a systematic analysis for the Global Burden of Disease Study 2019. *Lancet Healthy Longev* 2022; published online Oct 20. https://doi.org/10.1016/S2666-7568(22)00213-6.

የፕሮስቴት እጢ በሽታ በዓለም አቀፍ፣ በክልሎች እና በሀገራት ላይ የሚያደርሰው የጤና ችግር ጥናት ትንተና ከ2000 እስከ 2019 (እ.ኤ.አ)፡ በዓለም አቀፍ የ2019 የበሽታዎች ጫና ጥናት

በ2019 የዓለም አቀፍ የፕሮስቴት እጢ ጫና አጥኚዎች ቡድን

የጥናቱ ዳራ፡ የፕሮስቴት እጢ (BPH) በዓለም ዙሪያ ባሉ ወንዶች የተለመደ የሽንት ቧንቧ በሽታ ነው። ነገር ግን አጠቃላይ ስለበሽታው ስርጭት በዓለም አቀፍ ፣ በክልላዊ እና በብሄራዊ ሁኔታ እና ከጊዜ ወደ ጊዜ ያለውን ለዉጥ የሚያሳዩ መረጃዎች በቂ አይደሉም ወይም ውሰን ናቸዉ። የፕሮስቴት እጢ ከአለምአቀፍ የጤና ጫና አሳዳሪ በሽታዎች፣ ጉዳቶች እና የአደጋ ምክንያቶች ጥናት (GBD 2019) መካከል አንዱ ነው። በመሆኑም በዚህ ጥናታዊ ጽሁፍ በአለም አቀፍ ደረጃ፣ በ21 ክልሎች እና በ204 ሀገራት እና ግዛቶች የበሽታውን ስርጭትና፤ በአካል ጉዳትና ሞት ያደረሰውን የጤና እክል (DALYs) እንዲሁም ከ2000 እስከ 2019 እ.ኤ.አ ያለዉን ለዉጥ ያካትታል።

የጥናት ስልትና ዘዴዎች፡ ይህ ጥናት በዓለም አቀፍ 2019 እ.ኤ.አ የበሽታዎች ጫና ጥናት (GBD 2019) ትንታኔና ሞዴሊንግ መሰራት የተሰራ ነው። ለጥናቱ የዋለው መረጃ በዋናነት ከሶስት አገሮች የተገኘ የበሽታው ስርጭት መረጃ እና ከይገባኛል ጥያቄዎች እንዲሁም ከ45 ቦታዎች ከተገኘ የሆስፒታል ታካሚዎች መረጃ ነው። የ Bayesia meta regression እና DisMod-MR 2.1 ሞዴሊንግ ስልቶችን በመጠቀም የፕሮስቴት እጢ ስርጭትን በዕድሜ፣ በቦታ እና ዓመታት ተንትነን አቅርበናል። በፕሮስቴት እጢ ምክንያት በአካል ጉዳተኝነት የኖሩት ዓመታት የተሰላው (YLDs) የአካል ጉዳተኝነት ክብደት ልክ በፕሮስቴት እጢ ስርጭት መጠን በማባዛት ነው። ከፕሮስቴት እጢ ጋር ተያይዞ በሞት የጠፋውን የህይወት ዘመን ስላላጠናን፣ በአካል ጉዳተኝነት የተስተካከለ የህይወት ዓመታት (DALYs) ከአካል ጉዳተኝነት ጋር የተኖረ ዓመታት YLD ጋር እኩል ነው።

የጥናት ውጤቶች፡ በዓለም ላይ እ.ኤ.አ. በ 2019፣ 94.0 ሚሊዮን (95% እርግጠኛ ያለመሆን ክፍተት 73.2–118) ተስፋፍቷል ነገር ግን በዓለም ላይ እ.ኤ.አ. በ 2000 51·1 ሚሊዮን (95% እርግጠኛ ያለመሆን ክፍተት 43·1 to 69·3) ነበር:: የዕድሜ ደረጃውን የጠበቀ /ያገናዘበ (age-standardized) ስርጭት ከ100,000 ሰዎች መካከል 2480 (1940-3090) ነው። ምንም እንኳን በዓለም አቀፍ ደረጃ የህሙማን ቁጥር ከ2000 እስከ 2019 በ70.5% (68.6–72.7) ቢጨምርም ፣ የዕድሜ ደረጃውን የጠበቀ የስርጭት መጠን ግን ለውጥ አላሳየም። በ2019 የዕድሜ ደረጃውን የጠበቀ የስርጭት መጠን በምስራቅ አውሮፓ ከታየወው 6480 (5130–8080) ከ100,000 ሰዎች እስከ 987(732–1320) በሰሜን አፍሪካ እና በመካከለኛው ምስራቅ ይደርሳል።

በ2000 እና 2019 መካከል ያለው በአካል ጉዳተኝነት የተስተካከለ የህይወት ዓመታት (DALYs) በቁጥር ደረጃ ስርጭት መጨመሩ በአምስቱም የሶሺዮ-ዲሞግራፊ መለኪያ (SDI) ደረጃዎች ላይ በሚገኙ ተመልክቷል።

በፍጥነት ጭማሪ ያሳዮት በሦስቱ የታችኛው SDI ደረጃዎች ለይ የሚገኙት ሲሆኑ፤ በመካከለኛ SDI በ94.7% (91.8-97.6) ጭማሪ ታይቷል፣ ዝቅተኛ-መካከለኛ SDI በ77.3% (74.1-81.2) ይጨምራል፣ እና ዝቅተኛ SDI በ 77.7% (72.9-83.2) ጨምሯል. በዚያ ጊዜ ውስጥ፣ የዕድሜ ደረጃውን የጠበቀ የDALY ተመኖች በትንሹ ተለውጧል, ነገር ግን ሦስቱ የታችኛው SDI ደረጃዎች ትንሽ ጭማሪ እና ሁለቱ ከፍ ያለ SDI (ከፍተኛ እና ከፍተኛ-መካከለኛ) ትንሽ ቀንሷል።

ትርጓሜ፡ በአብዛኛዎቹ የዓለም ክፍሎች የፕሮስቴት እጢ ስርጭት በሚያስደነግጥ ፍጥነት እየጨመረ ነው፣ በተለይም በማደግ ላይ ባሉ ሀገራት በአሁኑ ጊዜ የስነ-ሕዝብ እና የበሽታው ስርጭታዊ ለውጦች በፈጣን ሁኔታ እየታዩ ነው።. በዓለም ዙሪያ ብዙ ሰዎች ረዘም ላለ ጊዜ ሲኖሩ ፣ የበሽታው መጠን በሚቀጥሉት ዓመታት እየጨመረ ይሄዳል ብለን እንገምታለን።ይህ ደግሞ አጉልቶ የሚያሳየው ለወደፊት የጤና ስርዓት ስትራቴጂያዊ ክትትል እና ዕቅድ ማውጣት አስፈላጊ መሆኑን ነው።።
