# Supplementary material for: The global, regional, and national burden of benign prostatic hyperplasia in 204 countries and territories from 2000 to 2019: a systematic analysis for the Global Burden of Disease Study 2019
Source: Lancet Healthy Longev. 2022 Nov;3(11):e754–76. doi: 10.1016/S2666-7568(22)00213-6 (PMC9640930; doi:10.1016/S2666-7568(22)00213-6)
Supplement: Supplementary appendix 2 [file mmc2.pdf]

THE LANCET
Healthy Longevity

Supplementary appendix 2

This appendix formed part of the original submission and has been peer reviewed. We post it as supplied by the authors.

Supplement to: GBD 2019 Benign Prostatic Hyperplasia Collaborators. The global, regional, and national burden of benign prostatic hyperplasia in 204 countries and territories from 2000 to 2019: a systematic analysis for the Global Burden of Disease Study 2019. *Lancet Healthy Longev* 2022; published online Oct 20. https://doi.org/10.1016/S2666-7568(22)00213-6.

APPENDIX 1. SUPPLEMENTARY METHODOLOGY

Table 1. Geographical hierarchy

Location name	Short name	Region name	Super-region name
Armenia	ARM	Central Asia	Central Europe, eastern Europe, and central Asia
Azerbaijan	AZE	Central Asia	Central Europe, eastern Europe, and central Asia
Georgia	GEO	Central Asia	Central Europe, eastern Europe, and central Asia
Kazakhstan	KAZ	Central Asia	Central Europe, eastern Europe, and central Asia
Kyrgyzstan	KGZ	Central Asia	Central Europe, eastern Europe, and central Asia
Mongolia	MNG	Central Asia	Central Europe, eastern Europe, and central Asia
Tajikistan	TJK	Central Asia	Central Europe, eastern Europe, and central Asia
Turkmenistan	TKM	Central Asia	Central Europe, eastern Europe, and central Asia
Uzbekistan	UZB	Central Asia	Central Europe, eastern Europe, and central Asia
Albania	ALB	Central Europe	Central Europe, eastern Europe, and central Asia
Bosnia and Herzegovina	BIH	Central Europe	Central Europe, eastern Europe, and central Asia
Bulgaria	BGR	Central Europe	Central Europe, eastern Europe, and central Asia
Croatia	HRV	Central Europe	Central Europe, eastern Europe, and central Asia
Czech Republic	CZE	Central Europe	Central Europe, eastern Europe, and central Asia
Hungary	HUN	Central Europe	Central Europe, eastern Europe, and central Asia
Montenegro	MNE	Central Europe	Central Europe, eastern Europe, and central Asia
North Macedonia	MKD	Central Europe	Central Europe, eastern Europe, and central Asia
Poland	POL	Central Europe	Central Europe, eastern Europe, and central Asia
Romania	ROU	Central Europe	Central Europe, eastern Europe, and central Asia
Serbia	SRB	Central Europe	Central Europe, eastern Europe, and central Asia
Slovakia	SVK	Central Europe	Central Europe, eastern Europe, and central Asia
Slovenia	SVN	Central Europe	Central Europe, eastern Europe, and central Asia
Belarus	BLR	Eastern Europe	Central Europe, eastern Europe, and central Asia
Estonia	EST	Eastern Europe	Central Europe, eastern Europe, and central Asia
Latvia	LVA	Eastern Europe	Central Europe, eastern Europe, and central Asia

Lithuania	LTU	Eastern Europe	Central Europe, eastern Europe, and central Asia
Moldova	MDA	Eastern Europe	Central Europe, eastern Europe, and central Asia
Russia	RUS	Eastern Europe	Central Europe, eastern Europe, and central Asia
Ukraine	UKR	Eastern Europe	Central Europe, eastern Europe, and central Asia
Australia	AUS	Australasia	High-income
New Zealand	NZL	Australasia	High-income
Brunei	BRN	High-income Asia Pacific	High-income
Japan	JPN	High-income Asia Pacific	High-income
South Korea	KOR	High-income Asia Pacific	High-income
Singapore	SGP	High-income Asia Pacific	High-income
Canada	CAN	High-income North America	High-income
Greenland	GRL	High-income North America	High-income
USA	USA	High-income North America	High-income
Argentina	ARG	Southern Latin America	High-income
Chile	CHL	Southern Latin America	High-income
Uruguay	URY	Southern Latin America	High-income
Andorra	AND	Western Europe	High-income
Austria	AUT	Western Europe	High-income
Belgium	BEL	Western Europe	High-income
Cyprus	CYP	Western Europe	High-income
Denmark	DNK	Western Europe	High-income
Finland	FIN	Western Europe	High-income
France	FRA	Western Europe	High-income
Germany	DEU	Western Europe	High-income
Greece	GRC	Western Europe	High-income
Iceland	ISL	Western Europe	High-income
Ireland	IRL	Western Europe	High-income
Israel	ISR	Western Europe	High-income
Italy	ITA	Western Europe	High-income
Luxembourg	LUX	Western Europe	High-income
Malta	MLT	Western Europe	High-income

Monaco	MCO	Western Europe	High-income
Netherlands	NLD	Western Europe	High-income
Norway	NOR	Western Europe	High-income
Portugal	PRT	Western Europe	High-income
San Marino	SMR	Western Europe	High-income
Spain	ESP	Western Europe	High-income
Sweden	SWE	Western Europe	High-income
Switzerland	CHE	Western Europe	High-income
UK	GBR	Western Europe	High-income
Bolivia	BOL	Andean Latin America	Latin America and Caribbean
Ecuador	ECU	Andean Latin America	Latin America and Caribbean
Peru	PER	Andean Latin America	Latin America and Caribbean
Antigua and Barbuda	ATG	Caribbean	Latin America and Caribbean
The Bahamas	BHS	Caribbean	Latin America and Caribbean
Barbados	BRB	Caribbean	Latin America and Caribbean
Belize	BLZ	Caribbean	Latin America and Caribbean
Bermuda	BMU	Caribbean	Latin America and Caribbean
Cuba	CUB	Caribbean	Latin America and Caribbean
Dominica	DMA	Caribbean	Latin America and Caribbean
Dominican Republic	DOM	Caribbean	Latin America and Caribbean
Grenada	GRD	Caribbean	Latin America and Caribbean
Guyana	GUY	Caribbean	Latin America and Caribbean
Haiti	HTI	Caribbean	Latin America and Caribbean
Jamaica	JAM	Caribbean	Latin America and Caribbean
Puerto Rico	PRI	Caribbean	Latin America and Caribbean
Saint Kitts and Nevis	KNA	Caribbean	Latin America and Caribbean
Saint Lucia	LCA	Caribbean	Latin America and Caribbean
Saint Vincent and the Grenadines	VCT	Caribbean	Latin America and Caribbean
Suriname	SUR	Caribbean	Latin America and Caribbean
Trinidad and Tobago	TTO	Caribbean	Latin America and Caribbean
Virgin Islands	VIR	Caribbean	Latin America and Caribbean

Colombia	COL	Central Latin America	Latin America and Caribbean
Costa Rica	CRI	Central Latin America	Latin America and Caribbean
El Salvador	SLV	Central Latin America	Latin America and Caribbean
Guatemala	GTM	Central Latin America	Latin America and Caribbean
Honduras	HND	Central Latin America	Latin America and Caribbean
Mexico	MEX	Central Latin America	Latin America and Caribbean
Nicaragua	NIC	Central Latin America	Latin America and Caribbean
Panama	PAN	Central Latin America	Latin America and Caribbean
Venezuela	VEN	Central Latin America	Latin America and Caribbean
Brazil	BRA	Tropical Latin America	Latin America and Caribbean
Paraguay	PRY	Tropical Latin America	Latin America and Caribbean
Afghanistan	AFG	North Africa and Middle East	North Africa and Middle East
Algeria	DZA	North Africa and Middle East	North Africa and Middle East
Bahrain	BHR	North Africa and Middle East	North Africa and Middle East
Egypt	EGY	North Africa and Middle East	North Africa and Middle East
Iran	IRN	North Africa and Middle East	North Africa and Middle East
Iraq	IRQ	North Africa and Middle East	North Africa and Middle East
Jordan	JOR	North Africa and Middle East	North Africa and Middle East
Kuwait	KWT	North Africa and Middle East	North Africa and Middle East
Lebanon	LBN	North Africa and Middle East	North Africa and Middle East
Libya	LBY	North Africa and Middle East	North Africa and Middle East
Morocco	MAR	North Africa and Middle East	North Africa and Middle East
Oman	OMN	North Africa and Middle East	North Africa and Middle East
Palestine	PSE	North Africa and Middle East	North Africa and Middle East
Qatar	QAT	North Africa and Middle East	North Africa and Middle East
Saudi Arabia	SAU	North Africa and Middle East	North Africa and Middle East
Sudan	SDN	North Africa and Middle East	North Africa and Middle East
Syria	SYR	North Africa and Middle East	North Africa and Middle East
Tunisia	TUN	North Africa and Middle East	North Africa and Middle East
Turkey	TUR	North Africa and Middle East	North Africa and Middle East
United Arab Emirates	ARE	North Africa and Middle East	North Africa and Middle East

Yemen	YEM	North Africa and Middle East	North Africa and Middle East
Bangladesh	BGD	South Asia	South Asia
Bhutan	BTN	South Asia	South Asia
India	IND	South Asia	South Asia
Nepal	NPL	South Asia	South Asia
Pakistan	PAK	South Asia	South Asia
China	CHN	East Asia	Southeast Asia, east Asia, and Oceania
North Korea	PRK	East Asia	Southeast Asia, east Asia, and Oceania
Taiwan (province of China)	TWN	East Asia	Southeast Asia, east Asia, and Oceania
American Samoa	ASM	Oceania	Southeast Asia, east Asia, and Oceania
Cook Islands	COK	Oceania	Southeast Asia, east Asia, and Oceania
Fiji	FJI	Oceania	Southeast Asia, east Asia, and Oceania
Guam	GUM	Oceania	Southeast Asia, east Asia, and Oceania
Kiribati	KIR	Oceania	Southeast Asia, east Asia, and Oceania
Marshall Islands	MHL	Oceania	Southeast Asia, east Asia, and Oceania
Federated States of Micronesia	FSM	Oceania	Southeast Asia, east Asia, and Oceania
Nauru	NRU	Oceania	Southeast Asia, east Asia, and Oceania
Niue	NIU	Oceania	Southeast Asia, east Asia, and Oceania
Northern Mariana Islands	MNP	Oceania	Southeast Asia, east Asia, and Oceania
Palau	PLW	Oceania	Southeast Asia, east Asia, and Oceania
Papua New Guinea	PNG	Oceania	Southeast Asia, east Asia, and Oceania
Samoa	WSM	Oceania	Southeast Asia, east Asia, and Oceania
Solomon Islands	SLB	Oceania	Southeast Asia, east Asia, and Oceania
Tokelau	TKL	Oceania	Southeast Asia, east Asia, and Oceania
Tonga	TON	Oceania	Southeast Asia, east Asia, and Oceania
Tuvalu	TUV	Oceania	Southeast Asia, east Asia, and Oceania
Vanuatu	VUT	Oceania	Southeast Asia, east Asia, and Oceania
Cambodia	KHM	Southeast Asia	Southeast Asia, east Asia, and Oceania
Indonesia	IDN	Southeast Asia	Southeast Asia, east Asia, and Oceania
Laos	LAO	Southeast Asia	Southeast Asia, east Asia, and Oceania
Malaysia	MYS	Southeast Asia	Southeast Asia, east Asia, and Oceania

Maldives	MDV	Southeast Asia	Southeast Asia, east Asia, and Oceania
Mauritius	MUS	Southeast Asia	Southeast Asia, east Asia, and Oceania
Myanmar	MMR	Southeast Asia	Southeast Asia, east Asia, and Oceania
Philippines	PHL	Southeast Asia	Southeast Asia, east Asia, and Oceania
Seychelles	SYC	Southeast Asia	Southeast Asia, east Asia, and Oceania
Sri Lanka	LKA	Southeast Asia	Southeast Asia, east Asia, and Oceania
Thailand	THA	Southeast Asia	Southeast Asia, east Asia, and Oceania
Timor-Leste	TLS	Southeast Asia	Southeast Asia, east Asia, and Oceania
Vietnam	VNM	Southeast Asia	Southeast Asia, east Asia, and Oceania
Angola	AGO	Central sub-Saharan Africa	Sub-Saharan Africa
Central African Republic	CAF	Central sub-Saharan Africa	Sub-Saharan Africa
Congo (Brazzaville)	COG	Central sub-Saharan Africa	Sub-Saharan Africa
DR Congo	COD	Central sub-Saharan Africa	Sub-Saharan Africa
Equatorial Guinea	GNQ	Central sub-Saharan Africa	Sub-Saharan Africa
Gabon	GAB	Central sub-Saharan Africa	Sub-Saharan Africa
Burundi	BDI	Eastern sub-Saharan Africa	Sub-Saharan Africa
Comoros	COM	Eastern sub-Saharan Africa	Sub-Saharan Africa
Djibouti	DJI	Eastern sub-Saharan Africa	Sub-Saharan Africa
Eritrea	ERI	Eastern sub-Saharan Africa	Sub-Saharan Africa
Ethiopia	ETH	Eastern sub-Saharan Africa	Sub-Saharan Africa
Kenya	KEN	Eastern sub-Saharan Africa	Sub-Saharan Africa
Madagascar	MDG	Eastern sub-Saharan Africa	Sub-Saharan Africa
Malawi	MWI	Eastern sub-Saharan Africa	Sub-Saharan Africa
Mozambique	MOZ	Eastern sub-Saharan Africa	Sub-Saharan Africa
Rwanda	RWA	Eastern sub-Saharan Africa	Sub-Saharan Africa
Somalia	SOM	Eastern sub-Saharan Africa	Sub-Saharan Africa
South Sudan	SSD	Eastern sub-Saharan Africa	Sub-Saharan Africa
Uganda	UGA	Eastern sub-Saharan Africa	Sub-Saharan Africa
Tanzania	TZA	Eastern sub-Saharan Africa	Sub-Saharan Africa
Zambia	ZMB	Eastern sub-Saharan Africa	Sub-Saharan Africa
Botswana	BWA	Southern sub-Saharan Africa	Sub-Saharan Africa

eSwatini	SWZ	Southern sub-Saharan Africa	Sub-Saharan Africa
Lesotho	LSO	Southern sub-Saharan Africa	Sub-Saharan Africa
Namibia	NAM	Southern sub-Saharan Africa	Sub-Saharan Africa
South Africa	ZAF	Southern sub-Saharan Africa	Sub-Saharan Africa
Zimbabwe	ZWE	Southern sub-Saharan Africa	Sub-Saharan Africa
Benin	BEN	Western sub-Saharan Africa	Sub-Saharan Africa
Burkina Faso	BFA	Western sub-Saharan Africa	Sub-Saharan Africa
Cape Verde	CPV	Western sub-Saharan Africa	Sub-Saharan Africa
Cameroon	CMR	Western sub-Saharan Africa	Sub-Saharan Africa
Chad	TCD	Western sub-Saharan Africa	Sub-Saharan Africa
Côte d’Ivoire	CIV	Western sub-Saharan Africa	Sub-Saharan Africa
The Gambia	GMB	Western sub-Saharan Africa	Sub-Saharan Africa
Ghana	GHA	Western sub-Saharan Africa	Sub-Saharan Africa
Guinea	GIN	Western sub-Saharan Africa	Sub-Saharan Africa
Guinea-Bissau	GNB	Western sub-Saharan Africa	Sub-Saharan Africa
Liberia	LBR	Western sub-Saharan Africa	Sub-Saharan Africa
Mali	MLI	Western sub-Saharan Africa	Sub-Saharan Africa
Mauritania	MRT	Western sub-Saharan Africa	Sub-Saharan Africa
Niger	NER	Western sub-Saharan Africa	Sub-Saharan Africa
Nigeria	NGA	Western sub-Saharan Africa	Sub-Saharan Africa
São Tomé and Príncipe	STP	Western sub-Saharan Africa	Sub-Saharan Africa
Senegal	SEN	Western sub-Saharan Africa	Sub-Saharan Africa
Sierra Leone	SLE	Western sub-Saharan Africa	Sub-Saharan Africa
Togo	TGO	Western sub-Saharan Africa	Sub-Saharan Africa

Table 2. List of countries by SDI quintile

Country name	SDI quintile
Afghanistan	Low SDI
Benin	Low SDI
Burkina Faso	Low SDI
Burundi	Low SDI
Central African Republic	Low SDI
Chad	Low SDI
Comoros	Low SDI
Côte d’Ivoire	Low SDI
Democratic Republic of the Congo	Low SDI
Eritrea	Low SDI
Gambia	Low SDI
Guinea	Low SDI
Guinea-Bissau	Low SDI
Haiti	Low SDI
Liberia	Low SDI
Madagascar	Low SDI
Malawi	Low SDI
Mali	Low SDI
Mozambique	Low SDI
Nepal	Low SDI
Niger	Low SDI
Papua New Guinea	Low SDI
Rwanda	Low SDI
Senegal	Low SDI
Sierra Leone	Low SDI
Solomon Islands	Low SDI
Somalia	Low SDI
South Sudan	Low SDI

Togo	Low SDI
Uganda	Low SDI
United Republic of Tanzania	Low SDI
Yemen	Low SDI
Angola	Low-middle SDI
Bangladesh	Low-middle SDI
Belize	Low-middle SDI
Bhutan	Low-middle SDI
Bolivia	Low-middle SDI
Cabo Verde	Low-middle SDI
Cambodia	Low-middle SDI
Cameroon	Low-middle SDI
Congo	Low-middle SDI
North Korea	Low-middle SDI
Djibouti	Low-middle SDI
Dominican Republic	Low-middle SDI
El Salvador	Low-middle SDI
Eswatini	Low-middle SDI
Ghana	Low-middle SDI
Guatemala	Low-middle SDI
Honduras	Low-middle SDI
Kiribati	Low-middle SDI
Kyrgyzstan	Low-middle SDI
Laos	Low-middle SDI
Lesotho	Low-middle SDI
Maldives	Low-middle SDI
Marshall Islands	Low-middle SDI
Mauritania	Low-middle SDI
Federated States of Micronesia	Low-middle SDI
Mongolia	Low-middle SDI
Morocco	Low-middle SDI

Myanmar	Low-middle SDI
Nicaragua	Low-middle SDI
Palestine	Low-middle SDI
São Tomé and Príncipe	Low-middle SDI
Sudan	Low-middle SDI
Tajikistan	Low-middle SDI
Timor-Leste	Low-middle SDI
Tuvalu	Low-middle SDI
Vanuatu	Low-middle SDI
Venezuela	Low-middle SDI
Zambia	Low-middle SDI
Zimbabwe	Low-middle SDI
Albania	Middle SDI
Algeria	Middle SDI
Armenia	Middle SDI
Azerbaijan	Middle SDI
Botswana	Middle SDI
Colombia	Middle SDI
Costa Rica	Middle SDI
Cuba	Middle SDI
Ecuador	Middle SDI
Egypt	Middle SDI
Equatorial Guinea	Middle SDI
Fiji	Middle SDI
Gabon	Middle SDI
Grenada	Middle SDI
Guyana	Middle SDI
Iraq	Middle SDI
Jamaica	Middle SDI
Namibia	Middle SDI
Nauru	Middle SDI

Panama	Middle SDI
Paraguay	Middle SDI
Peru	Middle SDI
Saint Lucia	Middle SDI
Saint Vincent and the Grenadines	Middle SDI
Samoa	Middle SDI
Suriname	Middle SDI
Syria	Middle SDI
Thailand	Middle SDI
Tokelau	Middle SDI
Tonga	Middle SDI
Tunisia	Middle SDI
Turkmenistan	Middle SDI
Uzbekistan	Middle SDI
Vietnam	Middle SDI
American Samoa	High-middle SDI
Antigua and Barbuda	High-middle SDI
Argentina	High-middle SDI
Bahamas	High-middle SDI
Bahrain	High-middle SDI
Barbados	High-middle SDI
Belarus	High-middle SDI
Bosnia and Herzegovina	High-middle SDI
Bulgaria	High-middle SDI
Chile	High-middle SDI
Cook Islands	High-middle SDI
Croatia	High-middle SDI
Dominica	High-middle SDI
Georgia	High-middle SDI
Greece	High-middle SDI
Greenland	High-middle SDI

Hungary	High-middle SDI
Israel	High-middle SDI
Jordan	High-middle SDI
Kazakhstan	High-middle SDI
Lebanon	High-middle SDI
Libya	High-middle SDI
Malaysia	High-middle SDI
Malta	High-middle SDI
Mauritius	High-middle SDI
Montenegro	High-middle SDI
Niue	High-middle SDI
North Macedonia	High-middle SDI
Northern Mariana Islands	High-middle SDI
Oman	High-middle SDI
Palau	High-middle SDI
Portugal	High-middle SDI
Republic of Moldova	High-middle SDI
Romania	High-middle SDI
Saint Kitts and Nevis	High-middle SDI
Serbia	High-middle SDI
Seychelles	High-middle SDI
Spain	High-middle SDI
Sri Lanka	High-middle SDI
Trinidad and Tobago	High-middle SDI
Turkey	High-middle SDI
Virgin Islands	High-middle SDI
Uruguay	High-middle SDI
Andorra	High SDI
Australia	High SDI
Austria	High SDI
Belgium	High SDI

Bermuda	High SDI
Brunei	High SDI
Canada	High SDI
Cyprus	High SDI
Czechia	High SDI
Denmark	High SDI
Estonia	High SDI
Finland	High SDI
France	High SDI
Germany	High SDI
Guam	High SDI
Iceland	High SDI
Ireland	High SDI
Kuwait	High SDI
Latvia	High SDI
Lithuania	High SDI
Luxembourg	High SDI
Monaco	High SDI
Netherlands	High SDI
Puerto Rico	High SDI
Qatar	High SDI
South Korea	High SDI
San Marino	High SDI
Saudi Arabia	High SDI
Singapore	High SDI
Slovakia	High SDI
Slovenia	High SDI
Switzerland	High SDI
Taiwan (province of China)	High SDI
United Arab Emirates	High SDI

Table 3. List of prevalence data sources for benign prostatic hyperplasia

A) Total data sources considered

Country name	Data source	Year range
Austria	Federal Ministry of Health (Austria), Statistics Austria. Austria Hospital Inpatient Discharges 1989-1992.	1988-1992
Austria	Federal Ministry of Health (Austria), Statistics Austria. Austria Hospital Inpatient Discharges 1993-1997.	1993-1997
Austria	Federal Ministry of Health (Austria), Statistics Austria. Austria Hospital Inpatient Discharges 1998-2002.	1998-2002
Austria	Federal Ministry of Health (Austria), Statistics Austria. Austria Hospital Inpatient Discharges 2003-2007.	2003-2007
Austria	Federal Ministry of Health (Austria), Statistics Austria. Austria Hospital Inpatient Discharges 2008-2012.	2008-2012
Austria	Federal Ministry of Health (Austria), Statistics Austria. Austria Hospital Inpatient Discharges 2013-2014.	2013-2017
Belgium	World Health Organization Regional Office for Europe (WHO/Europe). European Hospital Morbidity Database, 2008. Copenhagen, Denmark: World Health Organization Regional Office for Europe (WHO/Europe).	2003-2007
Belgium	World Health Organization Regional Office for Europe (WHO/Europe). European Hospital Morbidity Database, 2008. Copenhagen, Denmark: World Health Organization Regional Office for Europe (WHO/Europe).	2008-2012
Botswana	Ministry of Health (Botswana). Botswana Health Management Data System 2008-2009.	2003-2007
Botswana	Ministry of Health (Botswana). Botswana Health Management Data System 2008-2009.	2008-2012
Brazil	Ministry of Health (Brazil). Brazil Hospital Information System 1997. Rio de Janeiro, Brazil: Ministry of Health (Brazil).	1993-1997

Brazil	Ministry of Health (Brazil). Brazil Hospital Information System 1998-2002.	1998-2002
Brazil	Ministry of Health (Brazil). Brazil Hospital Information System 2003-2007.	2003-2007
Brazil	Ministry of Health (Brazil). Brazil Hospital Information System 2008-2012.	2008-2012
Brazil	Ministry of Health (Brazil). Brazil Hospital Information System 2013-2014.	2013-2017
Chile	Ministry of Health (Chile). Chile Hospital Discharges 2001-2002.	1998-2002
Chile	Ministry of Health (Chile). Chile Hospital Discharges 2003-2007.	2003-2007
Chile	Ministry of Health (Chile). Chile Hospital Discharges 2008-2012.	2008-2012
China	Center for Health Statistics and Information, National Health and Family Planning Commission (China), Shanghai Municipal Center for Disease Control and Prevention (Shanghai CDC), Shanghai Health Information Center. China Hospital Inpatient Data 2013-2016.	2013-2017
Croatia	World Health Organization Regional Office for Europe (WHO/Europe). European Hospital Morbidity Database, 2008. Copenhagen, Denmark: World Health Organization Regional Office for Europe (WHO/Europe).	1998-2002
Croatia	World Health Organization Regional Office for Europe (WHO/Europe). European Hospital Morbidity Database, 2008. Copenhagen, Denmark: World Health Organization Regional Office for Europe (WHO/Europe).	2003-2007
Croatia	World Health Organization Regional Office for Europe (WHO/Europe). European Hospital Morbidity Database, 2008. Copenhagen, Denmark: World Health Organization Regional Office for Europe (WHO/Europe).	2008-2012
Croatia	World Health Organization Regional Office for Europe (WHO/Europe). European Hospital Morbidity Database, 2008. Copenhagen, Denmark: World Health Organization Regional Office for Europe (WHO/Europe).	2013-2017
Cyprus	World Health Organization Regional Office for Europe (WHO/Europe). European Hospital Morbidity Database, 2008. Copenhagen, Denmark: World Health Organization Regional Office for Europe (WHO/Europe).	2003-2007
Cyprus	World Health Organization Regional Office for Europe (WHO/Europe). European Hospital Morbidity Database, 2008. Copenhagen, Denmark: World Health Organization Regional Office for Europe (WHO/Europe).	2008-2012

Czechia	World Health Organization Regional Office for Europe (WHO/Europe). European Hospital Morbidity Database, 2008. Copenhagen, Denmark: World Health Organization Regional Office for Europe (WHO/Europe).	2008-2012
Denmark	World Health Organization Regional Office for Europe (WHO/Europe). European Hospital Morbidity Database, 2008. Copenhagen, Denmark: World Health Organization Regional Office for Europe (WHO/Europe).	2003-2007
Ecuador	National Institute of Statistics and Censuses (Ecuador). Ecuador Hospital Inpatient Discharges 1993-1997.	1993-1997
Ecuador	National Institute of Statistics and Censuses (Ecuador). Ecuador Hospital Inpatient Discharges 1998-2002.	1998-2002
Ecuador	National Institute of Statistics and Censuses (Ecuador). Ecuador Hospital Inpatient Discharges 2003-2007.	2003-2007
Ecuador	National Institute of Statistics and Censuses (Ecuador). Ecuador Hospital Inpatient Discharges 2008-2012.	2008-2012
Ecuador	National Institute of Statistics and Censuses (Ecuador). Ecuador Hospital Inpatient Discharges 2013-2014.	2013-2017
Ecuador	National Institute of Statistics and Censuses (Ecuador). Ecuador Statistical Registry of Hospital Beds and Expenses 2015-2017.	2015-2017
England	NHS England. United Kingdom - England Hospital Episode Statistics 2001-2002.	1998-2002
England	NHS England. United Kingdom - England Hospital Episode Statistics 2003-2007.	2003-2007
England	NHS England. United Kingdom - England Hospital Episode Statistics 2008-2012.	2008-2012
England	NHS England. United Kingdom - England Hospital Episode Statistics 2013-2014.	2013-2017
Finland	World Health Organization Regional Office for Europe (WHO/Europe). European Hospital Morbidity Database, 2008. Copenhagen, Denmark: World Health Organization Regional Office for Europe (WHO/Europe).	1998-2002
Finland	World Health Organization Regional Office for Europe (WHO/Europe). European Hospital Morbidity Database, 2008. Copenhagen, Denmark: World Health Organization Regional Office for Europe (WHO/Europe).	2003-2007
Finland	World Health Organization Regional Office for Europe (WHO/Europe). European Hospital Morbidity Database, 2008. Copenhagen, Denmark: World Health Organization Regional Office for Europe (WHO/Europe).	2008-2012

Georgia	National Center for Disease Control and Public Health (Georgia). Georgia Hospital Data 2013-2014.	2013-2017
Georgia	National Center for Disease Control and Public Health (Georgia). Georgia Hospital Discharges 2016-2017.	2016-2017
Germany	Federal Statistical Office (Germany). Germany Hospital Discharges by Diagnosis 2009. Wiesbaden, Germany: Federal Statistical Office (Germany), 2011.	2008-2012
Iceland	World Health Organization Regional Office for Europe (WHO/Europe). European Hospital Morbidity Database, 2008. Copenhagen, Denmark: World Health Organization Regional Office for Europe (WHO/Europe).	2008-2012
India	Nazareth Hospital, Shillong, JSS Hospital, Mysore, King George's Medical University (India). India Hospital Inpatient Data 2014-2017.	2013-2017
India	Nazareth Hospital, Shillong. India - Shillong Nazareth Hospital Inpatient Discharges 2014.	2013-2017
India	St. John's Medical College Hospital (India). India - Bangalore St. John's Medical College Hospital Inpatient Data 2017.	2013-2017
Indonesia	Ministry of Health (Indonesia). Indonesia Integrated Hospital Data 2013. Jakarta, Indonesia: Ministry of Health (Indonesia), 2014.	2013-2017
Iran (Islamic Republic of)	Ministry of Health and Medical Education (Iran). Iran Hospital Data 2001-2010.	1998-2002
Iran (Islamic Republic of)	Ministry of Health and Medical Education (Iran). Iran Hospital Data 2001-2010.	2003-2007
Iran (Islamic Republic of)	Ministry of Health and Medical Education (Iran). Iran Hospital Data 2001-2010.	2008-2012
Italy	Ministry of Health (Italy). Italy Hospital Inpatient Discharges 2005-2007.	2003-2007
Italy	Ministry of Health (Italy). Italy Hospital Inpatient Discharges 2008-2012.	2008-2012
Italy	Ministry of Health (Italy). Italy Hospital Inpatient Discharges 2013-2016.	2013-2017
Japan	Ministry of Health, Labour and Welfare (Japan). Japan Diagnosis Procedure Combination Database 2010-2012.	2008-2012
Japan	Ministry of Health, Labour and Welfare (Japan). Japan Diagnosis Procedure Combination Database 2013-2015.	2013-2017
Jordan	Ministry of Health (Jordan). Jordan Al-Bashir Hospital Discharges 2016.	2013-2017
Kenya	Ministry of Health (Kenya). Kenya National Inpatient Morbidity and Mortality Statistics 1999.	1998-2002

Kyrgyzstan	Mandatory Health Insurance Fund (Kyrgyzstan). Kyrgyzstan - Bishkek Clinical-Related Groups Hospital Claims 2012.	2008-2012
Latvia	World Health Organization Regional Office for Europe (WHO/Europe). European Hospital Morbidity Database, 2008. Copenhagen, Denmark: World Health Organization Regional Office for Europe (WHO/Europe).	2003-2007
Latvia	World Health Organization Regional Office for Europe (WHO/Europe). European Hospital Morbidity Database, 2008. Copenhagen, Denmark: World Health Organization Regional Office for Europe (WHO/Europe).	2008-2012
Lithuania	World Health Organization Regional Office for Europe (WHO/Europe). European Hospital Morbidity Database, 2008. Copenhagen, Denmark: World Health Organization Regional Office for Europe (WHO/Europe).	1998-2002
Lithuania	World Health Organization Regional Office for Europe (WHO/Europe). European Hospital Morbidity Database, 2008. Copenhagen, Denmark: World Health Organization Regional Office for Europe (WHO/Europe).	2003-2007
Lithuania	World Health Organization Regional Office for Europe (WHO/Europe). European Hospital Morbidity Database, 2008. Copenhagen, Denmark: World Health Organization Regional Office for Europe (WHO/Europe).	2008-2012
Luxembourg	World Health Organization Regional Office for Europe (WHO/Europe). European Hospital Morbidity Database, 2008. Copenhagen, Denmark: World Health Organization Regional Office for Europe (WHO/Europe).	1998-2002
Luxembourg	World Health Organization Regional Office for Europe (WHO/Europe). European Hospital Morbidity Database, 2008. Copenhagen, Denmark: World Health Organization Regional Office for Europe (WHO/Europe).	2003-2007
Luxembourg	World Health Organization Regional Office for Europe (WHO/Europe). European Hospital Morbidity Database, 2008. Copenhagen, Denmark: World Health Organization Regional Office for Europe (WHO/Europe).	2008-2012
Malta	World Health Organization Regional Office for Europe (WHO/Europe). European Hospital Morbidity Database, 2008. Copenhagen, Denmark: World Health Organization Regional Office for Europe (WHO/Europe).	2003-2007

Malta	World Health Organization Regional Office for Europe (WHO/Europe). European Hospital Morbidity Database, 2008. Copenhagen, Denmark: World Health Organization Regional Office for Europe (WHO/Europe).	2008-2012
Mexico	Ministry of Health (Mexico). Mexico Ministry of Health Hospital Discharges 2000-2002.	1998-2002
Mexico	Ministry of Health (Mexico). Mexico Ministry of Health Hospital Discharges 2003-2007.	2003-2007
Mexico	Ministry of Health (Mexico). Mexico Ministry of Health Hospital Discharges 2008-2012.	2008-2012
Mexico	Ministry of Health (Mexico). Mexico Ministry of Health Hospital Discharges 2013-2015.	2013-2017
Nepal	Department of Health Services, Ministry of Health and Population (Nepal). Nepal Hospital Inpatient Discharges 2010-2012.	2008-2012
Nepal	Department of Health Services, Ministry of Health and Population (Nepal). Nepal Hospital Inpatient Discharges 2013-2014.	2013-2017
New Zealand	Ministry of Health (New Zealand). New Zealand National Minimum Dataset 2000-2002.	1998-2002
New Zealand	Ministry of Health (New Zealand). New Zealand National Minimum Dataset 2003-2007.	2003-2007
New Zealand	Ministry of Health (New Zealand). New Zealand National Minimum Dataset 2008-2012.	2008-2012
New Zealand	Ministry of Health (New Zealand). New Zealand National Minimum Dataset 2013-2014.	2013-2017
New Zealand	Ministry of Health (New Zealand). New Zealand National Minimum Dataset 2016-2017.	2016-2017
Norway	Norwegian Directorate of Health. Norway Patient Register 2008-2012.	2008-2012
Philippines	Philippine Health Insurance Corporation. Philippine Health Insurance Corporation Claims 2013-2016.	2013-2017
Poland	World Health Organization Regional Office for Europe (WHO/Europe). European Hospital Morbidity Database, 2008. Copenhagen, Denmark: World Health Organization Regional Office for Europe (WHO/Europe).	2003-2007
Poland	World Health Organization Regional Office for Europe (WHO/Europe). European Hospital Morbidity Database, 2008. Copenhagen, Denmark: World Health Organization Regional Office for Europe (WHO/Europe).	2008-2012
Poland	National Health Fund (Poland). Poland National Health Fund Patient Claims 2015.	2015-2015
Poland	National Health Fund (Poland). Poland National Health Fund Patient Claims 2016.	2016-2016

Poland	National Health Fund (Poland). Poland National Health Fund Patient Claims 2017.	2017-2017
Portugal	Ministry of Health (Portugal). Portugal Hospital Inpatient Discharges 2015.	2013-2017
Qatar	Hamad Medical Corporation (Qatar). Qatar - Annual Inpatients Discharge Abstract: Hamad General Hospital 2002. Doha, Qatar: Hamad Medical Corporation (Qatar).	1998-2002
Qatar	Hamad Medical Corporation (Qatar). Qatar - Annual Inpatients Discharge Abstract: Hamad General Hospital and Women's Hospital 2003. Doha, Qatar: Hamad Medical Corporation (Qatar).	2003-2007
Romania	World Health Organization Regional Office for Europe (WHO/Europe). European Hospital Morbidity Database, 2008. Copenhagen, Denmark: World Health Organization Regional Office for Europe (WHO/Europe).	2008-2012
Serbia	World Health Organization Regional Office for Europe (WHO/Europe). European Hospital Morbidity Database, 2008. Copenhagen, Denmark: World Health Organization Regional Office for Europe (WHO/Europe).	2008-2012
Slovakia	World Health Organization Regional Office for Europe (WHO/Europe). European Hospital Morbidity Database, 2008. Copenhagen, Denmark: World Health Organization Regional Office for Europe (WHO/Europe).	1998-2002
Slovakia	World Health Organization Regional Office for Europe (WHO/Europe). European Hospital Morbidity Database, 2008. Copenhagen, Denmark: World Health Organization Regional Office for Europe (WHO/Europe).	2003-2007
Slovakia	World Health Organization Regional Office for Europe (WHO/Europe). European Hospital Morbidity Database, 2008. Copenhagen, Denmark: World Health Organization Regional Office for Europe (WHO/Europe).	2008-2012
Slovenia	World Health Organization Regional Office for Europe (WHO/Europe). European Hospital Morbidity Database, 2008. Copenhagen, Denmark: World Health Organization Regional Office for Europe (WHO/Europe).	2003-2007
Slovenia	World Health Organization Regional Office for Europe (WHO/Europe). European Hospital Morbidity Database, 2008. Copenhagen, Denmark: World Health Organization Regional Office for Europe (WHO/Europe).	2008-2012
Sweden	National Board of Health and Welfare (Sweden). Sweden National Patient Register 1998-2002.	1998-2002

Sweden	National Board of Health and Welfare (Sweden). Sweden National Patient Register 2003-2007.	2003-2007
Sweden	National Board of Health and Welfare (Sweden). Sweden National Patient Register 2008-2012.	2008-2012
Sweden	National Board of Health and Welfare (Sweden). Sweden National Patient Register 2013-2016.	2013-2017
Switzerland	World Health Organization Regional Office for Europe (WHO/Europe). European Hospital Morbidity Database, 2008. Copenhagen, Denmark: World Health Organization Regional Office for Europe (WHO/Europe).	1998-2002
Switzerland	World Health Organization Regional Office for Europe (WHO/Europe). European Hospital Morbidity Database, 2008. Copenhagen, Denmark: World Health Organization Regional Office for Europe (WHO/Europe).	2003-2007
Switzerland	World Health Organization Regional Office for Europe (WHO/Europe). European Hospital Morbidity Database, 2008. Copenhagen, Denmark: World Health Organization Regional Office for Europe (WHO/Europe).	2008-2012
Taiwan (Province of China)	Ministry of Health and Welfare (Taiwan). Taiwan National Health Insurance Claims Data 2016.	2016-2016
Turkey	Ministry of Health (Turkey). Turkey Diagnosis-Related Group Hospital Inpatient Database 2011-2012.	2008-2012
United States of America	National Center for Health Statistics (NCHS), Centers for Disease Control and Prevention (CDC). United States National Hospital Discharge Survey 1988-1992.	1988-1992
United States of America	National Center for Health Statistics (NCHS), Centers for Disease Control and Prevention (CDC). United States National Hospital Discharge Survey 1993-1997.	1993-1997
United States of America	National Center for Health Statistics (NCHS), Centers for Disease Control and Prevention (CDC). United States National Hospital Discharge Survey 1998-2002.	1998-2002
United States of America	Truven Health Analytics. United States MarketScan Claims and Medicare Data - 2000. Ann Arbor, United States: Truven Health Analytics.	2000-2000
United States of America	National Center for Health Statistics (NCHS), Centers for Disease Control and Prevention (CDC). United States National Hospital Discharge Survey 2003-2007.	2003-2007
United States of America	Healthcare Cost and Utilization Project (HCUP), Agency for Healthcare Research and Quality (AHRQ). United States State Inpatient Databases 2003-2007.	2003-2007

United States of America	National Center for Health Statistics (NCHS), Centers for Disease Control and Prevention (CDC). United States National Hospital Discharge Survey 2008-2010.	2008-2012
United States of America	Healthcare Cost and Utilization Project (HCUP), Agency for Healthcare Research and Quality (AHRQ). United States State Inpatient Databases 2008-2009.	2008-2012
United States of America	Truven Health Analytics. United States MarketScan Claims and Medicare Data - 2010. Ann Arbor, United States: Truven Health Analytics.	2010-2010
United States of America	Truven Health Analytics. United States MarketScan Claims and Medicare Data 2011. Ann Arbor, United States: Truven Health Analytics.	2011-2011
United States of America	Truven Health Analytics. United States MarketScan Claims and Medicare Data - 2012. Ann Arbor, United States: Truven Health Analytics.	2012-2012
United States of America	Truven Health Analytics. United States MarketScan Claims and Medicare Data 2013. Ann Arbor, United States: Truven Health Analytics.	2013-2013
United States of America	Truven Health Analytics. United States MarketScan Claims and Medicare Data 2014. Ann Arbor, United States: Truven Health Analytics.	2014-2014
United States of America	Truven Health Analytics. United States MarketScan Claims and Medicare Data 2015. Ann Arbor, United States: Truven Health Analytics.	2015-2015
United States of America	Truven Health Analytics. United States MarketScan Claims and Medicare Data 2016. Ann Arbor, United States: Truven Health Analytics.	2016-2016
Viet Nam	Ministry of Health (Vietnam). Vietnam Hospital Data 2013.	2013-2017

B) Data sources included

Country name	Data source	Year range
Austria	Federal Ministry of Health (Austria), Statistics Austria. Austria Hospital Inpatient Discharges 2003-2007.	2003-2007
Austria	Federal Ministry of Health (Austria), Statistics Austria. Austria Hospital Inpatient Discharges 2008-2012.	2008-2012
Austria	Federal Ministry of Health (Austria), Statistics Austria. Austria Hospital Inpatient Discharges 2013-2014.	2013-2017

Belgium	World Health Organization Regional Office for Europe (WHO/Europe). European Hospital Morbidity Database, 2008. Copenhagen, Denmark: World Health Organization Regional Office for Europe (WHO/Europe).	2003-2007
Belgium	World Health Organization Regional Office for Europe (WHO/Europe). European Hospital Morbidity Database, 2008. Copenhagen, Denmark: World Health Organization Regional Office for Europe (WHO/Europe).	2008-2012
Botswana	Ministry of Health (Botswana). Botswana Health Management Data System 2008-2009.	2003-2007
Botswana	Ministry of Health (Botswana). Botswana Health Management Data System 2008-2009.	2008-2012
Brazil	Ministry of Health (Brazil). Brazil Hospital Information System 1997. Rio de Janeiro, Brazil: Ministry of Health (Brazil).	1993-1997
Brazil	Ministry of Health (Brazil). Brazil Hospital Information System 1998-2002.	1998-2002
Brazil	Ministry of Health (Brazil). Brazil Hospital Information System 2003-2007.	2003-2007
Brazil	Ministry of Health (Brazil). Brazil Hospital Information System 2008-2012.	2008-2012
Brazil	Ministry of Health (Brazil). Brazil Hospital Information System 2013-2014.	2013-2017
Chile	Ministry of Health (Chile). Chile Hospital Discharges 2008-2012.	2008-2012
China	Center for Health Statistics and Information, National Health and Family Planning Commission (China), Shanghai Municipal Center for Disease Control and Prevention (Shanghai CDC), Shanghai Health Information Center. China Hospital Inpatient Data 2013-2016.	2013-2017
Croatia	World Health Organization Regional Office for Europe (WHO/Europe). European Hospital Morbidity Database, 2008. Copenhagen, Denmark: World Health Organization Regional Office for Europe (WHO/Europe).	1998-2002
Croatia	World Health Organization Regional Office for Europe (WHO/Europe). European Hospital Morbidity Database, 2008. Copenhagen, Denmark: World Health Organization Regional Office for Europe (WHO/Europe).	2003-2007
Croatia	World Health Organization Regional Office for Europe (WHO/Europe). European Hospital Morbidity Database, 2008. Copenhagen, Denmark: World Health Organization Regional Office for Europe (WHO/Europe).	2008-2012

Croatia	World Health Organization Regional Office for Europe (WHO/Europe). European Hospital Morbidity Database, 2008. Copenhagen, Denmark: World Health Organization Regional Office for Europe (WHO/Europe).	2013-2017
Cyprus	World Health Organization Regional Office for Europe (WHO/Europe). European Hospital Morbidity Database, 2008. Copenhagen, Denmark: World Health Organization Regional Office for Europe (WHO/Europe).	2003-2007
Cyprus	World Health Organization Regional Office for Europe (WHO/Europe). European Hospital Morbidity Database, 2008. Copenhagen, Denmark: World Health Organization Regional Office for Europe (WHO/Europe).	2008-2012
Czechia	World Health Organization Regional Office for Europe (WHO/Europe). European Hospital Morbidity Database, 2008. Copenhagen, Denmark: World Health Organization Regional Office for Europe (WHO/Europe).	2008-2012
Denmark	World Health Organization Regional Office for Europe (WHO/Europe). European Hospital Morbidity Database, 2008. Copenhagen, Denmark: World Health Organization Regional Office for Europe (WHO/Europe).	2003-2007
Ecuador	National Institute of Statistics and Censuses (Ecuador). Ecuador Hospital Inpatient Discharges 2013-2014.	2013-2017
England	NHS England. United Kingdom - England Hospital Episode Statistics 2001-2002.	1998-2002
England	NHS England. United Kingdom - England Hospital Episode Statistics 2003-2007.	2003-2007
England	NHS England. United Kingdom - England Hospital Episode Statistics 2008-2012.	2008-2012
England	NHS England. United Kingdom - England Hospital Episode Statistics 2013-2014.	2013-2017
Finland	World Health Organization Regional Office for Europe (WHO/Europe). European Hospital Morbidity Database, 2008. Copenhagen, Denmark: World Health Organization Regional Office for Europe (WHO/Europe).	1998-2002
Finland	World Health Organization Regional Office for Europe (WHO/Europe). European Hospital Morbidity Database, 2008. Copenhagen, Denmark: World Health Organization Regional Office for Europe (WHO/Europe).	2003-2007
Finland	World Health Organization Regional Office for Europe (WHO/Europe). European Hospital Morbidity Database, 2008. Copenhagen, Denmark: World Health Organization Regional Office for Europe (WHO/Europe).	2008-2012

Georgia	National Center for Disease Control and Public Health (Georgia). Georgia Hospital Data 2013-2014.	2013-2017
Georgia	National Center for Disease Control and Public Health (Georgia). Georgia Hospital Discharges 2016-2017.	2016-2017
Germany	Federal Statistical Office (Germany). Germany Hospital Discharges by Diagnosis 2009. Wiesbaden, Germany: Federal Statistical Office (Germany), 2011.	2008-2012
Iceland	World Health Organization Regional Office for Europe (WHO/Europe). European Hospital Morbidity Database, 2008. Copenhagen, Denmark: World Health Organization Regional Office for Europe (WHO/Europe).	2008-2012
India	Nazareth Hospital, Shillong, JSS Hospital, Mysore, King George's Medical University (India). India Hospital Inpatient Data 2014-2017.	2013-2017
India	St. John's Medical College Hospital (India). India - Bangalore St. John's Medical College Hospital Inpatient Data 2017.	2013-2017
Indonesia	Ministry of Health (Indonesia). Indonesia Integrated Hospital Data 2013. Jakarta, Indonesia: Ministry of Health (Indonesia), 2014.	2013-2017
Italy	Ministry of Health (Italy). Italy Hospital Inpatient Discharges 2005-2007.	2003-2007
Italy	Ministry of Health (Italy). Italy Hospital Inpatient Discharges 2008-2012.	2008-2012
Italy	Ministry of Health (Italy). Italy Hospital Inpatient Discharges 2013-2016.	2013-2017
Japan	Ministry of Health, Labour and Welfare (Japan). Japan Diagnosis Procedure Combination Database 2010-2012.	2008-2012
Japan	Ministry of Health, Labour and Welfare (Japan). Japan Diagnosis Procedure Combination Database 2013-2015.	2013-2017
Jordan	Ministry of Health (Jordan). Jordan Al-Bashir Hospital Discharges 2016.	2013-2017
Kenya	Ministry of Health (Kenya). Kenya National Inpatient Morbidity and Mortality Statistics 1999.	1998-2002
Kyrgyzstan	Mandatory Health Insurance Fund (Kyrgyzstan). Kyrgyzstan - Bishkek Clinical-Related Groups Hospital Claims 2012.	2008-2012
Latvia	World Health Organization Regional Office for Europe (WHO/Europe). European Hospital Morbidity Database, 2008. Copenhagen, Denmark: World Health Organization Regional Office for Europe (WHO/Europe).	2008-2012

Lithuania	World Health Organization Regional Office for Europe (WHO/Europe). European Hospital Morbidity Database, 2008. Copenhagen, Denmark: World Health Organization Regional Office for Europe (WHO/Europe).	1998-2002
Lithuania	World Health Organization Regional Office for Europe (WHO/Europe). European Hospital Morbidity Database, 2008. Copenhagen, Denmark: World Health Organization Regional Office for Europe (WHO/Europe).	2008-2012
Luxembourg	World Health Organization Regional Office for Europe (WHO/Europe). European Hospital Morbidity Database, 2008. Copenhagen, Denmark: World Health Organization Regional Office for Europe (WHO/Europe).	2008-2012
Malta	World Health Organization Regional Office for Europe (WHO/Europe). European Hospital Morbidity Database, 2008. Copenhagen, Denmark: World Health Organization Regional Office for Europe (WHO/Europe).	2003-2007
Malta	World Health Organization Regional Office for Europe (WHO/Europe). European Hospital Morbidity Database, 2008. Copenhagen, Denmark: World Health Organization Regional Office for Europe (WHO/Europe).	2008-2012
Mexico	Ministry of Health (Mexico). Mexico Ministry of Health Hospital Discharges 2000-2002.	1998-2002
Mexico	Ministry of Health (Mexico). Mexico Ministry of Health Hospital Discharges 2003-2007.	2003-2007
Mexico	Ministry of Health (Mexico). Mexico Ministry of Health Hospital Discharges 2008-2012.	2008-2012
Mexico	Ministry of Health (Mexico). Mexico Ministry of Health Hospital Discharges 2013-2015	2013-2017
Nepal	Department of Health Services, Ministry of Health and Population (Nepal). Nepal Hospital Inpatient Discharges 2010-2012.	2008-2012
Nepal	Department of Health Services, Ministry of Health and Population (Nepal). Nepal Hospital Inpatient Discharges 2013-2014.	2013-2017
New Zealand	Ministry of Health (New Zealand). New Zealand National Minimum Dataset 2000-2002.	1998-2002
New Zealand	Ministry of Health (New Zealand). New Zealand National Minimum Dataset 2003-2007.	2003-2007

New Zealand	Ministry of Health (New Zealand). New Zealand National Minimum Dataset 2008-2012.	2008-2012
New Zealand	Ministry of Health (New Zealand). New Zealand National Minimum Dataset 2013-2014.	2013-2017
New Zealand	Ministry of Health (New Zealand). New Zealand National Minimum Dataset 2016-2017.	2016-2017
Norway	Norwegian Directorate of Health. Norway Patient Register 2008-2012.	2008-2012
Philippines	Philippine Health Insurance Corporation. Philippine Health Insurance Corporation Claims 2013-2016.	2013-2017
Poland	World Health Organization Regional Office for Europe (WHO/Europe). European Hospital Morbidity Database, 2008. Copenhagen, Denmark: World Health Organization Regional Office for Europe (WHO/Europe).	2003-2007
Poland	World Health Organization Regional Office for Europe (WHO/Europe). European Hospital Morbidity Database, 2008. Copenhagen, Denmark: World Health Organization Regional Office for Europe (WHO/Europe).	2008-2012
Poland	National Health Fund (Poland). Poland National Health Fund Patient Claims 2015.	2015-2015
Poland	National Health Fund (Poland). Poland National Health Fund Patient Claims 2016.	2016-2016
Poland	National Health Fund (Poland). Poland National Health Fund Patient Claims 2017.	2017-2017
Romania	World Health Organization Regional Office for Europe (WHO/Europe). European Hospital Morbidity Database, 2008. Copenhagen, Denmark: World Health Organization Regional Office for Europe (WHO/Europe).	2008-2012
Serbia	World Health Organization Regional Office for Europe (WHO/Europe). European Hospital Morbidity Database, 2008. Copenhagen, Denmark: World Health Organization Regional Office for Europe (WHO/Europe).	2008-2012
Slovakia	World Health Organization Regional Office for Europe (WHO/Europe). European Hospital Morbidity Database, 2008. Copenhagen, Denmark: World Health Organization Regional Office for Europe (WHO/Europe).	1998-2002

Slovakia	World Health Organization Regional Office for Europe (WHO/Europe). European Hospital Morbidity Database, 2008. Copenhagen, Denmark: World Health Organization Regional Office for Europe (WHO/Europe).	2003-2007
Slovakia	World Health Organization Regional Office for Europe (WHO/Europe). European Hospital Morbidity Database, 2008. Copenhagen, Denmark: World Health Organization Regional Office for Europe (WHO/Europe).	2008-2012
Slovenia	World Health Organization Regional Office for Europe (WHO/Europe). European Hospital Morbidity Database, 2008. Copenhagen, Denmark: World Health Organization Regional Office for Europe (WHO/Europe).	2003-2007
Slovenia	World Health Organization Regional Office for Europe (WHO/Europe). European Hospital Morbidity Database, 2008. Copenhagen, Denmark: World Health Organization Regional Office for Europe (WHO/Europe).	2008-2012
Sweden	National Board of Health and Welfare (Sweden). Sweden National Patient Register 1998-2002.	1998-2002
Sweden	National Board of Health and Welfare (Sweden). Sweden National Patient Register 2003-2007.	2003-2007
Sweden	National Board of Health and Welfare (Sweden). Sweden National Patient Register 2008-2012.	2008-2012
Sweden	National Board of Health and Welfare (Sweden). Sweden National Patient Register 2013-2016.	2013-2017
Switzerland	World Health Organization Regional Office for Europe (WHO/Europe). European Hospital Morbidity Database, 2008. Copenhagen, Denmark: World Health Organization Regional Office for Europe (WHO/Europe).	1998-2002
Switzerland	World Health Organization Regional Office for Europe (WHO/Europe). European Hospital Morbidity Database, 2008. Copenhagen, Denmark: World Health Organization Regional Office for Europe (WHO/Europe).	2003-2007
Switzerland	World Health Organization Regional Office for Europe (WHO/Europe). European Hospital Morbidity Database, 2008. Copenhagen, Denmark: World Health Organization Regional Office for Europe (WHO/Europe).	2008-2012
Taiwan (Province of China)	Ministry of Health and Welfare (Taiwan). Taiwan National Health Insurance Claims Data 2016.	2016-2016

United States of America	National Center for Health Statistics (NCHS), Centers for Disease Control and Prevention (CDC). United States National Hospital Discharge Survey 1993-1997.	1993-1997
United States of America	National Center for Health Statistics (NCHS), Centers for Disease Control and Prevention (CDC). United States National Hospital Discharge Survey 1998-2002.	1998-2002
United States of America	Truven Health Analytics. United States MarketScan Claims and Medicare Data - 2000. Ann Arbor, United States: Truven Health Analytics.	2000-2000
United States of America	National Center for Health Statistics (NCHS), Centers for Disease Control and Prevention (CDC). United States National Hospital Discharge Survey 2003-2007.	2003-2007
United States of America	Healthcare Cost and Utilization Project (HCUP), Agency for Healthcare Research and Quality (AHRQ). United States State Inpatient Databases 2003-2007.	2003-2007
United States of America	National Center for Health Statistics (NCHS), Centers for Disease Control and Prevention (CDC). United States National Hospital Discharge Survey 2008-2010.	2008-2012
United States of America	Healthcare Cost and Utilization Project (HCUP), Agency for Healthcare Research and Quality (AHRQ). United States State Inpatient Databases 2008-2009.	2008-2012
United States of America	Truven Health Analytics. United States MarketScan Claims and Medicare Data - 2010. Ann Arbor, United States: Truven Health Analytics.	2010-2010
United States of America	Truven Health Analytics. United States MarketScan Claims and Medicare Data 2011. Ann Arbor, United States: Truven Health Analytics.	2011-2011
United States of America	Truven Health Analytics. United States MarketScan Claims and Medicare Data - 2012. Ann Arbor, United States: Truven Health Analytics.	2012-2012
United States of America	Truven Health Analytics. United States MarketScan Claims and Medicare Data 2013. Ann Arbor, United States: Truven Health Analytics.	2013-2013
United States of America	Truven Health Analytics. United States MarketScan Claims and Medicare Data 2014. Ann Arbor, United States: Truven Health Analytics.	2014-2014

United States of America	Truven Health Analytics. United States MarketScan Claims and Medicare Data 2015. Ann Arbor, United States: Truven Health Analytics.	2015-2015
United States of America	Truven Health Analytics. United States MarketScan Claims and Medicare Data 2016. Ann Arbor, United States: Truven Health Analytics.	2016-2016
Viet Nam	Ministry of Health (Vietnam). Vietnam Hospital Data 2013.	2013-2017

Table 4. Percentage change of the number of prevalent cases of BPH and the age-standardized prevalence of BPH, globally, between 2000 and 2019, by age-group

Age group	Percentage change between 2000 and 2019 (%)	
	**Cases (95% UI)**	**Age-standardized prevalence (95% UI)**
40 to 44	22·6 (16·7–26·8)	-7·02 (-11·5–-3·87)
45 to 49	36·6 (33·0–39·1)	-3·57 (-6·07–-1·80)
50 to 54	57·2 (54·2–59·3)	-4·17 (-6·00–-2·87)
55 to 59	77·7 (75·4–79·7)	0·0147 (-1·29–1·13)
60 to 64	61·1 (59·2–62·8)	-3·33 (-4·44–-2·29)
65 to 69	65·6 (64·0–67·5)	-1·84 (-2·81–-0·732)
70 to 74	55·8 (53·7–57·9)	-3·19 (-4·48–-1·88)
75 to 79	74·6 (72·8–76·3)	1·16 (0·112–2·16)
80 to 84	126 (124–129)	4·61 (3·41–5·69)
85 to 89	142 (138–145)	5·04 (3·29–6·49)
90 to 94	166 (160–170)	4·90 (2·70–6·71)
95 plus	173 (166–179)	0·166 (-2·27–2·45)

Table 5. Global, super-region, and country-level DALYs of benign prostatic hyperplasia and percentage change between 2000 and 2019

	2000		2019		Percentage change between 2000 and 2019	
	**Cases (95% UI)**	**Age-standardised DALYs per 100,000 (95% UI)**	**Cases (95% UI)**	**Age-standardised DALYs per 100,000 (95% UI)**	**Cases (95% UI)**	**Age-standardised DALYs (95% UI)**
**Global**	**1090000 (657000–1630000)**	**49·2 (29·9–73·2)**	**1860000 (1130000–2780000)**	**48·9 (29·7–72·6)**	**70·5 (68·6–72·7)**	**-0·640 (-1·46–0·249)**
**Central Europe, eastern Europe, and central Asia**	**194000 (117000–287000)**	**95·8 (58·3–140)**	**241000 (145000–361000)**	**94·7 (57·3–140)**	**24·6 (20·9–28·3)**	**-1·19 (-3·75–1·29)**
Central Asia	10300 (6080–15400)	50·9 (30·2–75·8)	15200 (8920–23000)	51·7 (30·5–77·5)	48·0 (39·2–56·9)	1·54 (-2·05–4·74)
Armenia	710 (408–1090)	50·6 (29·4–76·8)	928 (533–1420)	51·9 (29·7–78·6)	30·7 (19·5–42·7)	2·62 (-4·91–11·0)
Azerbaijan	1180 (677–1810)	50·7 (29·3–76·8)	2030 (1140–3090)	51·8 (29·9–78·3)	72·3 (56·5–90·0)	2·18 (-4·76–9·77)
Georgia	1560 (957–2290)	59·4 (36·9–87·6)	1470 (916–2160)	60·4 (37·3–88·9)	-5·30 (-12·0–1·97)	1·59 (-5·74–9·28)
Kazakhstan	2340 (1360–3580)	49·7 (29·4–75·6)	3360 (1920–5170)	50·8 (29·5–77·5)	43·6 (31·2–57·9)	2·20 (-5·90–10·8)
Kyrgyzstan	543 (317–829)	42·6 (25·0–65·3)	781 (454–1190)	43·4 (25·1–65·7)	43·8 (28·2–61·4)	1·86 (-6·00–11·0)
Mongolia	226 (130–339)	49·4 (29·1–75·0)	446 (258–677)	50·6 (29·7–77·2)	97·7 (78·9–116)	2·31 (-5·95–10·7)
Tajikistan	656 (384–1010)	50·2 (29·4–77·3)	1090 (628–1690)	51·1 (29·4–77·2)	66·4 (48·1–87·2)	1·69 (-5·82–10·6)
Turkmenistan	449 (259–670)	50·4 (29·0–76·0)	800 (464–1220)	51·4 (30·2–78·2)	78·0 (62·6–95·0)	1·92 (-5·97–11·3)

Uzbekistan	2600 (1500–3990)	50·3 (29·5–75·9)	4280 (2410–6660)	51·6 (29·9–79·8)	64·3 (47·6–84·4)	2·58 (-4·87–11·1)
Central Europe	44100 (27300–64600)	62·3 (38·3–91·0)	58400 (36400–85700)	61·8 (38·6–90·2)	32·5 (29·2–36·2)	-0·678 (-2·82–1·43)
Albania	754 (435–1160)	59·4 (34·4–90·1)	1280 (750–1950)	59·8 (35·1–90·7)	69·6 (58·0–84·0)	0·524 (-5·63–8·32)
Bosnia and Herzegovina	1290 (758–1960)	60·4 (35·5–91·6)	1650 (956–2540)	60·7 (35·5–92·1)	28·1 (16·5–40·6)	0·500 (-7·73–9·35)
Bulgaria	3680 (2180–5640)	60·8 (36·0–92·7)	3880 (2230–5900)	60·5 (35·0–91·9)	5·18 (-3·59–13·0)	-0·408 (-8·11–6·81)
Croatia	1780 (1100–2630)	58·6 (36·9–86·5)	2270 (1420–3300)	58·7 (36·7–85·1)	27·6 (18·5–38·5)	0·157 (-6·91–8·18)
Czechia	4970 (2970–7470)	77·9 (46·6–118)	7430 (4430–11000)	78·0 (46·5–116)	49·4 (37·1–61·5)	0·0938 (-8·02–7·63)
Hungary	3850 (2260–5950)	60·2 (35·4–92·1)	4810 (2800–7330)	60·3 (35·3–91·0)	24·9 (14·8–36·7)	0·0426 (-7·81–8·58)
Montenegro	206 (121–313)	60·7 (35·6–91·6)	273 (160–415)	60·4 (35·4–90·9)	32·5 (22·4–42·7)	-0·555 (-7·71–6·76)
North Macedonia	644 (376–982)	61·0 (36·0–93·0)	955 (556–1450)	61·1 (35·6–91·8)	48·4 (37·0–61·7)	0·115 (-7·21–8·58)
Poland	10200 (6300–15000)	51·0 (31·2–74·7)	15300 (9380–22400)	51·1 (31·6–74·8)	49·8 (45·2–54·0)	0·324 (-1·99–2·45)
Romania	10000 (6180–14900)	71·8 (44·6–106)	11700 (7300–17400)	72·4 (45·2–108)	17·1 (8·29–26·0)	0·817 (-6·48–7·69)
Serbia	4360 (2700–6410)	69·8 (43·2–103)	5330 (3350–7950)	69·9 (43·8–104)	22·4 (13·5–32·8)	0·257 (-6·93–7·76)
Slovakia	1800 (1130–2660)	65·9 (41·4–96·7)	2670 (1670–3920)	65·8 (41·3–96·5)	47·8 (37·5–58·4)	-0·215 (-6·98–6·86)

Slovenia	558 (347–816)	46·9 (29·3–68·6)	898 (559–1310)	46·5 (29·0–67·6)	60·9 (48·0–73·0)	-0·890 (-8·99–6·40)
Eastern Europe	139000 (83500–208000)	127 (76·3–188)	168000 (99100–253000)	128 (76·5–190)	20·3 (15·4–25·3)	0·934 (-2·60–4·32)
Belarus	6430 (3830–9620)	123 (73·3–183)	7280 (4300–11000)	123 (74·1–183)	13·2 (5·33–20·6)	0·265 (-6·55–6·76)
Estonia	977 (587–1470)	124 (75·2–185)	1250 (759–1850)	125 (76·3–185)	28·2 (20·0–36·0)	1·17 (-4·98–7·11)
Latvia	1620 (991–2360)	123 (76·4–179)	1840 (1150–2710)	126 (78·1–185)	13·9 (5·48–20·9)	2·04 (-5·12–8·03)
Lithuania	2550 (1600–3710)	135 (85·2–196)	2920 (1830–4270)	136 (85·3–200)	14·6 (7·61–21·2)	1·12 (-5·14–6·77)
Republic of Moldova	2350 (1410–3500)	124 (74·6–185)	2920 (1740–4400)	125 (74·4–185)	24·4 (17·0–32·3)	0·319 (-5·54–6·35)
Russia	89900 (54100–135000)	127 (76·4–189)	114000 (67500–174000)	128 (76·4–192)	27·3 (19·9–34·6)	1·16 (-4·42–6·54)
Ukraine	35400 (20900–53500)	127 (75·8–190)	36800 (21700–54800)	128 (76·2–190)	4·02 (-2·23–10·6)	0·438 (-5·05–5·92)
**High-income**	**233000 (144000–340000)**	**37·3 (23·1–54·2)**	**347000 (213000–506000)**	**36·5 (22·5–52·9)**	**48·8 (46·3–51·6)**	**-2·29 (-3·74–-0·833)**
Australasia	5340 (3150–8260)	39·0 (23·0–60·4)	9360 (5480–14400)	39·1 (22·9–60·2)	75·2 (61·8–89·9)	0·307 (-7·19–8·78)
Australia	4380 (2480–6870)	38·1 (21·6–59·8)	7710 (4440–12100)	38·3 (22·1–60·2)	76·2 (60·1–94·6)	0·601 (-8·49–11·0)
New Zealand	962 (587–1430)	43·7 (26·8–65·0)	1640 (1010–2420)	43·4 (26·8–63·6)	70·9 (56·0–85·8)	-0·888 (-9·32–7·48)
High income Asia Pacific	30600 (18100–47000)	24·7 (14·6–37·7)	47700 (28000–73000)	23·8 (14·0–36·2)	55·7 (48·2–65·1)	-3·40 (-6·29–-0·640)

Brunei	13·1 (7·51–20·6)	24·1 (13·9–37·8)	30·6 (17·6–48·5)	23·3 (13·5–35·8)	134 (109–164)	-3·51 (-13·2–7·61)
Japan	26400 (15600–40300)	25·1 (14·9–38·2)	37400 (22200–57300)	24·4 (14·4–37·0)	41·6 (34·2–50·9)	-2·62 (-4·92–-0·314)
South Korea	3880 (2190–6200)	22·4 (12·9–35·6)	9400 (5380–14800)	22·2 (12·8–34·6)	142 (114–171)	-1·06 (-11·4–9·97)
Singapore	317 (179–492)	22·6 (13·1–35·1)	861 (492–1350)	22·0 (12·6–34·3)	172 (144–199)	-2·44 (-11·9–6·87)
High income North America	60300 (38500–88700)	34·3 (21·8–50·4)	101000 (64300–148000)	34·9 (22·4–51·4)	66·9 (63·2–70·8)	1·90 (-0·0970–3·84)
Canada	6370 (3720–9750)	35·1 (20·5–53·4)	11300 (6570–17500)	35·3 (20·6–54·6)	77·3 (62·2–93·8)	0·594 (-7·56–9·52)
Greenland	7·26 (4·12–11·3)	34·2 (19·8–51·8)	13·1 (7·39–20·4)	34·5 (20·1–52·2)	81·0 (63·1–99·8)	0·689 (-7·75–9·44)
USA	54000 (34000–79800)	34·2 (21·5–50·4)	89400 (56800–132000)	34·9 (22·1–51·6)	65·7 (62·1–69·4)	2·03 (0·235–3·96)
Southern Latin America	6080 (3540–9360)	24·9 (14·5–38·5)	9210 (5310–14400)	24·8 (14·4–38·6)	51·4 (41·2–63·3)	-0·246 (-6·86–7·23)
Argentina	3950 (2310–6140)	23·7 (13·8–36·8)	5550 (3190–8700)	23·3 (13·4–36·4)	40·5 (27·9–55·4)	-1·42 (-10·2–8·53)
Chile	1700 (974–2630)	28·9 (16·5–44·5)	3140 (1760–4880)	28·4 (16·0–44·1)	85·0 (66·8–106)	-1·64 (-11·5–8·65)
Uruguay	434 (251–679)	23·1 (13·3–36·3)	520 (302–801)	22·9 (13·3–35·3)	19·7 (8·67–31·7)	-0·873 (-10·3–8·84)
Western Europe	131000 (80200–191000)	45·3 (27·7–66·0)	180000 (110000–263000)	44·9 (27·4–65·6)	37·7 (34·1–41·2)	-0·798 (-3·10–1·52)
Andorra	14·2 (8·19–22·1)	32·7 (18·9–50·6)	22·8 (13·3–35·3)	32·3 (18·7–50·2)	60·2 (43·7–76·2)	-1·39 (-11·4–8·20)

Austria	4860 (3040–7100)	90·1 (56·4–132)	7190 (4430–10600)	91·0 (55·9–134)	48·1 (38·1–58·2)	0·929 (-6·05–7·70)
Belgium	5690 (2980–8930)	74·5 (38·9–116)	7310 (3830–11300)	71·5 (37·4–110)	28·5 (6·53–44·2)	-3·99 (-19·8–7·39)
Cyprus	158 (96·7–241)	32·4 (20·0–49·3)	307 (186–464)	31·6 (19·3–47·8)	95·0 (77·9–114)	-2·27 (-10·8–7·30)
Denmark	1300 (718–2030)	34·7 (19·2–54·3)	1890 (1060–3040)	34·5 (19·3–55·2)	46·0 (31·4–62·2)	-0·418 (-9·52–10·2)
Finland	2710 (1690–3980)	78·6 (49·2–116)	4530 (2830–6670)	78·8 (49·2–116)	67·6 (56·3–80·0)	0·230 (-6·74–7·64)
France	13400 (7710–20600)	32·6 (18·7–50·6)	19100 (11100–29900)	32·4 (18·6–50·4)	42·3 (30·2–56·8)	-0·777 (-9·15–8·81)
Germany	20100 (11500–31100)	32·5 (18·5–50·3)	27300 (15500–42100)	32·3 (18·3–49·8)	36·2 (23·2–51·8)	-0·725 (-9·24–8·83)
Greece	2790 (1590–4380)	32·4 (18·4–50·4)	3290 (1860–5090)	32·1 (18·4–49·4)	18·1 (6·71–31·4)	-1·09 (-10·6–8·70)
Iceland	49·1 (27·6–75·8)	31·2 (17·6–47·6)	82·0 (47·0–128)	30·8 (17·7–48·0)	66·9 (49·6–84·6)	-1·19 (-11·0–8·69)
Ireland	695 (398–1070)	33·0 (19·0–50·8)	1170 (677–1830)	32·5 (18·7–50·9)	68·7 (53·2–84·9)	-1·50 (-10·4–7·75)
Israel	972 (556–1510)	33·6 (19·2–52·1)	1770 (1010–2770)	33·2 (19·0–51·9)	81·8 (65·5–99·8)	-1·18 (-9·75–8·56)
Italy	30800 (19100–45600)	67·9 (42·1–101)	39700 (24400–58600)	67·7 (42·0–101)	28·9 (25·5–32·6)	-0·277 (-2·65–2·29)
Luxembourg	88·7 (51·0–139)	32·6 (18·9–51·2)	150 (86·7–235)	32·2 (18·7–50·4)	69·0 (53·1–85·2)	-1·14 (-9·97–8·15)
Malta	108 (65·0–165)	44·3 (26·7–67·6)	196 (119–297)	43·9 (26·6–66·5)	82·1 (67·4–99·4)	-0·974 (-8·94–7·96)

Monaco	11·4 (6·56–17·6)	33·1 (18·7–50·6)	14·2 (8·27–22·2)	32·7 (18·9–50·9)	24·5 (13·4–36·0)	-1·29 (-10·1–7·48)
Netherlands	3360 (1940–5260)	33·3 (19·2–52·0)	5370 (3130–8350)	33·0 (19·4–50·9)	59·6 (44·9–74·7)	-0·884 (-9·40–8·26)
Norway	3260 (1980–4860)	107 (64·0–159)	4880 (2970–7260)	107 (64·5–160)	49·8 (46·7–52·9)	0·126 (-1·56–1·96)
Portugal	2480 (1430–3830)	32·8 (19·0–50·3)	3350 (1950–5180)	32·7 (18·9–50·3)	35·1 (22·2–48·3)	-0·485 (-9·85–9·37)
San Marino	6·59 (3·86–10·1)	33·1 (19·0–51·1)	9·64 (5·53–15·1)	32·7 (18·6–51·5)	46·3 (33·2–59·5)	-1·11 (-9·55–8·03)
Spain	9870 (5620–15400)	32·9 (18·5–51·1)	13400 (7700–20800)	32·6 (18·6–51·0)	35·7 (24·3–49·8)	-0·796 (-9·39–9·42)
Sweden	2770 (1630–4290)	39·5 (22·9–61·0)	3860 (2260–5980)	39·6 (22·8–61·4)	39·3 (27·0–52·7)	0·243 (-8·22–9·30)
Switzerland	4920 (3030–7160)	97·4 (60·1–142)	7640 (4830–11100)	97·1 (60·9–142)	55·3 (45·2–65·7)	-0·400 (-7·08–6·46)
UK	20400 (12800–29900)	48·1 (30·0–70·0)	27500 (17200–39800)	47·3 (29·2–68·9)	34·6 (31·9–37·3)	-1·55 (-3·35–0·266)
**Latin America and Caribbean**	**80300 (48700–120000)**	**58·6 (35·7–87·4)**	**155000 (94500–231000)**	**59·1 (36·0–87·3)**	**93·5 (90·3–97·0)**	**0·751 (-0·954–2·44)**
Andean Latin America	10000 (5930–15300)	74·1 (43·8–113)	18800 (11300–28600)	71·9 (43·1–109)	87·8 (77·7–99·1)	-2·91 (-8·01–3·04)
Bolivia	1420 (818–2210)	68·3 (39·5–107)	2720 (1580–4280)	66·4 (38·8–104)	91·4 (75·8–109)	-2·79 (-10·8–6·19)
Ecuador	3390 (2000–5190)	91·4 (54·2–140)	6280 (3730–9770)	88·1 (52·7–137)	85·6 (71·3–102)	-3·61 (-10·9–4·98)
Peru	5230 (3000–8190)	67·3 (39·0–106)	9840 (5760–15200)	65·7 (38·5–102)	88·2 (73·8–106)	-2·35 (-9·88–6·77)

Caribbean	8620 (5030–13500)	56·0 (32·8–87·5)	13300 (7800–20600)	55·2 (32·2–85·1)	54·5 (47·6–60·9)	-1·59 (-6·07–2·42)
Antigua and Barbuda	14·6 (8·49–22·6)	57·5 (33·3–89·2)	26·9 (15·6–42·2)	56·4 (33·2–87·9)	83·9 (66·6–101)	-1·91 (-10·0–6·63)
Bahamas	48·3 (28·1–75·7)	57·5 (33·5–89·8)	94·3 (55·3–149)	56·3 (32·9–87·9)	95·3 (80·2–113)	-2·04 (-9·65–6·31)
Barbados	77·9 (45·0–121)	58·6 (34·0–92·8)	133 (76·3–206)	57·4 (33·1–89·0)	70·3 (55·0–89·8)	-2·08 (-10·4–7·64)
Belize	33·8 (19·8–53·0)	58·0 (33·9–90·8)	74·7 (44·1–116)	57·4 (33·6–89·3)	121 (101–143)	-0·959 (-9·22–8·09)
Bermuda	18·6 (10·7–28·8)	56·1 (32·5–87·2)	32·7 (19·0–51·8)	55·1 (31·9–87·2)	76·3 (61·3–94·5)	-1·76 (-9·65–7·76)
Cuba	3310 (1920–5200)	54·9 (31·9–86·4)	4800 (2790–7400)	53·8 (31·2–82·9)	45·3 (33·0–57·5)	-2·17 (-10·5–6·16)
Dominica	18·9 (11·1–30·0)	58·1 (34·0–91·8)	25·4 (15·1–39·4)	56·6 (33·8–87·2)	34·1 (22·9–46·3)	-2·55 (-9·75–5·41)
Dominican Republic	1380 (795–2140)	54·7 (31·5–85·2)	2320 (1350–3600)	54·3 (31·5–84·1)	68·5 (54·8–84·1)	-0·732 (-8·49–8·25)
Grenada	17·2 (10·0–26·9)	58·0 (34·0–90·2)	30·3 (17·4–47·0)	57·4 (33·5–88·5)	76·1 (61·9–93·0)	-1·06 (-8·24–7·26)
Guyana	106 (61·7–166)	58·7 (34·4–91·5)	158 (91·5–249)	57·3 (33·4–89·8)	49·6 (37·0–62·1)	-2·43 (-10·2–5·02)
Haiti	1060 (606–1670)	56·4 (32·5–87·9)	1660 (966–2630)	55·4 (32·2–87·0)	56·3 (43·5–70·1)	-1·89 (-9·64–6·14)
Jamaica	579 (334–907)	58·7 (33·8–92·1)	821 (479–1280)	58·4 (34·2–91·1)	41·8 (31·1–55·9)	-0·531 (-8·01–9·12)
Puerto Rico	1220 (716–1900)	58·5 (34·3–90·9)	1880 (1090–2900)	57·9 (33·7–89·6)	53·2 (40·5–66·5)	-1·17 (-9·60–7·66)

Saint Kitts and Nevis	9·49 (5·53–14·7)	57·3 (33·2–89·6)	17·5 (10·0–27·1)	56·3 (32·7–87·6)	84·6 (60·7–108)	-1·72 (-10·6–6·46)
Saint Lucia	30·3 (17·9–47·8)	57·9 (34·2–91·5)	56·3 (33·0–87·2)	56·1 (33·0–86·7)	86·0 (69·9–106)	-3·11 (-10·7–7·16)
Saint Vincent and the Grenadines	22·2 (13·0–35·0)	59·6 (35·0–93·9)	41·3 (24·4–64·4)	58·7 (34·4–91·0)	86·3 (70·0–105)	-1·49 (-9·91–7·63)
Suriname	85·3 (49·9–132)	56·8 (33·5–88·0)	151 (87·6–238)	56·9 (33·0–89·5)	77·3 (63·6–93·4)	0·222 (-7·14–8·73)
Trinidad and Tobago	272 (154–423)	55·6 (31·9–86·3)	499 (288–791)	54·5 (31·9–86·7)	83·6 (67·2–102)	-1·86 (-10·4–7·64)
Virgin Islands	30·1 (17·4–47·8)	57·3 (33·1–90·2)	51·1 (29·3–80·2)	56·4 (32·3–88·3)	69·4 (51·4–88·1)	-1·64 (-9·85–6·83)
Central Latin America	43000 (26200–63900)	81·0 (49·5–120)	86200 (52700–128000)	81·8 (50·2–121)	100 (95·6–105)	0·960 (-1·48–3·31)
Colombia	8590 (5010–13400)	73·0 (42·5–113)	17400 (10100–26900)	73·6 (42·8–113)	102 (85·6–121)	0·794 (-7·62–9·82)
Costa Rica	865 (499–1320)	74·1 (43·0–113)	1720 (1010–2660)	73·7 (43·1–113)	99·1 (82·5–116)	-0·584 (-9·06–7·69)
El Salvador	1260 (743–1950)	74·1 (43·7–115)	1860 (1080–2870)	74·8 (43·3–116)	47·8 (37·0–59·7)	1·02 (-6·41–9·25)
Guatemala	1900 (1110–2930)	74·8 (43·8–115)	3710 (2160–5680)	76·6 (44·7–117)	95·5 (79·9–113)	2·36 (-5·54–11·1)
Honduras	984 (577–1520)	74·2 (43·5–114)	2030 (1200–3130)	74·9 (44·5–115)	106 (89·4–125)	0·932 (-7·39–9·80)
Mexico	23500 (14700–34500)	88·0 (55·5–129)	46700 (29200–68400)	89·0 (55·9–131)	98·3 (94·7–102)	1·23 (-0·530–2·94)
Nicaragua	726 (429–1120)	73·7 (43·7–114)	1380 (816–2150)	74·7 (44·1–114)	90·6 (75·6–109)	1·35 (-6·28–10·5)

Panama	734 (427–1130)	74·3 (43·2–115)	1480 (877–2300)	74·9 (44·3–116)	102 (87·0–118)	0·880 (-6·65–8·72)
Venezuela	4430 (2590–6840)	75·0 (44·0–116)	10000 (5890–15300)	75·6 (44·9–116)	126 (108–146)	0·742 (-7·14–9·87)
Tropical Latin America	18600 (11500–27500)	33·7 (20·9–49·8)	36900 (22700–54700)	34·3 (21·0–50·3)	98·8 (93·1–105)	1·75 (-1·05–4·84)
Brazil	18100 (11200–26700)	33·6 (20·8–49·6)	36000 (22100–53200)	34·2 (20·9–50·1)	99·0 (93·1–105)	1·76 (-1·20–4·99)
Paraguay	519 (305–814)	38·0 (22·3–59·5)	989 (576–1550)	38·8 (22·8–60·8)	90·6 (73·5–108)	2·14 (-6·71–11·6)
**North Africa and Middle East**	**21600 (12500–33700)**	**19·5 (11·2–30·1)**	**40600 (23300–63400)**	**19·6 (11·4–30·3)**	**88·1 (80·9–95·7)**	**0·605 (-2·57–4·15)**
Afghanistan	772 (449–1220)	19·3 (11·4–30·1)	949 (537–1480)	19·6 (11·2–30·7)	22·9 (7·60–41·0)	1·60 (-9·53–12·5)
Algeria	1540 (889–2400)	19·4 (11·4–30·3)	3230 (1850–5060)	19·5 (11·2–30·0)	110 (89·8–132)	0·662 (-8·96–10·2)
Bahrain	24·8 (14·2–39·6)	21·2 (12·3–33·3)	127 (69·0–202)	21·0 (12·1–32·8)	410 (343–472)	-0·919 (-11·5–7·57)
Egypt	3530 (1990–5570)	19·4 (10·9–30·2)	6500 (3700–10300)	19·7 (11·2–30·8)	84·1 (66·2–105)	1·14 (-8·12–11·3)
Iran	3770 (2130–5930)	20·1 (11·5–31·3)	7050 (4060–11000)	20·4 (11·8–31·8)	87·1 (74·3–103)	1·42 (-3·67–8·20)
Iraq	978 (586–1520)	20·2 (12·0–31·4)	2120 (1210–3390)	20·5 (11·8–32·1)	117 (96·0–141)	1·62 (-7·54–11·8)
Jordan	218 (124–344)	23·2 (13·3–35·9)	731 (417–1140)	22·9 (12·9–35·6)	236 (202–275)	-1·22 (-9·85–8·83)
Kuwait	101 (58·5–158)	20·0 (11·6–31·0)	267 (152–425)	19·7 (11·4–30·9)	165 (134–199)	-1·41 (-11·1–9·26)

Lebanon	317 (182–506)	19·2 (11·0–30·2)	440 (258–695)	18·9 (11·0–29·5)	38·7 (24·0–53·8)	-1·87 (-11·8–8·73)
Libya	234 (132–364)	19·4 (11·1–30·3)	450 (260–700)	19·2 (11·2–29·8)	92·1 (70·2–115)	-1·16 (-11·2–9·37)
Morocco	1640 (950–2550)	19·4 (11·2–29·8)	2940 (1670–4640)	19·5 (11·1–29·8)	78·9 (60·3–101)	0·522 (-9·09–11·3)
Oman	83·9 (48·0–133)	19·9 (11·6–31·2)	179 (101–286)	20·4 (12·0–31·6)	113 (89·7–139)	2·54 (-6·63–12·7)
Palestine	96·5 (55·5–153)	19·4 (11·1–30·4)	208 (119–331)	19·6 (11·4–30·7)	116 (93·0–142)	1·36 (-8·26–12·0)
Qatar	24·1 (13·5–39·1)	21·2 (12·4–33·7)	144 (79·3–235)	20·9 (12·0–32·4)	497 (424–581)	-1·80 (-10·5–8·11)
Saudi Arabia	828 (472–1300)	19·1 (10·9–29·9)	1740 (986–2720)	19·0 (11·0–29·0)	110 (89·2–134)	-0·789 (-9·19–9·11)
Sudan	1140 (654–1790)	19·0 (10·9–29·6)	1780 (1030–2770)	19·0 (11·0–29·8)	56·1 (39·7–72·4)	0·358 (-9·81–9·74)
Syria	655 (372–1060)	19·0 (10·9–30·1)	1180 (669–1830)	18·8 (10·9–29·1)	79·4 (61·9–99·0)	-1·07 (-10·0–9·16)
Tunisia	660 (375–1050)	19·2 (11·0–30·3)	1190 (683–1880)	19·3 (11·2–30·0)	80·2 (61·8–101)	0·400 (-9·28–11·3)
Turkey	4280 (2420–6630)	19·2 (10·9–29·6)	7740 (4490–12100)	18·9 (11·0–29·6)	81·0 (61·3–102)	-1·18 (-11·1–10·1)
United Arab Emirates	78·1 (42·0–127)	20·0 (11·5–31·4)	462 (252–752)	19·9 (11·7–30·9)	491 (406–589)	-0·556 (-9·44–8·72)
Yemen	594 (339–949)	18·9 (11·0–29·6)	1130 (631–1780)	18·9 (10·7–29·1)	90·4 (71·4–113)	-0·0670 (-9·39–10·6)
**South Asia**	**218000 (129000–334000)**	**63·2 (37·1–95·7)**	**418000 (245000–641000)**	**63·6 (37·5–96·5)**	**91·5 (87·3–96·4)**	**0·654 (-0·932–2·40)**

Bangladesh	13700<br>(8020–21000)	46·0<br>(27·2–71·1)	31200<br>(18300–47300)	46·5<br>(27·1–70·5)	129<br>(108–148)	0·967<br>(-7·25–9·07)
Bhutan	73·7<br>(42·7–116)	45·9<br>(26·9–71·5)	130<br>(75·0–200)	47·1<br>(27·5–72·8)	76·2<br>(61·2–92·7)	2·62<br>(-5·59–12·0)
India	186000<br>(109000–285000)	67·7<br>(39·8–102)	356000<br>(209000–544000)	67·4<br>(39·7–102)	90·9<br>(86·4–96·1)	-0·383<br>(-2·05–1·38)
Nepal	2370<br>(1430–3450)	39·3<br>(23·9–57·1)	4320<br>(2610–6410)	40·6<br>(24·7–60·1)	82·7<br>(68·5–101)	3·46<br>(-4·48–12·6)
Pakistan	15800<br>(9170–24400)	48·7<br>(28·3–76·1)	26600<br>(15600–41100)	51·2<br>(30·0–79·7)	67·9<br>(58·0–77·8)	5·04<br>(-0·476–10·7)
**Southeast Asia, east Asia, and Oceania**	**317000<br>(190000–482000)**	**49·3<br>(29·3–74·6)**	**614000<br>(366000–927000)**	**50·2<br>(29·9–76·3)**	**93·8<br>(90·5–97·4)**	**1·88<br>(0·421–3·26)**
East Asia	217000<br>(128000–334000)	44·3<br>(26·2–67·4)	427000<br>(254000–656000)	45·0<br>(26·7–68·9)	97·3<br>(93·1–102)	1·59<br>(-0·183–3·27)
China	204000<br>(121000–316000)	43·3<br>(25·6–65·9)	406000<br>(241000–623000)	44·0<br>(26·1–67·5)	98·7<br>(94·3–103)	1·75<br>(0·00198–3·52)
North Korea	3810<br>(2150–5970)	60·0<br>(34·1–93·7)	6850<br>(4010–10500)	61·0<br>(35·4–94·0)	79·9<br>(62·5–96·1)	1·71<br>(-6·13–9·64)
Taiwan (province of China)	8510<br>(5010–12900)	77·3<br>(45·9–117)	14400<br>(8600–21800)	77·9<br>(46·3–118)	69·5<br>(56·1–82·6)	0·848<br>(-6·07–7·53)
Oceania	1020<br>(592–1560)	62·5<br>(36·1–95·5)	1920<br>(1120–2940)	64·4<br>(38·0–98·0)	89·0<br>(78·4–101)	2·98<br>(-2·57–8·50)
American Samoa	8·06<br>(4·69–12·3)	60·6<br>(35·0–91·8)	13·3<br>(7·84–20·1)	62·5<br>(37·3–95·6)	64·5<br>(50·4–77·3)	3·05<br>(-5·34–9·75)
Cook Islands	5·14<br>(2·97–7·90)	63·8<br>(37·0–97·2)	8·17<br>(4·74–12·3)	66·0<br>(38·2–99·8)	58·9<br>(45·3–71·0)	3·40<br>(-4·94–10·5)
Fiji	126<br>(73·3–193)	72·3<br>(42·7–110)	225<br>(131–343)	72·6<br>(42·6–109)	78·3<br>(63·5–93·5)	0·488<br>(-7·06–8·48)

Guam	29·7 (17·2–45·7)	60·8 (35·2–93·1)	55·4 (32·2–85·7)	62·8 (36·5–97·3)	86·4 (73·1–103)	3·24 (-3·76–11·6)
Kiribati	10·1 (5·84–15·3)	67·9 (39·6–104)	16·0 (9·35–24·4)	69·5 (40·9–105)	58·4 (44·4–73·6)	2·34 (-4·56–9·31)
Marshall Islands	4·58 (2·70–6·99)	61·0 (35·6–93·6)	9·15 (5·25–14·2)	63·0 (37·0–96·3)	99·6 (81·5–118)	3·33 (-4·55–11·0)
Micronesia (Federated States of)	12·9 (7·60–19·8)	63·5 (36·9–98·0)	17·8 (10·4–27·2)	65·8 (38·9–100)	38·4 (24·7–54·0)	3·57 (-3·67–11·2)
Nauru	0·843 (0·480–1·29)	62·9 (36·6–96·1)	0·795 (0·453–1·23)	65·4 (37·8–98·8)	-5·73 (-14·2–3·76)	3·90 (-3·26–11·7)
Niue	0·631 (0·368–0·969)	63·8 (37·0–96·9)	0·627 (0·361–0·950)	66·1 (38·4–100)	-0·704 (-8·39–7·83)	3·48 (-3·59–11·5)
Northern Mariana Islands	6·14 (3·61–9·21)	61·1 (35·6–92·4)	15·0 (8·68–22·8)	62·7 (36·8–96·4)	144 (121–169)	2·66 (-5·62–10·7)
Palau	3·73 (2·21–5·70)	63·7 (37·3–97·5)	6·08 (3·56–9·52)	65·9 (38·7–99·7)	63·1 (49·3–79·0)	3·34 (-3·89–12·4)
Papua New Guinea	626 (362–969)	61·1 (35·2–93·6)	1270 (726–1940)	63·2 (36·9–96·7)	103 (85·4–121)	3·44 (-4·89–11·4)
Samoa	29·1 (17·1–44·8)	62·1 (36·5–94·3)	40·3 (23·4–61·3)	63·9 (37·6–97·3)	38·4 (25·6–50·7)	2·87 (-5·51–10·3)
Solomon Islands	54·3 (31·2–83·9)	62·1 (36·1–95·3)	79·7 (46·6–121)	65·1 (38·4–99·0)	46·9 (35·8–61·9)	4·84 (-2·18–14·9)
Tokelau	0·341 (0·196–0·519)	62·9 (36·0–95·4)	0·428 (0·251–0·653)	65·5 (38·5–99·1)	25·3 (14·8–36·4)	4·04 (-3·61–12·5)
Tonga	19·3 (11·1–29·7)	64·2 (37·3–97·7)	23·2 (13·5–35·5)	67·0 (39·0–102)	20·3 (11·8–29·4)	4·30 (-2·54–12·2)
Tuvalu	2·06 (1·20–3·21)	63·0 (37·0–97·2)	2·88 (1·69–4·35)	65·1 (38·7–99·1)	39·8 (29·5–51·2)	3·40 (-4·03–11·0)

Vanuatu	23·0<br>(13·4–35·4)	58·8<br>(34·2–90·2)	51·1<br>(29·5–78·7)	62·1<br>(35·5–95·4)	122<br>(104–141)	5·55<br>(-2·44–14·2)
Southeast Asia	98900<br>(58300–148000)	68·7<br>(41·1–103)	184000<br>(108000–277000)	69·7<br>(41·8–104)	86·3<br>(81·3–91·7)	1·56<br>(-0·551–3·68)
Cambodia	1420<br>(831–2160)	62·9<br>(37·1–96·3)	2920<br>(1710–4430)	64·2<br>(37·7–97·4)	105<br>(89·8–125)	2·10<br>(-5·16–11·0)
Indonesia	39800<br>(23600–59900)	71·2<br>(42·7–107)	69700<br>(41200–105000)	72·9<br>(44·0–109)	75·1<br>(69·5–81·3)	2·41<br>(-0·714–5·34)
Laos	724<br>(422–1110)	64·1<br>(37·2–97·4)	1260<br>(721–1930)	65·8<br>(38·0–101)	73·6<br>(59·1–88·5)	2·66<br>(-5·04–10·9)
Malaysia	3720<br>(2140–5680)	64·8<br>(37·9–97·8)	8570<br>(4960–13200)	66·3<br>(38·4–102)	131<br>(114–152)	2·34<br>(-4·70–10·7)
Maldives	45·8<br>(26·7–70·4)	63·7<br>(37·5–97·3)	92·9<br>(53·7–141)	65·2<br>(37·9–99·4)	103<br>(83·9–124)	2·32<br>(-5·91–10·9)
Mauritius	263<br>(154–398)	66·3<br>(38·3–101)	585<br>(343–894)	72·5<br>(42·4–110)	122<br>(104–142)	9·39<br>(0·568–18·7)
Myanmar	7660<br>(4470–11600)	62·5<br>(36·6–95·8)	12200<br>(7100–18800)	64·7<br>(38·0–100)	59·5<br>(47·4–74·5)	3·45<br>(-3·94–12·5)
Philippines	10300<br>(5960–15500)	58·8<br>(34·3–89·1)	19700<br>(11400–29900)	59·6<br>(34·8–90·6)	91·5<br>(87·1–95·9)	1·23<br>(-0·344–2·90)
Seychelles	17·3<br>(10·2–26·6)	63·2<br>(37·4–96·6)	32·2<br>(18·7–49·3)	64·7<br>(37·6–99·4)	86·0<br>(68·5–105)	2·42<br>(-6·10–12·2)
Sri Lanka	4050<br>(2350–6090)	63·6<br>(37·4–95·9)	7620<br>(4400–11800)	66·1<br>(38·3–101)	88·4<br>(74·1–105)	3·93<br>(-3·41–12·6)
Thailand	13800<br>(7980–21200)	64·1<br>(37·5–98·2)	30000<br>(17600–45700)	64·2<br>(38·0–99·1)	118<br>(100–135)	0·191<br>(-7·61–8·10)
Timor-Leste	116<br>(67·3–177)	61·0<br>(35·7–92·9)	250<br>(145–383)	63·3<br>(36·7–96·5)	115<br>(96·8–137)	3·88<br>(-4·07–12·4)

Vietnam	16900 (10200–25200)	81·9 (49·6–122)	31100 (18400–47300)	83·8 (50·7–126)	83·9 (67·5–101)	2·41 (-5·02–9·13)
**Sub-Saharan Africa**	**28300 (16500–44000)**	**23·8 (13·8–36·9)**	**46100 (26600–72000)**	**23·8 (13·7–36·8)**	**63·0 (59·8–66·2)**	**0·341 (-1·05–1·76)**
Central sub-Saharan Africa	2490 (1430–3910)	21·7 (12·6–33·9)	4520 (2570–7240)	21·8 (12·6–34·1)	81·4 (66·9–96·6)	0·267 (-6·49–7·54)
Angola	459 (266–723)	21·5 (12·6–33·1)	951 (541–1490)	21·7 (12·4–33·6)	107 (84·4–132)	1·29 (-7·81–13·2)
Central African Republic	114 (65·5–184)	21·6 (12·6–34·5)	173 (97·9–275)	21·7 (12·4–34·0)	51·6 (33·9–69·3)	0·265 (-8·89–9·27)
Congo	116 (66·8–182)	22·1 (12·7–34·7)	241 (138–382)	22·0 (12·6–34·6)	108 (87·4–130)	-0·195 (-8·78–8·29)
Democratic Republic of the Congo	1720 (976–2720)	21·7 (12·5–34·1)	3020 (1710–4860)	21·8 (12·6–33·9)	75·0 (56·8–95·0)	0·0168 (-9·66–10·4)
Equatorial Guinea	19·9 (11·3–31·8)	21·9 (12·4–34·2)	39·0 (22·4–60·6)	22·3 (12·9–34·4)	95·9 (74·5–120)	1·44 (-8·69–12·7)
Gabon	60·1 (34·8–93·7)	22·3 (13·1–34·6)	100 (57·3–158)	22·3 (13·0–34·6)	67·2 (51·2–84·8)	-0·102 (-8·39–9·51)
Eastern sub-Saharan Africa	9390 (5510–14600)	23·0 (13·3–35·8)	15900 (9170–24800)	23·0 (13·2–35·5)	69·7 (64·1–75·2)	-0·00958 (-2·85–3·02)
Burundi	233 (134–357)	22·1 (12·7–34·2)	465 (263–743)	22·0 (12·7–34·5)	99·8 (81·7–122)	-0·552 (-8·45–8·94)
Comoros	28·2 (16·4–43·9)	22·2 (13·0–34·3)	45·6 (26·0–70·6)	22·1 (12·6–34·3)	61·6 (45·0–78·6)	-0·422 (-10·3–9·40)
Djibouti	22·3 (12·6–34·7)	22·3 (12·9–34·9)	64·1 (36·1–102)	22·3 (12·7–34·8)	187 (160–222)	0·0294 (-9·21–10·3)
Eritrea	97·1 (54·2–155)	22·0 (12·9–34·4)	202 (113–322)	21·8 (12·7–34·2)	108 (86·6–131)	-0·780 (-9·88–9·05)

Ethiopia	2860 (1640–4480)	23·2 (13·5–36·1)	4470 (2620–6990)	23·1 (13·6–35·8)	56·3 (42·1–69·6)	-0·553 (-8·55–7·15)
Kenya	1280 (745–2010)	27·1 (15·8–42·2)	2510 (1460–3940)	27·0 (15·6–42·0)	96·8 (91·5–102)	-0·337 (-2·27–1·60)
Madagascar	567 (327–905)	22·1 (12·8–35·2)	1010 (567–1590)	22·0 (12·8–34·6)	78·2 (60·3–100)	-0·190 (-9·52–10·7)
Malawi	405 (233–635)	22·0 (12·7–34·3)	630 (365–1000)	21·9 (12·9–34·0)	55·8 (41·6–73·6)	-0·260 (-9·10–10·8)
Mozambique	641 (366–1010)	21·7 (12·5–34·0)	938 (540–1480)	21·8 (12·5–34·1)	46·3 (31·5–62·6)	0·154 (-9·49–10·1)
Rwanda	230 (134–359)	22·0 (13·0–33·8)	500 (291–791)	21·9 (12·8–34·0)	118 (95·3–143)	-0·533 (-10·2–9·60)
Somalia	277 (159–435)	22·2 (12·8–34·2)	519 (290–820)	22·1 (12·6–34·6)	87·3 (69·1–107)	-0·210 (-7·93–8·81)
South Sudan	291 (172–455)	21·8 (12·9–34·0)	399 (234–623)	21·8 (12·9–34·0)	36·8 (23·9–52·3)	0·237 (-8·74–10·6)
Uganda	733 (422–1160)	22·2 (12·8–34·8)	1200 (686–1890)	22·1 (12·8–34·5)	64·1 (47·8–80·6)	-0·455 (-9·29–8·99)
Tanzania	1370 (770–2150)	22·0 (12·6–34·2)	2350 (1330–3720)	22·0 (12·4–34·3)	72·1 (55·6–91·7)	0·188 (-9·45–11·3)
Zambia	354 (201–552)	22·3 (12·8–34·9)	611 (349–958)	22·3 (12·8–35·0)	72·2 (55·6–91·4)	0·230 (-8·87–9·72)
Southern sub-Saharan Africa	4670 (2710–7390)	34·1 (19·7–53·5)	7640 (4500–12000)	34·6 (20·2–53·5)	63·5 (58·6–69·3)	1·60 (-1·17–4·82)
Botswana	124 (71·3–193)	42·1 (24·3–65·1)	222 (127–344)	42·4 (24·3–65·6)	78·7 (63·8–96·3)	0·708 (-7·05–9·35)
Eswatini	50·1 (29·1–78·6)	33·9 (19·9–53·3)	70·5 (40·5–110)	34·6 (20·0–53·9)	40·7 (27·1–55·3)	2·00 (-6·74–12·5)

Lesotho	139 (79·6–219)	32·8 (18·9–50·9)	156 (90·2–245)	33·7 (19·8–52·5)	11·7 (2·03–22·0)	2·75 (-5·20–11·4)
Namibia	129 (74·7–200)	32·6 (18·9–50·8)	175 (101–279)	32·2 (18·6–50·2)	36·4 (23·6–49·6)	-1·25 (-10·2–8·11)
South Africa	3540 (2060–5620)	34·3 (19·9–54·0)	6180 (3620–9730)	34·8 (20·2–53·8)	74·2 (68·3–81·6)	1·38 (-2·12–4·94)
Zimbabwe	685 (391–1090)	32·3 (18·6–50·5)	841 (488–1330)	32·6 (19·0–51·3)	22·7 (11·9–35·0)	0·850 (-7·59–10·3)
Western sub-Saharan Africa	11700 (6800–18300)	22·1 (12·8–34·2)	18000 (10400–28200)	22·2 (12·7–34·3)	53·5 (50·2–57·6)	0·276 (-1·45–2·34)
Benin	240 (140–381)	21·5 (12·7–34·0)	446 (256–696)	21·7 (12·5–33·9)	85·6 (67·5–107)	0·740 (-8·92–11·1)
Burkina Faso	513 (294–795)	21·5 (12·4–33·4)	812 (475–1280)	21·7 (12·5–33·6)	58·2 (43·1–74·1)	1·18 (-7·94–10·4)
Cabo Verde	24·2 (13·9–38·1)	21·9 (12·5–34·4)	36·9 (21·2–58·1)	21·9 (12·6–34·7)	52·5 (35·5–73·1)	0·224 (-8·38–9·43)
Cameroon	593 (334–943)	21·6 (12·4–34·1)	1140 (657–1810)	22·0 (12·7–34·5)	91·9 (73·1–112)	1·57 (-7·36–11·1)
Chad	340 (196–531)	21·4 (12·4–33·4)	608 (347–952)	21·7 (12·4–33·9)	78·7 (61·6–96·8)	1·10 (-7·99–10·2)
Côte d'Ivoire	590 (339–935)	21·8 (12·6–34·4)	1060 (596–1680)	21·8 (12·5–33·9)	80·2 (63·0–101)	0·212 (-8·37–10·2)
The Gambia	55·1 (30·9–87·0)	21·6 (12·1–34·0)	92·0 (51·7–147)	21·6 (12·3–34·5)	67·0 (50·2–84·2)	-0·210 (-9·52–9·18)
Ghana	846 (475–1340)	21·9 (12·5–34·5)	1420 (800–2210)	22·1 (12·7–34·1)	67·9 (51·4–88·3)	1·04 (-8·10–12·0)
Guinea	448 (258–705)	21·7 (12·5–33·7)	570 (324–888)	21·6 (12·4–33·6)	27·2 (14·3–40·1)	-0·211 (-9·24–9·51)

Guinea-Bissau	41·7 (24·4–66·1)	21·9 (12·8–34·4)	62·6 (36·1–99·4)	21·9 (12·6–34·8)	50·0 (34·3–66·2)	0·0311 (-9·65–9·90)
Liberia	136 (79·1–213)	21·5 (12·6–33·5)	197 (114–309)	21·5 (12·6–34·0)	45·2 (30·2–61·3)	0·112 (-9·13–9·64)
Mali	529 (306–839)	21·6 (12·5–34·3)	893 (505–1390)	21·7 (12·5–33·7)	68·9 (52·2–87·4)	0·464 (-8·73–10·8)
Mauritania	124 (72·4–195)	22·0 (12·9–34·4)	220 (127–344)	22·0 (12·7–34·1)	76·6 (59·5–93·6)	0·00648 (-9·38–8·87)
Niger	376 (212–595)	21·6 (12·5–34·0)	760 (437–1220)	21·8 (12·6–34·1)	102 (82·6–125)	0·911 (-7·98–10·8)
Nigeria	6070 (3520–9510)	22·6 (13·2–35·0)	8310 (4840–13000)	22·6 (13·1–34·9)	36·8 (33·1–40·9)	0·164 (-2·01–2·23)
São Tomé and Príncipe	7·24 (4·16–11·5)	21·5 (12·4–33·8)	9·76 (5·59–15·4)	21·4 (12·3–33·8)	34·9 (20·5–52·0)	-0·359 (-10·0–10·6)
Senegal	440 (252–690)	21·7 (12·6–34·0)	740 (427–1150)	21·8 (12·6–34·2)	68·2 (50·3–86·8)	0·631 (-9·34–11·1)
Sierra Leone	216 (125–341)	21·4 (12·5–33·6)	356 (200–556)	21·7 (12·4–33·8)	64·6 (48·5–82·8)	1·20 (-8·40–11·1)
Togo	149 (84·8–235)	21·5 (12·6–33·5)	296 (169–467)	21·7 (12·5–34·1)	99·2 (78·9–121)	0·721 (-7·92–10·5)

Table 6. Guideline for Accurate and Transparent Health Estimates Reporting (GATHER) checklist.

Item #	Checklist item	Reported on page #
**Objectives and funding**		
**1**	Define the indicator(s), populations (including age, sex, and geographic entities), and time period(s) for which estimates were made.	Age: p 11<br>Sex: NR<br>Locations: Appendix pp 1-7<br>Time: p 2, p 4
**2**	List the funding sources for the work.	p 3
**Data Inputs**		
For all data inputs from multiple sources that are synthesized as part of the study:		
**3**	Describe how the data were identified and how the data were accessed.	p 9
**4**	Specify the inclusion and exclusion criteria. Identify all ad-hoc exclusions.	p 10
**5**	Provide information on all included data sources and their main characteristics. For each data source used, report reference information or contact name/institution, population represented, data collection method, year(s) of data collection, sex and age range, diagnostic criteria or measurement method, and sample size, as relevant.	p 9<br>Appendix Table S3<br>Global Health Data Exchange (GHDx, http://ghdx.healthdata.org/)
**6**	Identify and describe any categories of input data that have potentially important biases (e.g., based on characteristics listed in item 5).	pp 9-10
For data inputs that contribute to the analysis but were not synthesized as part of the study:		
**7**	Describe and give sources for any other data inputs.	pp 11-13
For all data inputs:		
**8**	Provide all data inputs in a file format from which data can be efficiently extracted (e.g., a spreadsheet rather than a PDF), including all relevant meta-data listed in item 5. For any data inputs that cannot be shared because of ethical or legal reasons, such as third-party ownership, provide a contact name or the name of the institution that retains the right to the data.	Global Health Data Exchange (GHDx, http://ghdx.healthdata.org/)<br>Epi Visualization | Viz Hub (http://ihmeuw.org/5u6w)
**Data analysis**		
**9**	Provide a conceptual overview of the data analysis method. A diagram may be helpful.	p 8<br>Reference 41

**10**	Provide a detailed description of all steps of the analysis, including mathematical formulae. This description should cover, as relevant, data cleaning, data pre-processing, data adjustments and weighting of data sources, and mathematical or statistical model(s).	pp 9-13<br>Reference 41
**11**	Describe how candidate models were evaluated and how the final model(s) were selected.	p 11
**12**	Provide the results of an evaluation of model performance, if done, as well as the results of any relevant sensitivity analysis.	Reference 41, 49
**13**	Describe methods for calculating uncertainty of the estimates. State which sources of uncertainty were, and were not, accounted for in the uncertainty analysis.	p 13<br>p 20
**14**	State how analytic or statistical source code used to generate estimates can be accessed.	Global Health Data Exchange (GHDx, https://ghdx.healthdata.org/gbd-2019/code)
**Results and Discussion**		
**15**	Provide published estimates in a file format from which data can be efficiently extracted.	Epi Visualization | Viz Hub (http://ihmeuw.org/5u6w)<br>GBD Compare | Viz Hub (http://ihmeuw.org/5u6z)
**16**	Report a quantitative measure of the uncertainty of the estimates (e.g. uncertainty intervals).	95% uncertainty intervals provided for all reported estimates in text and tables
**17**	Interpret results in light of existing evidence. If updating a previous set of estimates, describe the reasons for changes in estimates.	pp 3-5<br>pp 16-18
**18**	Discuss limitations of the estimates. Include a discussion of any modelling assumptions or data limitations that affect interpretation of the estimates.	pp 20-22

AUTHOR CONTRIBUTIONS

Managing the overall research enterprise

Theo Vos and M Ashworth Dirac

Writing the first draft of the manuscript

Atalel Fentahun Awedew, Hannah Han, M Ashworth Dirac

Primary responsibility for applying analytical methods to produce estimates

Hannah Han

Primary responsibility for seeking, cataloguing, extracting, or cleaning data; designing or coding figures and tables

Hannah Han

Providing data or critical feedback on data sources

Muktar Beshir Ahmed, Omar Almidani, Erfan Amini, Jalal Arabloo, Ayele Mamo Argaw, Seyyed Shamsadin Athari, Maciej Banach, Amadou Barrow, Akshaya Srikanth Bhagavathula, Boris Bikbov, Belay Boda Abule Bodicha, Nadeem Shafique Butt, Xiaochen Dai, Linh Phuong Doan, Sahar Eftekharzadeh, Ali Fatehizadeh, Tushar Garg, Teferi Gebru Gebremeskel, Mahaveer Golechha, Vivek Kumar Gupta, Mehdi Hosseinzadeh, Nahlah Elkudssiah Ismail, Mihajlo Jakovljevic, Shubha Jayaram, Seyed Behzad Jazayeri, Yousef Saleh Khader, Sang-woong Lee, Shaun Wen Huey Lee, Narges Malih, Entezar Mehrabi Nasab, Gedefaye Nibret Mihrtie, Awoke Temesgen Misganaw, Ali H Mokdad, Mariam Molokhia, Sreenivas Narasimha Swamy, Son Hoang Nguyen, Mayowa O Owolabi, Shrikant Pawar, Salman Rawaf, Reza Rawassizadeh, Jasvinder A Singh, Ker-Kan Tan, Gebremaryam Temesgen, Marcos Roberto Tovani-Palone, Sahel Valadan Tahbaz, Bay Vo, Linh Gia Vu, Vahit Yiğit, Ismaeel Yunusa

Developing methods or computational machinery

Muktar Beshir Ahmed, Akshaya Srikanth Bhagavathula, Xiaochen Dai, Ali Fatehizadeh, Simon I Hay, Mehdi Hosseinzadeh, Sang-woong Lee, Ali H Mokdad, Reza Rawassizadeh, Bay Vo

Providing critical feedback on methods or results

Behzad Abbasi, Muktar Beshir Ahmed, Omar Almidani, Erfan Amini, Jalal Arabloo, Seyyed Shamsadin Athari, Daniel Atlaw, Maciej Banach, Amadou Barrow, Akshaya Srikanth Bhagavathula, Vijayalakshmi S Bhojaraja, Boris Bikbov, Belay Boda Abule Bodicha, Nadeem Shafique Butt, Florentino Luciano Caetano dos Santos, Omid Dadras, Xiaochen Dai, Linh Phuong Doan, Sahar Eftekharzadeh, Ali Fatehizadeh, Tushar Garg, Teferi Gebru Gebremeskel, Motuma Erena Getachew, Syed Amir Gilani, Mahaveer Golechha, Vivek Kumar Gupta, Mohammad-Salar Hosseini, Mehdi Hosseinzadeh, Ayesha Humayun, Irena M Ilic, Milena D Ilic, Nahlah Elkudssiah Ismail, Mihajlo Jakovljevic, Shubha Jayaram, Seyed Behzad

Jazayeri, Alelign Tasew Jema, Ibraheem M Karaye, Yousef Saleh Khader, Ejaz Ahmad Khan, Sang-woong Lee, Stephen S Lim, Stany W Lobo, Azeem Majeed, Mohammad-Reza Malekpour, Narges Malih, Ahmad Azam Malik, Entezar Mehrabi Nasab, Tomislav Mestrovic, Irmina Maria Michalek, Gedefaye Nibret Mihrtie, Mohammad Mirza-Aghazadeh-Attari, Awoke Temesgen Misganaw, Ali H Mokdad, Mariam Molokhia, Sreenivas Narasimha Swamy, Son Hoang Nguyen, Ali Nowroozi, Mayowa O Owolabi, Shrikant Pawar, David Laith Rawaf, Salman Rawaf, Reza Rawassizadeh, Chethan Sampath, Jeevan K Shetty, Migbar Mekonnen Sibhat, Jasvinder A Singh, Ker-Kan Tan, Gebremaryam Temesgen, Musliu Adetola Tolani, Marcos Roberto Tovani-Palone, Sahel Valadan Tahbaz, Rohollah Valizadeh, Bay Vo, Linh Gia Vu, Lin Yang, Fereshteh Yazdanpanah, Arzu Yigit, Vahit Yiğit, Ismaeel Yunusa, Mazyar Zahir

Drafting the work or revising it critically for important intellectual content

Behzad Abbasi, Mohsen Abbasi-Kangevari, Muktar Beshir Ahmed, Omar Almidani, Erfan Amini, Jalal Arabloo, Daniel Atlaw, Maciej Banach, Amadou Barrow, Akshaya Srikanth Bhagavathula, Vijayalakshmi S Bhojaraja, Boris Bikbov, Belay Boda Abule Bodicha, Florentino Luciano Caetano dos Santos, Linh Phuong Doan, Sahar Eftekharzadeh, Ali Fatehizadeh, Tushar Garg, Motuma Erena Getachew, Seyyed-Hadi Ghamari, Veer Bala Gupta, Vivek Kumar Gupta, Simon I Hay, Mohammad-Salar Hosseini, Irena M Ilic, Milena D Ilic, Nahlah Elkudssiah Ismail, Mihajlo Jakovljevic, Shubha Jayaram, Alelign Tasew Jema, Ali Kabir, Yousef Saleh Khader, Ejaz Ahmad Khan, Iván Landires, Shaun Wen Huey Lee, Azeem Majeed, Mohammad-Reza Malekpour, Narges Malih, Ahmad Azam Malik, Tomislav Mestrovic, Irmina Maria Michalek, Mohammad Mirza-Aghazadeh-Attari, Ali H Mokdad, Mariam Molokhia, Sreenivas Narasimha Swamy, Son Hoang Nguyen, Ali Nowroozi, Virginia Nuñez-Samudio, Mayowa O Owolabi, Shrikant Pawar, Norberto Perico, David Laith Rawaf, Salman Rawaf, Giuseppe Remuzzi, Amirhossein Sahebkar, Jeevan K Shetty, Jasvinder A Singh, Ker-Kan Tan, Musliu Adetola Tolani, Marcos Roberto Tovani-Palone, Sahel Valadan Tahbaz, Linh Gia Vu, Lin Yang, Arzu Yigit, Vahit Yiğit, Mazyar Zahir
